# Incidence and temporal trends in type 2 diabetes by weight status: A systematic review and meta-analysis of prospective cohort studies

**DOI:** 10.7189/jogh.13.04088

**Published:** 2023-09-01

**Authors:** Hong-jie Yu, Mandy Ho, Xiangxiang Liu, Jundi Yang, Pui Hing Chau, Daniel Yee Tak Fong

**Affiliations:** 1 School of Nursing, University of Hong Kong, Hong Kong SAR, China; 2 National Clinical Research Center for Infectious Diseases, The Third People’s Hospital of Shenzhen, Shenzhen, China

## Abstract

**Background:**

Diabetes is more prevalent among overweight/obese individuals, but has become a significant public health challenge among normal weight populations. In this meta-analysis, we aimed to estimate diabetes/prediabetes incidence and its temporal trends by weight status.

**Methods:**

PubMed, Embase, Web of Science, and Cochrane Library were searched until 8 December 2021. Prospective cohort studies reporting diabetes incidence by baseline body mass index (BMI) categories in adults were included. The median year of data collection was used to assess the temporal trends. Subgroup analyses and meta-regression were also performed.

**Results:**

We included 94 studies involving 3.4 million adults from 22 countries. The pooled diabetes incidence in underweight, normal-weight, and overweight/obese adults was 4.5 (95% confidence interval (CI) = 2.8-7.3), 2.7 (95% CI = 2.2-3.3), and 10.5 (95% CI = 9.3-11.8) per 1000 person-years, respectively. The diabetes incidence in low- and middle-income countries (LMICs) was higher than in high-income countries among normal-weight (5.8 vs 2.0 per 1000 person-years) or overweight/obese (15.9 vs 8.9 per 1000 person-years) adults. European and American regions had a higher diabetes incidence than the non-Western areas, regardless of weight status. Underweight diabetes incidence decreased significantly from 1995-2000 to 2005-2010. Diabetes incidence in normal-weight populations has increased continuously since 1985 by an estimated 36% every five years. In overweight/obese adults, diabetes incidence increased between 1985-1990 and 1995-2000, stabilised between 2000 and 2010, and spiked suddenly after 2010.

**Conclusions:**

Diabetes incidence and its temporal trends differed by weight status. The continuous upward trend of diabetes incidence among overweight/obese individuals requires urgent attention, particularly in LMICs. Furthermore, diabetes among normal-weight individuals is becoming a significant public health problem.

**Registration:**

PROSPERO (CRD42020215957).

Obesity is a known risk factor for diabetes [1]. However, studies have suggested that diabetes in non-overweight individuals is becoming a significant public health challenge worldwide, particularly in Asian countries [2-5]. Likewise, a substantial proportion of individuals with normal weight also develop diabetes [3,4]. Results of trend analyses using data from nationally representative surveys reported that diabetes prevalence remains stable among adults with normal weight and overweight, while it increased and then dropped before leveling out among individuals with obesity over the past two decades [6-8]. However, prevalence is a less reliable metric than incidence in assessing changes in population risk for diabetes because increasing prevalence might be attributed to several factors such as increasing incidence, improved survival, and/or lower mortality rates [9-11]. To date, only one systematic review and a multicounty trend analysis in high- and middle-income settings has described the trends in diabetes incidence [10]. However, it did not provide data to quantify diabetes incidence and its temporal trends by weight status, which are of great importance for public health policies and clinical practices.

Additionally, the prevalence of prediabetes is nearly three times higher than that of diabetes, and pre-diabetes plays a critical role in the pathophysiology of type 2 diabetes (hereinafter referred to as diabetes) [12,13]. Thus, we conducted a systematic review and meta-analysis to estimate the incidence of prediabetes and diabetes among adults with underweight, normal weight, and overweight/obesity and their temporal trends.

## METHODS

We followed the Preferred Reporting Items for Systematic Reviews and Meta-Analyses (PRISMA guidelines) [14] in conducting this systematic review and meta-analysis, and we preregistered its protocol in the International prospective register of systematic reviews (PROSPERO, CRD42020215957). We used the population, interventions/exposures, comparators, outcomes, and study design (PICOS) framework to develop the search strategy. We defined the population as adults (18 years and above) without diabetes at baseline, the intervention/exposures as different weight statuses defined by body mass index (BMI), outcomes as the incidence of prediabetes or diabetes, and we limited the study design to prospective cohort studies. The “comparator” criteria was not applicable in our review. We adapted the search strategy from a previous meta-analysis [15]. We searched PubMed, Embase, Web of Science, and Cochrane Library from inception to 8 December 2021 using keywords such as “diabetes”, “prediabetes”, “body mass index or BMI”, “obese”, “non-obese”, “cohort study”, and “adult”. We filtered the search results to “English” and “Human” studies (Table S1 in the **Online Supplementary Document**). We also identified additional studies from the reference list of two previous related meta-analyses [16,17].

We included prospective cohort studies with a follow-up duration >12 months, participants ≥18 years and free of prediabetes or diabetes at baseline, and with data on the cases and follow-up duration, incidence rate (cases/person-years), or cumulative incidence (%) by baseline BMI categories. We also included eligible studies from the same cohort but with different follow-up durations. For eligible studies from the same cohort and with a same follow-up duration, we only retained the one with a larger sample size. We excluded studies that targeted children or individuals with preexisting diabetes and related cardiovascular diseases and nerve, kidney, and eye damage and studies that combined prediabetes and diabetes as the main outcome without stratified analysis or measured type 1 or gestational diabetes as the main outcome. We defined prediabetes/diabetes as per the International Diabetes Federation [18] and American Diabetes Association [19].

Three authors (YHJ, LXX, and YJD) screened the title, abstract, and full text independently. Two authors (YHJ and LXX) extracted and checked the study characteristics, participants’ characteristics, exposure and outcome assessment, and baseline BMI categories using a standardised data extraction form. Two authors (YHJ and YJD) assessed study quality using the Newcastle-Ottawa scale (NOS) (Table S2 in the **Online Supplementary Document**) [20]; studies with a NOS score ≥7 were deemed good quality. We resolved all uncertainties through group discussion.

### Data analysis

The primary outcome was the incidence rate of prediabetes or diabetes by weight status, calculated from the incident cases and corresponding person-years of follow-up, or as the cumulative incidence (%) divided by the median/average follow-up duration, if available [21]. We defined weight status by the ethnic-specific BMI classification recommended by World Health Organization (WHO): BMI (kg/m^2^)<18.5 as underweight, 18.5-22.9 for Asians and 18.5-24.9 for non-Asians as normal weight, ≥23 for Asians and ≥25 for non-Asians as overweight/obesity, respectively [22], with a margin of ±1 kg/m^2^ [23]. We calculated pooled estimates using the DerSimonian-Laird method [24] and used the Cochran’s Q test and *I*^2^ to assess statistical between-study heterogeneity [25].

We conducted subgroup analyses of pre-specified factors to explore potential sources of heterogeneity, including sex (female, male, and total), age (≤45, 45-60, and ≥60 years), country income evaluated per World Bank definitions (low- and middle-income countries (LMICs), and high-income countries (HICs)) [26], WHO region (Western Pacific Region, Region of the Americas, European Region, South-East Asia Region, and Eastern Mediterranean Region) [27], median follow-up duration (≤8 and >8 years), median year of data collection (before 1985, five-year interval between 1985 and 2010, and after 2010), study setting (rural, urban, and mixed), weight assessment (self-reported and directly measured), and prediabetes/diabetes ascertainment (via blood test, medical records, self-reported, and multiple methods). We defined “multiple methods” by blood test plus other one or two ascertainment methods.

We used the median year of data collection reported by the study to estimate temporal trends [28-30] in three steps: by plotting the pooled incidence of diabetes/prediabetes against the median year of data collection in five-year intervals, by conducting a univariate meta-regression analysis with the median year of data collection as a continuous covariate and obtaining a bubble plot, and by conducting a multivariate meta-regression analysis to examine the robustness of temporal trends after including pre-specified factors with *P* ≤ 0.20 in their respective univariate meta-regression analysis [31]. We used the *R*^2^ to quantify the variance proportion explained by each model. We computed the correlation matrix and variance inflation factor (VIF) to assess the potential multicollinearity between pre-specified factors; we omitted one of two factors with correlation coefficients >0.5 or VIF>3.0 in the multivariate meta-regression model [32].

We conducted sensitivity analyses via the leave-one-out method, which re-pooled the incidence after omitting every single study to detect its contribution to overall heterogeneity [33]. Moreover, we repeated the meta-regression by only including good quality studies (NOS≥7) to test for robustness of results. We assessed publication bias via visual inspection of the funnel plot of standard error and Egger regression test when ≥10 incidences were available [25]. We performed all analyses using R, version 4.0.3. (R Core Team, Auckland, New Zealand) and its “metafor” and “meta” packages. We considered a two-tailed *P*-value <0.05 statistically significant.

### Patient and public involvement

This systematic review and meta-analysis did not involve any raw personal data, so a patient and public involvement statement is not applicable.

## RESULTS

### Study characteristics

We retrieved 26 920 studies, of which 94 [34-127] met the inclusion criteria (Figure S1 in the **Online Supplementary Document**). Ninety-two studies provided information on diabetes incidence (Table S3 in the **Online Supplementary Document**) [71-115,118-127]. We excluded one study from the meta-analysis because its BMI was categorised by quintiles without a clear description of the range [115]. The included studies covered 22 countries/regions and were published between 1991 and 2021, involving 3.4 million adults at baseline and observing about 184 000 cases during a median follow-up of eight years. The NOS score ranged from four to nine, and 78.7% (70/94) of studies were good quality (NOS≥7) (Table S2 in the **Online Supplementary Document**), with quality increasing with publication year.

### Diabetes incidence by weight status

**Figure 1:**
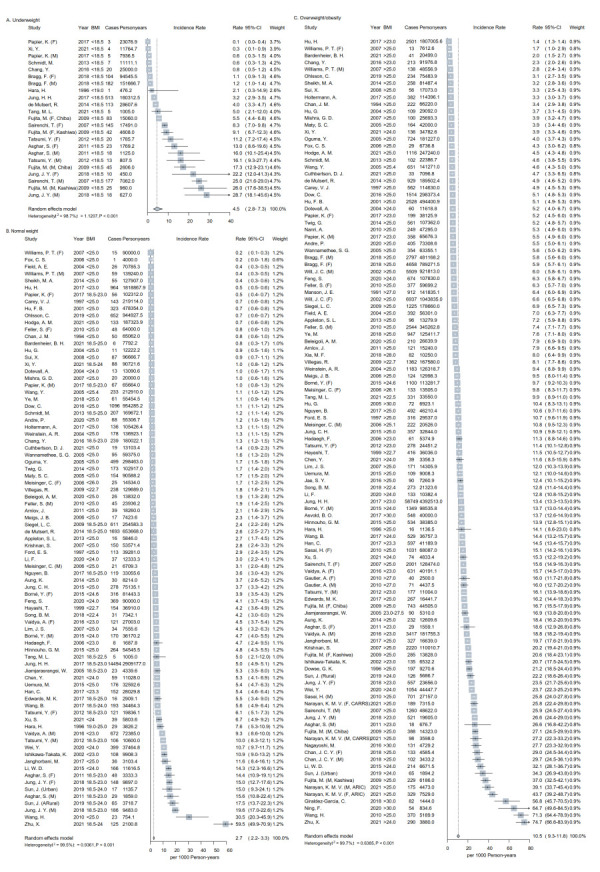
The pooled incidence of diabetes by baseline weight status. **Panel A.** Underweight. **Panel B.** Normal weight. **Panel C.** Overweight/obesity. BMI – body mass index (kg/m^2^), F – female, M – male.

Fourteen studies [36,41,45,46,56,61,74,75,91,92,94,100,124,125] reported 23 estimates of diabetes incidences among adults with underweight. During a median follow-up of eight years (interquartile range (IQR) = 5-10), 1576 cases were identified. The pooled diabetes incidence among adults with underweight was 4.5 (95% confidence interval (CI) 2.8-7.3; *I*^2^ = 99.7%) cases per 1000 person-years (**Figure 1**, panel A). Among 73 studies that reported 83 estimates of diabetes incidence in adults with normal weight [34-36,38-40,42,44-48,50-55,59-65,67-69,71-79,81-84,87,89-91,94-110,112,114], 31 304 cases were identified during a median follow-up of eight years (IQR = 5-11.2). The pooled diabetes incidence in adults with normal weight was 2.7 (95% CI = 2.2-3.3; *I*^2^ = 99.5%) cases per 1000 person-years (**Figure 1**, panel B). Diabetes incidence among individuals with overweight/obesity was available in 91 studies [34-114] and the pooled value was 10.5 (95% CI = 9.3-11.8; *I*^2^ = 99.7%) per 1000 person-years (**Figure 1**, panel C). The leave-one-out sensitivity analyses did not indicate the predominance of any single study for overall heterogeneity (Table S4 in the **Online Supplementary Document**). After excluding studies with NOS<7, the pooled incidences of diabetes in adults with underweight, normal weight, and overweight/obesity were 5.2 (3.1-8.8, *I*^2^ = 98.9%, n = 11 studies), 3.3 (2.6-4.3, *I*^2^ = 99.5%, n = 55 studies), and 11.5 (10.0-13.2, *I*^2^ = 99.7%, n = 69 studies) cases per 1000 person-years, respectively (Figure S2, panels A-C in the **Online Supplementary Document**).

### Subgroup and meta-regression analysis of diabetes incidence

Subgroup (**Table 1**) and univariate meta-regression (**Table 2**) analyses indicated that diabetes incidence (per 1000 person-years) varied by age (older adults had a higher diabetes incidence), weight assessment (direct measurement had a higher diabetes incidence than self-report), and diabetes ascertainment methods (blood test and multiple had a higher diabetes incidence than medical records and self-report). Additionally, diabetes incidence in LMICs was significantly lower than that in HICs for adults with underweight (1.7 vs 7.6, odds ratio (OR) = 0.23; 95% CI = 0.09-0.56), while diabetes incidence in LMICs was significantly higher than that in HICs for adults with normal weight (5.8 vs 2.0, OR = 2.95; 95% CI = 1.87-4.54) and overweight/obesity (15.9 vs 8.9, OR = 1.78; 95% CI = 1.35-2.33). Compared with the Western Pacific, the Americas and European had a significantly lower diabetes incidence, while the South-East Asian Region and Eastern Mediterranean Region had similar diabetes incidences in adults with normal weight and overweight/obesity. Among the studies that reported sex-specific diabetes incidence in adults with normal weight, diabetes incidence did not differ between males and females. Studies with a follow-up duration ≤8 years reported a higher diabetes incidence (12.0, 95% CI = 10.2-14.2) than those with >8 years of follow-up (8.6, 95% CI = 7.6-9.8) for only overweight/obese adults.

**Table 1:** Subgroup analysis of diabetes incidence by different weight status*

	Underweight (n = 14 articles)					Normal weight (n = 73 articles)					Overweight/obesity (n = 91 articles)				
**Subgroups**	**Number of estimates**	**Incidence rate**	**95% CI**	***I* ^2^**	***P*-value for comparison**	**Number of estimates**	**Incidence rate**	**95% CI**	***I* ^2^**	***P*-value for comparison**	**Number of estimates**	**Incidence rate**	**95% CI**	***I* ^2^**	***P*-value for comparison**
**All**	23	4.5	2.8-7.3	98.7%		83	2.7	2.2-3.3	99.5%		113	10.5	9.3-11.8	99.7%	
Sex					0.07					0.002					0.38
*Female*	8	5.0	2.3-10.6	98.2%		20	1.4	1.0-2.2	99.2%		30	9.3	7.6-11.3	99.6%	
*Male*	10	6.7	2.6-17.5	99.2%		22	2.5	1.7-3.8	99.5%		33	10.3	8.2-12.9	99.7%	
*Mixed*	5	1.6	0.6-4.0	93.0%		41	3.7	2.8-5.0	99.5%		50	11.5	9.1-14.4	99.6%	
Age					0.45					0.003					0.23
*≤45*	5	2.1	0.4-9.9	97.6%		19	1.5	1.0-2.3	99.1%		27	8.4	6.2-11.5	99.8%	
*45-60*	16	5.8	3.3-10.1	99.0%		58	3.1	2.5-3.8	99.4%		76	11.3	10.0-12.7	99.6%	
*≥60*	2	4.3	1.9-9.6	0.0%		6	4.0	2.1-7.6	99.5%		10	11.0	7.3-16.5	99.6%	
World Bank country†					0.002					<0.001					<0.001
*High income*	15	7.6	4.6-12.6	98.5%		60	2.0	1.6-2.5	99.6%		81	8.9	7.7-10.3	99.8%	
*Low- and middle-income*	8	1.7	0.8-3.8	97.4%		18	5.8	3.9-8.7	99.1%		32	15.9	12.4-20.3	99.6%	
WHO Region					<0.001					<0.001					<0.001
*Western Pacific*	16	6.1	3.4-11.0	96.9%		33	4.8	3.6-6.7	99.6%		49	13.3	11.0-16.2	99.8%	
*Americas*	2	3.9	3.3-4.7	0.0%		24	1.6	1.2-2.1	98.5%		28	7.2	5.9-8.8	99.6%	
*European*	1	0.6	0.3-1.3	–		18	1.5	1.1-2.2	98.9%		23	7.8	6.3-9.6	99.1%	
*South-East Asia*	4	2.2	0.4-12.6	96.9%		5	3.7	0.9-14.9	99.1%		10	18.9	10.7-33.6	98.3%	
*Eastern Mediterranean*						3	4.5	1.1-18.8	98.3%		3	10.5	4.0-27.8	99.5%	
Follow-up duration in years‡					0.51					0.98					0.002
*≤8*	12	3.9	2.0-7.6	98.7%		47	2.7	2.0-3.6	99.5%		67	12.0	10.2-14.2	99.7%	
*>8*	11	5.4	2.6-10.9	98.5%		36	2.7	2.0-3.5	99.3%		46	8.6	7.6-9.8	99.4%	
Median year of data collection					<0.001					<0.001					<0.001
*≤1985*	1	2.1	0.3-14.9			7	1.9	1.1-3.2	98.7%		11	6.6	5.6-7.8	98.7%	
*1985-1990*						9	1.2	0.7-2.1	98.2%		9	5.7	4.2-7.8	98.8%	
*1990-1995*	1	0.6	0.3-1.3			6	1.7	1.2-2.4	97.4%		7	6.6	4.8-9.0	98.8%	
*1995-2000*	7	10.4	5.8-18.9	98.0%		16	2.1	1.3-3.2	98.9%		24	11.1	8.4-14.6	99.7%	
*2000-2005*	5	7.9	3.5-18.0	94.3%		12	3.2	1.9-5.3	99.2%		18	11.9	8.9-15.8	99.3%	
*2005-2010*	8	2.1	1.1-4.0	98.3%		19	3.4	2.5-4.7	99.2%		24	11.0	8.9-13.6	99.7%	
*>2010*	1	5.0	2.1-12.0			14	5.6	2.4-13.0	99.7%		20	16.4	8.9-30.3	99.9%	
Study setting					<0.001					<0.001					<0.001
*Rural*	4	13.6	10.9-17.1	98.7%		9	8.1	6.0-11.0	99.6%		9	15.1	13.3-17.2	85.5%	
*Urban*						2	5.2	0.7-40.5	98.6%		2	16.6	4.0-68.2	99.2%	
*Mixed*	4	5.2	1.6-17.0	98.8%		9	3.7	1.7-8.1	98.6%		14	14.4	10.3-20.2	99.6%	
Weight assessment					0.01					<0.001					<0.001
*Measured directly*	19	6.6	3.9-11.3	98.9%		62	3.8	2.9-4.8	99.6%		88	12.8	11.1-14.7	99.7%	
*Self-reported*	4	0.6	0.1-3.4	95.7%		21	1.0	0.8-1.3	98.7%		25	5.4	4.6-6.4	99.4%	
Diabetes ascertainment					<0.001					<0.001					<0.001
*Blood test*	12	10.9	6.7-17.7	96.9%		27	6.0	3.2-11.2	99.6%		46	16.9	11.9-24.1	99.8%	
*Multiple*§	2	12.9	9.1-18.3	3.6%		14	3.0	2.3-4.0	96.4%		16	9.0	7.4-10.9	97.8%	
*Medical records*	4	1.3	0.7-2.7	98.5%		8	1.8	0.9-3.7	99.8%		12	6.6	4.9-9.0	99.8%	
*Self-reported*	5	0.7	0.2-3.4	94.2%		34	1.5	1.1-2.0	99.2%		39	6.8	5.8-8.0	99.6%	

CI – confidence interval, WHO – World Health Organization

*We defined weight status by the ethnic-specific body mass index classification recommended by the WHO [22].

†We categorised country income by the World Bank Country and Lending Groups in 2019 [26].

‡We categorised follow-up by its median.

§We defined multiple as blood test plus other ascertainment method.

**Table 2:** Univariate meta-regression analysis of diabetes incidence by different weight status

Subgroups	Underweight (n = 14 articles)					Normal weight (n = 73 articles)					Overweight/obesity (n = 91 articles)				
	**Number of estimates**	**OR (95% CI)**	***P*-value**	**Variance explained *R^2^***	**Maximum likelihood test *P*-value**	**Number of estimates**	**OR (95% CI)**	***P*-value**	**Variance explained *R^2^***	**Maximum likelihood test *P*-value**	**Number of estimates**	**OR (95% CI)**	***P*-value**	**Variance explained *R^2^***	**Maximum likelihood test *P*-value**
Sex				0%	0.21				0%	0.002				0%	0.39
*Female*	8	Ref				20	Ref				30	Ref			
*Male*	10	1.40 (0.40-4.92)	0.60			22	1.76 (0.99-3.16)	0.06			33	1.11 (0.79-1.57)	0.54		
*Mixed*	5	0.32 (0.07-1.52)	0.15			41	2.58 (1.54-4.33)	<0.001			50	1.24 (0.91-1.71)	0.18		
Age	23	1.07 (1.02-1.11)	0.002	5.26%	0.002	83	1.04 (1.02-1.06)	0.002	22.15%	<0.001	113	1.01 (1.00-1.03)	0.03	7.69%	0.03
World Bank country†				21.49%	0.001				1.40%	<0.001				0%	<0.001
*High income*	15	Ref				60	Ref				81	Ref			
*Low- and middle-income*	8	0.23 (0.09-0.56)	0.001			18	2.95 (1.87-4.64)	<0.001			32	1.78 (1.35-2.33)	<0.001		
WHO Region				0%	0.19				15.11%	<0.001				0.82%	0.007
*Western Pacific*	16	Ref				33	Ref				49	Ref			
*Americas*	2	0.51 (0.07-3.52)	0.50			24	0.32 (0.19-0.54)	<0.001			28	0.54 (0.40-0.73)	<0.001		
*European*	1	0.10 (0.01-1.25)	0.07			18	0.32 (0.18-0.56)	<0.001			23	0.58 (0.42-0.8)	0.001		
*South-East Asia*	4	0.38 (0.10-1.49)	0.17			5	0.75 (0.30-1.92)	0.55			10	1.42 (0.92-2.2)	0.12		
*Eastern Mediterranean*						3	0.93 (0.28-3.04)	0.90			3	0.79 (0.37-1.67)	0.53		
Follow-up duration, years	23	0.95 (0.88-1.03)	0.24	0%	0.24	83	0.96 (0.94-0.99)	0.003	13.88%	0.003	113	0.96 (0.94-0.97)	<0.001	17.40%	<0.001
Weight assessment				0%	0.001				5.37%	<0.001				8.12%	<0.001
*Measured directly*	19	Ref				62	Ref				88	Ref			
*Self-reported*	4	0.10 (0.03-0.36)	0.001			21	0.27 (0.17-0.42)	<0.001			25	0.42 (0.32-0.55)	<0.001		
Diabetes ascertainment				49.86%	<0.001				0%	<0.001				0%	<0.001
*Blood test*	12	Ref				27	Ref				46	Ref			
*Multiple*‡	2	1.23 (0.35-4.27)	0.75			14	0.47 (0.24-0.93)	0.03			16	0.48 (0.33-0.72)	<0.001		
*Medical records*	4	0.12 (0.05-0.31)	<0.001			8	0.29 (0.13-0.67)	0.004			12	0.36 (0.23-0.56)	<0.001		
*Self-reported*	5	0.07 (0.03-0.19)	<0.001			34	0.24 (0.14-0.41)	<0.001			39	0.37 (0.28-0.50)	<0.001		

CI – confidence interval, OR – odds ratio, Ref – reference group, WHO – World Health Organization

*We defined weight status by the ethnic-specific body mass index classification recommended by the WHO [22].

†We categorised country income by the World Bank Country and Lending Groups in 2019 [26].

‡We defined multiple as blood test plus other ascertainment method.

### The temporal trends of diabetes incidence by weight status

**Figure 2:**
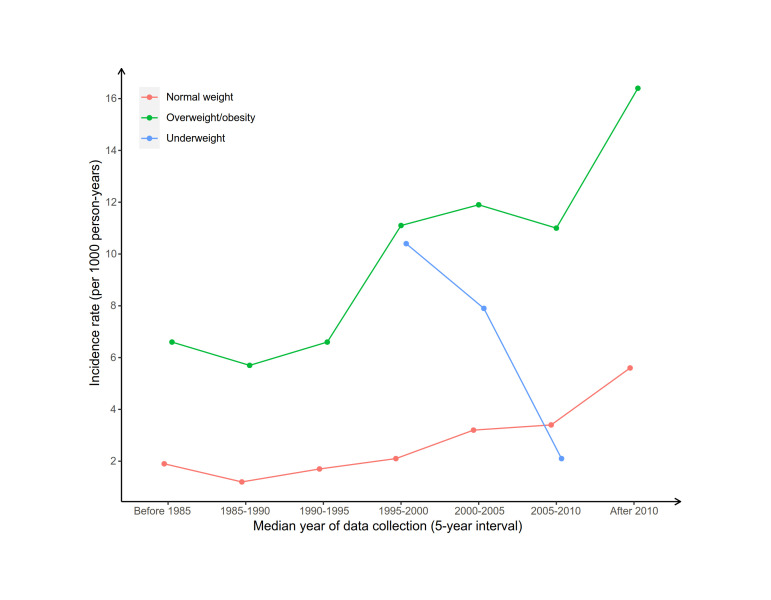
The temporal trends of diabetes incidence by baseline weight status. Blue line: Underweight. Red line: Normal weight. Green line: Overweight/obesity. The diabetes incidence was pooled by five years interval; median year of data collection was calculated by the mean value of baseline and follow-up year.

The pooled diabetes incidence (n = 14 studies) showed a sharp decrease since 1995 in adults with underweight (**Figure 2**). However, the diabetes incidence in adults with normal weight (n = 73 studies) increased continuously from 1.2 per 1000 person-years between 1985 and 1990 to 5.6 per 1000 person-years after 2010, with an estimated increase of 36% every five years. The pooled diabetes incidence in adults with overweight/obesity (n = 91 studies) showed a great increase from around six per 1000 person-years in 1985 to 11 per 1000 person-years between 1995 and 2000, remained stable between 2000 and 2010, but spiked to 16.4 per 1000 person-years after 2010. The bubble plot of diabetes incidence by median year of data collection indicated that the adjusted and unadjusted trends were significant across different weight statuses (Figure S3, panel A-C in the **Online Supplementary Document**). WHO region, country income, and diabetes ascertainment methods were not included as adjusting factors because of substantial correlation coefficients (>0.5) (Table S5 in the **Online Supplementary Document**). The results of sensitivity analyses by excluding studies with a NOS<7 in meta-regression analyses were similar to the primary analyses (Figure S4, panel A-C in the **Online Supplementary Document**).

### Prediabetes incidence by weight status

Three studies reported prediabetes and diabetes separately [103,113,121], while two reported hyperglycaemia (prediabetes combined with diabetes, but with diabetes cases only accounting for about 10%) (Table S6 in the **Online Supplementary Document**) [116,117]. All eligible studies were published between 2016 and 2021; they involved 93 910 adults and identified 19 758 cases during a median follow-up of 4.4 years (IQR = 3.5-8). The average prediabetes incidences for normal weight, normal weight combined overweight, and overweight/obesity were 47.4 (n = 3, range = 25.9-60.4), 46.3 (n = 2, range = 8-80.2), and 63.1 (n = 5, range = 10.0-95.9) per 1000 person-years, respectively.

### Publication bias

Funnel plots for the pooled incidence in underweight and overweight/obesity (Figure S5, panel A-C in the **Online Supplementary Document**) showed an asymmetrical distribution, and the Egger regression tests were insignificant (*P* = 0.59 for underweight and *P* = 0.42 for overweight/obesity), while the results for normal weight adults were significant (*P* = 0.04).

## DISCUSSION

We identified 94 prospective cohort studies containing data on the incidence of diabetes/prediabetes among approximately 3.4 million adults across varying weight statuses from 22 countries. This is the first meta-analysis to estimate the incidence of diabetes/prediabetes based on baseline weight status, which provides important insights for public health and clinical policies on diabetes prevention and management. The pooled incidences of diabetes in adults with underweight, normal weight, and overweight/obesity were 4.5, 2.7, and 10.5 per 1000 person-years, respectively. The incidence of prediabetes was higher than the incidence of diabetes, with averages of 47.4 in adults with normal weight and 63.1 per 1000 person-years in those with overweight/obesity. Moreover, this is the first study to assess the temporal trends in diabetes incidence by weight status. We found that diabetes incidence in adults with normal weight increased continuously from 1985 to 1990 with an estimated increase of 36% every five years. Diabetes incidence in adults with overweight/obesity sharply increased between 1995 and 2000 and then spiked 2010, while it greatly decreased in adults with underweight between 1995 and 2000 and between 2005 and 2010. Additionally, the pooled diabetes incidence varied by age, sex, country income, WHO region, study setting, weight assessment, and diabetes ascertainment methods.

Previous research has shown contradictory findings for trends of diabetes incidence. An aggregation analysis reported a stabilising or declining trend from 2010 onwards in many HICs [9], while the data from the Global Burden of Disease Study identified an increasing trend between 1990 and 2017 [128]. Although the two studies consistently used contemporary, real-world data over time to estimate the trends of diagnosed diabetes, they had no information on body weight. Our study is the first meta-analysis to assess the temporal trends of diabetes incidence by weight status, helping resolve this discrepancy. We found a significant increase in adults with overweight/obesity between 1995 and 2000 and after 2010. The temporal trends of diabetes in adults with underweight and overweight/obesity paralleled the trends of diabetes prevalence in those with underweight and overweight/obesity, respectively, reported by WHO during the same time period [129]. A shift in diet (traditional plant-based diet transitioned to more animal-based diet) caused by increasing economic status may be a potential explanation [130]. Animal-based diets provide sufficient nutrients to improve malnutrition status and excessive calories to increase obesity prevalence simultaneously [130]. Likewise, the temporal trends of diabetes incidence between 2000 and 2010 remained stable, as suggested by data from individual countries [11,131].

Importantly, we found that the temporal trend of diabetes incidence in adults with normal weight at baseline has increased robustly since 1985, with an estimated increase of 36% every five years, which contrasts stabilising trends of diabetes prevalence among adults with normal weight during recent decades [3-5]. This stabilising trend of diabetes prevalence may be caused by improved survival and declining mortality. Accumulating evidence has reported that the burden of diabetes among individuals with normal weight has increased, particularly in the Asian population [2,4,5]. Moreover, normal-weight individuals who develop diabetes may have a higher level of diabetes risk factors, including former smokers, hypertension, and physical inactivity [132]. Overweight/obesity has long been regarded as the critical parameter for diabetes control and management [16] and obesity is the primary focus in diabetes screening and intervention guidelines [12]. Our findings suggest that current diabetes screening policies and prevention strategies should not neglect non-overweight diabetes. More research to identify the metabolic differences between overweight and non-overweight diabetes, and the risk factors and effective prevention strategies for normal-weight diabetes is needed.

Moreover, our study confirmed that diabetes incidence in LMICs was significantly higher than that in HICs in adults with normal weight or overweight/obesity. This implies that the difference in diabetes prevalence between LMICs and HICs could be due to an increase in new diabetes cases in LMICs. However, the finding that LMICs had a lower diabetes incidence than HICs in underweight individuals is surprising. Underweight diabetes, as an atypical phenotype, may not fit into the classical definition of type 1 or type 2 diabetes well. However, its characteristics are commonly misdiagnosed as type 1 diabetes, particularly among lean individuals with deprived socioeconomic status in African and Asian countries [133]. The regional difference showed that the diabetes incidence in Americas and European regions was much lower than in the Western Pacific and South-East Asia. Apart from the economic level, ethnicity could also explain this regional difference [2]. The Asian population has a much lower BMI threshold for similar diabetes risk than the non-Asian population [22,134]. One of the strengths of our study is that we defined weight status using the ethnic-specific BMI classification [22]. These findings suggest that more efforts are needed to cope with the burden of diabetes in LMICs and non-Western countries.

Prediabetes is an intermediate metabolic state in the development of diabetes [135]. Evidence has shown that up to 70% of those with prediabetes will eventually progress to diabetes [12,135,136]. We found that the incidence of prediabetes was much higher than diabetes incidence even though the median follow-up period was shorter (four vs eight years). Importantly, the incidence of prediabetes was also high among adults with normal weight. Mapping the incidence of prediabetes is critical to predicting future trends of diabetes incidence. Currently, the data on prediabetes incidence stratified by weight status is limited, and further surveys are required.

### Limitations

Our study has several limitations. First, we filtered the search results to English-only studies and likely excluded those in other languages. However, we have tried to re-run title/abstract and full-text screening to include articles published in other languages; the newly identified articles did not affect the main results. Second, although assessing the temporal trends by using the median year of data collection is a widely used method [28-30], it fails to obtain an accurate yearly incidence of diabetes and estimate the secular trends of diabetes incidence. Third, there was substantial heterogeneity of the pooled diabetes/prediabetes incidences by weight status. We have conducted subgroup analyses and meta-regression to explore the source of this heterogeneity and identified that the WHO region and weight and diabetes assessment methods were considerable sources of heterogeneity. However, some other important factors that may play a critical role in the diabetes development but are difficult to extract were not considered, including ethnicity [137], family history [134], and dietary pattern [138]. Fourth, although we included diabetes ascertainment methods as a potential subgroup, diabetes determined by self-reporting and medical records cannot distinguish types 1 and 2 diabetes. However, the bias caused by type 1 diabetes might be small, because over 95% diabetic patients have type 2 diabetes [19]. Finally, we defined weight status only by BMI, without considering other anthropometric parameters, including waist circumstance, fat distribution, or visceral fat. Moreover, we categorised weight status by baseline BMI, without consideration of weight change during follow-up. Therefore, these findings should be interpreted cautiously, and more accurate and timely data are needed to assess the secular trends of diabetes incidence by weight status.

## CONCLUSIONS

We comprehensively assessed diabetes/prediabetes incidence across different weight statuses and their temporal trends. We found that the temporal trends of diabetes incidence for adults with overweight/obesity increased greatly, and while it showed a steady increase for those with normal weight, with an estimated increase of 36% every five years. More tailored prevention and intervention strategies and awareness campaigns to target non-overweight diabetes are required, particularly in LMICs and non-Western countries.

## Additional material


Online Supplementary Document

## References

[1] Lingvay I Sumithran P Cohen RV le Roux CW Obesity management as a primary treatment goal for type 2 diabetes: time to reframe the conversation. Lancet. 2022;399:394-405. 10.1016/S0140-6736(21)01919-X 34600604

[2] Gujral UP Narayan KMV Diabetes in Normal-Weight Individuals: High Susceptibility in Nonwhite Populations. Diabetes Care. 2019;42:2164-6. 10.2337/dci19-0046 31748211 PMC6868465

[3] Olaogun I Farag M Hamid P The pathophysiology of type 2 diabetes mellitus in non-obese individuals: an overview of the current understanding. Cureus. 2020;12:e7614. 10.7759/cureus.7614 32399348 PMC7213678

[4] Gujral UP Weber MB Staimez LR Narayan KMV Diabetes among non-overweight individuals: an emerging public health challenge. Curr Diab Rep. 2018;18:60. 10.1007/s11892-018-1017-1 29974263

[5] Vaag A Lund SS Non-obese patients with type 2 diabetes and prediabetic subjects: distinct phenotypes requiring special diabetes treatment and (or) prevention? Appl Physiol Nutr Metab. 2007;32:912-20. 10.1139/H07-100 18059616

[6] Menke A Casagrande S Geiss L Cowie CC Prevalence of and trends in diabetes among adults in the United States, 1988-2012. JAMA. 2015;314:1021-9. 10.1001/jama.2015.10029 26348752

[7] de Mestral C Stringhini S Guessous I Jornayvaz FR Thirteen-year trends in the prevalence of diabetes according to socioeconomic condition and cardiovascular risk factors in a Swiss population. BMJ Open Diabetes Res Care. 2020;8:e001273. 10.1136/bmjdrc-2020-001273 32661192 PMC7359178

[8] Shin JY Trends in the prevalence and management of diabetes in Korea: 2007-2017. Epidemiol Health. 2019;41:e2019029. 10.4178/epih.e2019029 31319658 PMC6702122

[9] Magliano DJ Chen L Islam RM Carstensen B Gregg EW Pavkov ME Trends in the incidence of diagnosed diabetes: a multicountry analysis of aggregate data from 22 million diagnoses in high-income and middle-income settings. Lancet Diabetes Endocrinol. 2021;9:203-11. 10.1016/S2213-8587(20)30402-2 33636102 PMC10984526

[10] Magliano DJ Islam RM Barr ELM Gregg EW Pavkov ME Harding JL Trends in incidence of total or type 2 diabetes: systematic review. BMJ. 2019;366:l5003. 10.1136/bmj.l5003 31511236 PMC6737490

[11] Abraham TM Pencina KM Pencina MJ Fox CS Trends in diabetes incidence: the Framingham Heart Study. Diabetes Care. 2015;38:482-7. 10.2337/dc14-1432 25552418 PMC4338506

[12] US Preventive Services Task Force Davidson KW Barry MJ Mangione CM Cabana M Caughey AB Screening for prediabetes and type 2 diabetes: US Preventive Services Task Force Recommendation Statement. JAMA. 2021;326:736-43. 10.1001/jama.2021.12531 34427594

[13] Li Y Teng D Shi X Qin G Qin Y Quan H Prevalence of diabetes recorded in mainland China using 2018 diagnostic criteria from the American Diabetes Association: national cross sectional study. BMJ. 2020;369:m997. 10.1136/bmj.m997 32345662 PMC7186854

[14] Page MJ McKenzie JE Bossuyt PM Boutron I Hoffmann TC Mulrow CD The PRISMA 2020 statement: an updated guideline for reporting systematic reviews. BMJ. 2021;372:n71. 10.1136/bmj.n71 33782057 PMC8005924

[15] Yu HJ Ho M Liu X Yang J Chau PH Fong DYT Association of weight status and the risks of diabetes in adults: a systematic review and meta-analysis of prospective cohort studies. Int J Obes (Lond). 2022;46:1101-13. 10.1038/s41366-022-01096-1 35197569

[16] Abdullah A Peeters A de Courten M Stoelwinder J The magnitude of association between overweight and obesity and the risk of diabetes: A meta-analysis of prospective cohort studies. Diabetes Res Clin Pract. 2010;89:309-19. 10.1016/j.diabres.2010.04.012 20493574

[17] Lotta LA Abbasi A Sharp SJ Sahlqvist AS Waterworth D Brosnan JM Definitions of metabolic health and risk of future type 2 diabetes in bmi categories: a systematic review and network meta-analysis. Diabetes Care. 2015;38:2177-87. 10.2337/dc15-1218 26494809 PMC4826609

[18] Aschner P New IDF clinical practice recommendations for managing type 2 diabetes in primary care. Diabetes Res Clin Pract. 2017;132:169-70. 10.1016/j.diabres.2017.09.002 28962686

[19] American Diabetes Association Professional Practice Committee 2. Classification and diagnosis of diabetes: standards of medical care in diabetes-2022. Diabetes Care. 2022;45:S17-38. 10.2337/dc22-S002 34964875

[20] Wells GA, Shea B, O’Connell D, Peterson J, Welch V, Losos M, et al. The Newcastle-Ottawa Scale (NOS) for assessing the quality of nonrandomised studies in meta-analyses. 2000. Available: http://www.ohri.ca/programs/clinical_epidemiology/oxford.asp. Accessed: 5 June 2023.

[21] Rudnicka AR Kapetanakis VV Jarrar Z Wathern AK Wormald R Fletcher AE Incidence of late-stage age-related macular degeneration in American Whites: systematic review and meta-analysis. Am J Ophthalmol. 2015;160:85-93.e3. 10.1016/j.ajo.2015.04.003 25857680

[22] WHO Expert Consultation Appropriate body-mass index for Asian populations and its implications for policy and intervention strategies. Lancet. 2004;363:157-63. 10.1016/S0140-6736(03)15268-3 14726171

[23] Liu XM Liu YJ Zhan J He QQ Overweight, obesity and risk of all-cause and cardiovascular mortality in patients with type 2 diabetes mellitus: a dose-response meta-analysis of prospective cohort studies. Eur J Epidemiol. 2015;30:35-45. 10.1007/s10654-014-9973-5 25421785

[24] Van Ganse E Kaufman L Derde MP Yernault JC Delaunois L Vincken W Effects of antihistamines in adult asthma: a meta-analysis of clinical trials. Eur Respir J. 1997;10:2216-24. 10.1183/09031936.97.10102216 9387943

[25] Fagerland MW. Evidence-based medicine and systematic reviews. In: Laake P, Benestad HB, Olsen BR, editors. Research in medical and biological sciences (Second Edition). Amsterdam: Academic Press; 2015. p. 431-61.

[26] The World Bank. World Bank Country and Lending Groups. Available: https://datahelpdesk.worldbank.org/knowledgebase/articles/906519-world-bank-country-and-lending-groups. Accessed: 10 August 2023.

[27] World Health OrganizationCountries. 2023. Available: https://www.who.int/countries. Accessed: 5 June 2023.

[28] Kunutsor SK Barrett MC Beswick AD Judge A Blom AW Wylde V Risk factors for dislocation after primary total hip replacement: a systematic review and meta-analysis of 125 studies involving approximately five million hip replacements. Lancet Rheumatol. 2019;1:e111-e121. 10.1016/S2665-9913(19)30045-1 38229338

[29] Berstock JR Beswick AD Lopez-Lopez JA Whitehouse MR Blom AW Mortality after total knee arthroplasty: a systematic review of incidence, temporal trends, and risk factors. J Bone Joint Surg Am. 2018;100:1064-70. 10.2106/JBJS.17.00249 29916935

[30] Kunutsor SK Barrett MC Whitehouse MR Craig RS Lenguerrand E Beswick AD Incidence, temporal trends and potential risk factors for prosthetic joint infection after primary total shoulder and elbow replacement: systematic review and meta-analysis. J Infect. 2020;80:426-36. 10.1016/j.jinf.2020.01.008 31981635

[31] Smolak A Chemaitelly H Hermez JG Low N Abu-Raddad LJ Epidemiology of Chlamydia trachomatis in the Middle East and north Africa: a systematic review, meta-analysis, and meta-regression. Lancet Glob Health. 2019;7:e1197-225. 10.1016/S2214-109X(19)30279-7 31402004

[32] Vatcheva KP Lee M McCormick JB Rahbar MH Multicollinearity in regression analyses conducted in epidemiologic studies. Epidemiology (Sunnyvale). 2016;6:227. 10.4172/2161-1165.1000227 27274911 PMC4888898

[33] Bourne RRA Flaxman SR Braithwaite T Cicinelli MV Das A Jonas JB Magnitude, temporal trends, and projections of the global prevalence of blindness and distance and near vision impairment: a systematic review and meta-analysis. Lancet Glob Health. 2017;5:e888-97. 10.1016/S2214-109X(17)30293-0 28779882

[34] Appleton SL Seaborn CJ Visvanathan R Hill CL Gill TK Taylor AW Diabetes and cardiovascular disease outcomes in the metabolically healthy obese phenotype: a cohort study. Diabetes Care. 2013;36:2388-94. 10.2337/dc12-1971 23491523 PMC3714523

[35] Arnlöv J Sundstrom J Ingelsson E Lind L Impact of BMI and the metabolic syndrome on the risk of diabetes in middle-aged men. Diabetes Care. 2011;34:61-5. 10.2337/dc10-0955 20852030 PMC3005442

[36] Asghar S Khan AK Ali SM Sayeed MA Bhowmik B Diep ML Incidence of diabetes in Asian-Indian subjects: a five year follow-up study from Bangladesh. Prim Care Diabetes. 2011;5:117-24. 10.1016/j.pcd.2011.01.002 21306967

[37] Åsvold BO Midthjell K Krokstad S Rangul V Bauman A Prolonged sitting may increase diabetes risk in physically inactive individuals: an 11 year follow-up of the HUNT Study, Norway. Diabetologia. 2017;60:830-5. 10.1007/s00125-016-4193-z 28054097

[38] Aung K Lorenzo C Hinojosa MA Haffner SM Risk of developing diabetes and cardiovascular disease in metabolically unhealthy normal-weight and metabolically healthy obese individuals. J Clin Endocrinol Metab. 2014;99:462-8. 10.1210/jc.2013-2832 24257907 PMC3913817

[39] Beleigoli AM Appleton SL Gill TK Hill CL Adams RJ Association of metabolic phenotypes, grip strength and diabetes risk: The 15-year follow-up of The North West Adelaide Health Study, Australia. Obes Res Clin Pract. 2020;14:536-41. 10.1016/j.orcp.2020.09.006 33041220

[40] Borné Y Nilsson PM Melander O Hedblad B Engström G Multiple anthropometric measures in relation to incidence of diabetes: a Swedish population-based cohort study. Eur J Public Health. 2015;25:1100-5. 10.1093/eurpub/ckv044 25817208

[41] Bragg F Tang K Guo Y Iona A Du H Holmes MV Associations of general and central adiposity with incident diabetes in Chinese men and women. Diabetes Care. 2018;41:494-502. 10.2337/dc17-1852 29298802 PMC6548563

[42] Carey VJ Walters EE Colditz GA Solomon CG Willett WC Rosner BA Body fat distribution and risk of non-insulin-dependent diabetes mellitus in women. The Nurses’ Health Study. Am J Epidemiol. 1997;145:614-9. 10.1093/oxfordjournals.aje.a009158 9098178

[43] Chan JCY Chee ML Tan NYQ Cheng CY Wong TY Sabanayagam C Differential effect of body mass index on the incidence of diabetes and diabetic retinopathy in two Asian populations. Nutr Diabetes. 2018;8:16. 10.1038/s41387-018-0018-0 29549238 PMC5856769

[44] Chan JM Rimm EB Colditz GA Stampfer MJ Willett WC Obesity, fat distribution, and weight gain as risk factors for clinical diabetes in men. Diabetes Care. 1994;17:961-9. 10.2337/diacare.17.9.961 7988316

[45] Chang Y Jung HS Yun KE Cho J Ahn J Chung EC Metabolically healthy obesity is associated with an increased risk of diabetes independently of nonalcoholic fatty liver disease. Obesity (Silver Spring). 2016;24:1996-2003. 10.1002/oby.21580 27474900

[46] de Mutsert R Sun Q Willett WC Hu FB van Dam RM Overweight in early adulthood, adult weight change, and risk of type 2 diabetes, cardiovascular diseases, and certain cancers in men: a cohort study. Am J Epidemiol. 2014;179:1353-65. 10.1093/aje/kwu052 24786797 PMC4036209

[47] Dotevall A Johansson S Wilhelmsen L Rosengren A Increased levels of triglycerides, BMI and blood pressure and low physical activity increase the risk of diabetes in Swedish women. A prospective 18-year follow-up of the BEDA study. Diabet Med. 2004;21:615-22. 10.1111/j.1464-5491.2004.01189.x 15154949

[48] Dow C Mangin M Balkau B Affret A Boutron-Ruault MC Clavel-Chapelon F Fatty acid consumption and incident type 2 diabetes: An 18-year follow-up in the female E3N (Etude Epidemiologique aupres des femmes de la Mutuelle Generale de l’Education Nationale) prospective cohort study. Br J Nutr. 2016;116:1807-15. 10.1017/S0007114516003883 27842617

[49] Dowse GK Incidence of NIDDM and the natural history of IGT in Pacific and Indian Ocean populations. Diabetes Res Clin Pract. 1996;34:S45-50. 10.1016/S0168-8227(96)90007-8 9015669

[50] Edwards MK Addoh O Sng E Ikuta T Carithers T Bertoni AG Physical activity, body mass index and waist circumference change, and normal-range glycated hemoglobin on incident diabetes: Jackson Heart Study. Postgrad Med. 2017;129:842-8. 10.1080/00325481.2017.1358065 28730854

[51] Feller S Boeing H Pischon T Body mass index, waist circumference, and the risk of type 2 diabetes mellitus: implications for routine clinical practice. Dtsch Arztebl Int. 2010;107:470-6. 20644701 10.3238/arztebl.2010.0470 PMC2905837

[52] Feng S Gong X Liu H Lu R Duan T Wang M The diabetes risk and determinants of transition from metabolically healthy to unhealthy phenotypes in 49,702 older adults: 4-year cohort study. Obesity (Silver Spring). 2020;28:1141-8. 10.1002/oby.22800 32374520

[53] Field AE Manson JE Laird N Williamson DF Willett WC Colditz GA Weight cycling and the risk of developing type 2 diabetes among adult women in the United States. Obes Res. 2004;12:267-74. 10.1038/oby.2004.34 14981219

[54] Ford ES Williamson DF Liu S Weight change and diabetes incidence: findings from a national cohort of US adults. Am J Epidemiol. 1997;146:214-22. 10.1093/oxfordjournals.aje.a009256 9247005

[55] Fox CS Pencina MJ Meigs JB Vasan RS Levitzky YS D’Agostino RB Sr Trends in the incidence of type 2 diabetes mellitus from the 1970s to the 1990s: the Framingham Heart Study. Circulation. 2006;113:2914-8. 10.1161/CIRCULATIONAHA.106.613828 16785337

[56] Fujita M Ueno K Hata A Effect of obesity on incidence of type 2 diabetes declines with age among Japanese women. Exp Biol Med (Maywood). 2009;234:750-7. 10.3181/0810-RM-292 19429850

[57] Gautier A Balkau B Lange C Tichet J Bonnet F Risk factors for incident type 2 diabetes in individuals with a BMI of <27 kg/m2: the role of gamma-glutamyltransferase. Data from an Epidemiological Study on the Insulin Resistance Syndrome (DESIR). Diabetologia. 2010;53:247-53. 10.1007/s00125-009-1602-6 19936701

[58] Giráldez-García C Franch-Nadal J Sangros FJ Ruiz A Carraminana F Goday A Adiposity and diabetes risk in adults with prediabetes: heterogeneity of findings depending on age and anthropometric measure. Obesity (Silver Spring). 2018;26:1481-90. 10.1002/oby.22256 30070055

[59] Hadaegh F Zabetian A Harati H Azizi F Waist/height ratio as a better predictor of type 2 diabetes compared to body mass index in Tehranian adult men–a 3.6-year prospective study. Exp Clin Endocrinol Diabetes. 2006;114:310-5. 10.1055/s-2006-924123 16868890

[60] Han C Liu Y Sun X Luo X Zhang L Wang B Prediction of a new body shape index and body adiposity estimator for development of type 2 diabetes mellitus: The Rural Chinese Cohort Study. Br J Nutr. 2017;118:771-6. 10.1017/S0007114517002859 29143718

[61] Hara H Egusa G Yamakido M Incidence of non-insulin-dependent diabetes mellitus and its risk factors in Japanese-Americans living in Hawaii and Los Angeles. Diabet Med. 1996;13:S133-42. 10.1002/dme.1996.13.s6.133 8894497

[62] Hayashi T Tsumura K Suematsu C Endo G Fujii S Okada K High normal blood pressure, hypertension, and the risk of type 2 diabetes in Japanese men. The Osaka Health Survey. Diabetes Care. 1999;22:1683-7. 10.2337/diacare.22.10.1683 10526735

[63] Hinnouho GM Czernichow S Dugravot A Nabi H Brunner EJ Kivimaki M Metabolically healthy obesity and the risk of cardiovascular disease and type 2 diabetes: the Whitehall II cohort study. Eur Heart J. 2015;36:551-9. 10.1093/eurheartj/ehu123 24670711 PMC4344958

[64] Holtermann A Gyntelberg F Bauman A Jensen MT Cardiorespiratory fitness, fatness and incident diabetes. Diabetes Res Clin Pract. 2017;134:113-20. 10.1016/j.diabres.2017.10.001 28993157

[65] Hu FB Manson JE Stampfer MJ Colditz G Liu S Solomon CG Diet, lifestyle, and the risk of type 2 diabetes mellitus in women. N Engl J Med. 2001;345:790-7. 10.1056/NEJMoa010492 11556298

[66] Hu G Jousilahti P Peltonen M Lindström J Tuomilehto J Urinary sodium and potassium excretion and the risk of type 2 diabetes: a prospective study in Finland. Diabetologia. 2005;48:1477-83. 10.1007/s00125-005-1824-1 15971060

[67] Hu G Lindström J Valle TT Eriksson JG Jousilahti P Silventoinen K Physical activity, body mass index, and risk of type 2 diabetes in patients with normal or impaired glucose regulation. Arch Intern Med. 2004;164:892-6. 10.1001/archinte.164.8.892 15111376

[68] Hu H Nagahama S Nanri A Tomita K Akter S Okazaki H Duration and degree of weight change and risk of incident diabetes: Japan Epidemiology Collaboration on Occupational Health Study. Prev Med. 2017;96:118-23. 10.1016/j.ypmed.2016.12.046 28040517

[69] Ishikawa-Takata K Ohta T Moritaki K Gotou T Inoue S Obesity, weight change and risks for hypertension, diabetes and hypercholesterolemia in Japanese men. Eur J Clin Nutr. 2002;56:601-7. 10.1038/sj.ejcn.1601364 12080398

[70] Jae SY Franklin BA Choo J Yoon ES Choi YH Park WH Fitness, body habitus, and the risk of incident type 2 diabetes mellitus in Korean men. Am J Cardiol. 2016;117:585-9. 10.1016/j.amjcard.2015.11.046 26721657

[71] Janghorbani M Salamat MR Amini M Aminorroaya A Risk of diabetes according to the metabolic health status and degree of obesity. Diabetes Metab Syndr. 2017;11 Suppl 1:S439-S444. 10.1016/j.dsx.2017.03.032 28404516

[72] Jiamjarasrangsi W Aekplakorn W Incidence and predictors of type 2 diabetes among professional and office workers in Bangkok, Thailand. J Med Assoc Thai. 2005;88:1896-904. 16518992

[73] Jung CH Lee MJ Kang YM Jang JE Leem J Hwang JY The risk of incident type 2 diabetes in a Korean metabolically healthy obese population: the role of systemic inflammation. J Clin Endocrinol Metab. 2015;100:934-41. 10.1210/jc.2014-3885 25490279

[74] Jung HH Park JI Jeong JS Incidence of diabetes and its mortality according to body mass index in south Koreans aged 40-79 years. Clin Epidemiol. 2017;9:667-78. 10.2147/CLEP.S146860 29263705 PMC5724411

[75] Jung JY Park SK Oh CM Ryoo JH Choi JM Choi YJ The risk of type 2 diabetes mellitus according to the categories of body mass index: the Korean Genome and Epidemiology Study (KoGES). Acta Diabetol. 2018;55:479-84. 10.1007/s00592-018-1112-4 29455425

[76] Krishnan S Rosenberg L Djoussé L Cupples LA Palmer JR Overall and central obesity and risk of type 2 diabetes in U.S. black women. Obesity (Silver Spring). 2007;15:1860-6. 10.1038/oby.2007.220 17636105

[77] Li F Duan J Yang Y Yan G Chen Z Wang J Distinct uric acid trajectories are associated with incident diabetes in an overweight Chinese population. Diabetes Metab. 2021;47:101175. 10.1016/j.diabet.2020.07.002 32730902

[78] Li WD Fu KF Li GM Lian YS Ren AM Chen YJ Comparison of effects of obesity and non-alcoholic fatty liver disease on incidence of type 2 diabetes mellitus. World J Gastroenterol. 2015;21:9607-13. 10.3748/wjg.v21.i32.9607 26327768 PMC4548121

[79] Lim JS Lee DH Park JY Jin SH Jacobs DR A strong interaction between serum gamma-glutamyltransferase and obesity on the risk of prevalent type 2 diabetes: Results from the Third National Health and Nutrition Examination Survey. Clin Chem. 2007;53:1092-8. 10.1373/clinchem.2006.079814 17478563

[80] Manson JE Rimm EB Stampfer MJ Colditz GA Willett WC Krolewski AS Physical activity and incidence of non-insulin-dependent diabetes mellitus in women. Lancet. 1991;338:774-8. 10.1016/0140-6736(91)90664-B 1681160

[81] Maty SC Everson-Rose SA Haan MN Raghunathan TE Kaplan GA Education, income, occupation, and the 34-year incidence (1965-99) of Type 2 diabetes in the Alameda County Study. Int J Epidemiol. 2005;34:1274-81. 10.1093/ije/dyi167 16120636 PMC3172611

[82] Meigs JB Wilson PW Fox CS Vasan RS Nathan DM Sullivan LM Body mass index, metabolic syndrome, and risk of type 2 diabetes or cardiovascular disease. J Clin Endocrinol Metab. 2006;91:2906-12. 10.1210/jc.2006-0594 16735483

[83] Meisinger C Doring A Thorand B Heier M Lowel H Body fat distribution and risk of type 2 diabetes in the general population: are there differences between men and women? The MONICA/KORA Augsburg cohort study. Am J Clin Nutr. 2006;84:483-9. 10.1093/ajcn/84.3.483 16960160

[84] Mishra GD Carrigan G Brown WJ Barnett AG Dobson AJ Short-term weight change and the incidence of diabetes in midlife: results from the Australian Longitudinal Study on Women’s Health. Diabetes Care. 2007;30:1418-24. 10.2337/dc06-2187 17351279

[85] Nagayoshi M Punjabi NM Selvin E Pankow JS Shahar E Iso H Obstructive sleep apnea and incident type 2 diabetes. Sleep Med. 2016;25:156-61. 10.1016/j.sleep.2016.05.009 27810258 PMC5102826

[86] Nanri A Mizoue T Takahashi Y Kirii K Inoue M Noda M Soy product and isoflavone intakes are associated with a lower risk of type 2 diabetes in overweight Japanese women. J Nutr. 2010;140:580-6. 10.3945/jn.109.116020 20053935

[87] Nguyen B Bauman A Ding D Incident type 2 diabetes in a large Australian cohort study: does physical activity or sitting time alter the risk associated with body mass index? J Phys Act Health. 2017;14:13-9. 10.1123/jpah.2016-0184 27618727

[88] Ning F Zhang D Xue B Zhang L Zhang J Zhu Z Synergistic effects of depression and obesity on type 2 diabetes incidence in Chinese adults. J Diabetes. 2020;12:142-50. 10.1111/1753-0407.12968 31287240

[89] Oguma Y Sesso HD Paffenbarger RS Jr Lee IM Weight change and risk of developing type 2 diabetes. Obes Res. 2005;13:945-51. 10.1038/oby.2005.109 15919849

[90] Ohlsson C Bygdell M Nethander M Rosengren A Kindblom JM BMI change during puberty is an important determinant of adult type 2 diabetes risk in men. J Clin Endocrinol Metab. 2019;104:1823-32. 10.1210/jc.2018-01339 30517677 PMC6456008

[91] Papier K D’Este C Bain C Banwell C Seubsman SA Sleigh A Body mass index and type 2 diabetes in Thai adults: defining risk thresholds and population impacts. BMC Public Health. 2017;17:707. 10.1186/s12889-017-4708-7 28915801 PMC5602842

[92] Sairenchi T Iso H Irie F Fukasawa N Ota H Muto T Underweight as a predictor of diabetes in older adults: a large cohort study. Diabetes Care. 2008;31:583-4. 10.2337/dc07-1390 18071003

[93] Sasai H Sairenchi T Iso H Irie F Otaka E Tanaka K Relationship between obesity and incident diabetes in middle-aged and older Japanese adults: the Ibaraki Prefectural Health Study. Mayo Clin Proc. 2010;85:36-40. 10.4065/mcp.2009.0230 20042559 PMC2800296

[94] Schmidt M Johannesdottir SA Lemeshow S Lash TL Ulrichsen SP Botker HE Obesity in young men, and individual and combined risks of type 2 diabetes, cardiovascular morbidity and death before 55 years of age: a Danish 33-year follow-up study. BMJ Open. 2013;3:e002698. 10.1136/bmjopen-2013-002698 23628994 PMC3641453

[95] Sheikh MA Lund E Braaten T The predictive effect of body mass index on type 2 diabetes in the Norwegian women and cancer study. Lipids Health Dis. 2014;13:164. 10.1186/1476-511X-13-164 25344292 PMC4223755

[96] Siegel LC Sesso HD Bowman TS Lee IM Manson JE Gaziano JM Physical activity, body mass index, and diabetes risk in men: a prospective study. Am J Med. 2009;122:1115-21. 10.1016/j.amjmed.2009.02.008 19958889 PMC2789347

[97] Song BM Kim HC Kim DJ Ahn SV Kim KM Lee JM Aminotransferase levels, body mass index, and the risk of diabetes: a prospective cohort study. Ann Epidemiol. 2018;28:675-80.e6. 10.1016/j.annepidem.2018.07.009 30075987

[98] Sui X Hooker SP Lee IM Church TS Colabianchi N Lee CD A prospective study of cardiorespiratory fitness and risk of type 2 diabetes in women. Diabetes Care. 2008;31:550-5. 10.2337/dc07-1870 18070999 PMC3410433

[99] Sun J Bao G Cui J Yasmeen N Aslam B Xin H The association of diabetes risk score and body mass index with incidence of diabetes among urban and rural adult communities in Qingdao, China. Int J Diabetes Dev Ctries. 2019;39:730-8. 10.1007/s13410-019-00740-3

[100] Tatsumi Y Ohno Y Morimoto A Nishigaki Y Maejima F Mizuno S U-shaped relationship between body mass index and incidence of diabetes. Diabetol Int. 2012;3:92-8. 10.1007/s13340-012-0067-x

[101] Twig G Afek A Derazne E Tzur D Cukierman-Yaffe T Gerstein HC Diabetes risk among overweight and obese metabolically healthy young adults. Diabetes Care. 2014;37:2989-95. 10.2337/dc14-0869 25139886

[102] Uemura M Yatsuya H Hilawe EH Li Y Wang C Chiang C Breakfast Skipping is Positively Associated With Incidence of Type 2 Diabetes Mellitus: Evidence From the Aichi Workers’ Cohort Study. J Epidemiol. 2015;25:351-8. 10.2188/jea.JE20140109 25787236 PMC4411234

[103] Vaidya A Cui L Sun L Lu B Chen S Liu X A prospective study of impaired fasting glucose and type 2 diabetes in China: The Kailuan study. Medicine (Baltimore). 2016;95:e5350. 10.1097/MD.0000000000005350 27861364 PMC5120921

[104] Villegas R Shu XO Yang G Matthews CE Li H Cai H Energy balance and type 2 diabetes: a report from the Shanghai Women’s Health Study. Nutr Metab Cardiovasc Dis. 2009;19:190-7. 10.1016/j.numecd.2008.06.003 18774701 PMC2701731

[105] Wang B Zhang M Wang S Wang C Wang J Li L Dynamic status of metabolically healthy overweight/obesity and metabolically unhealthy and normal weight and the risk of type 2 diabetes mellitus: A cohort study of a rural adult Chinese population. Obes Res Clin Pract. 2018;12:61-71. 10.1016/j.orcp.2017.10.005 29100915

[106] Wang H Shara NM Calhoun D Umans JG Lee ET Howard BV Incidence rates and predictors of diabetes in those with prediabetes: the Strong Heart Study. Diabetes Metab Res Rev. 2010;26:378-85. 10.1002/dmrr.1089 20578203 PMC2897954

[107] Wang Y Rimm EB Stampfer MJ Willett WC Hu FB Comparison of abdominal adiposity and overall obesity in predicting risk of type 2 diabetes among men. Am J Clin Nutr. 2005;81:555-63. 10.1093/ajcn/81.3.555 15755822

[108] Wannamethee SG Shaper AG Walker M Overweight and obesity and weight change in middle aged men: impact on cardiovascular disease and diabetes. J Epidemiol Community Health. 2005;59:134-9. 10.1136/jech.2003.015651 15650145 PMC1733005

[109] Wei Y Wang J Han X Yu C Wang F Yuan J Metabolically healthy obesity increased diabetes incidence in a middle-aged and elderly Chinese population. Diabetes Metab Res Rev. 2020;36:e3202. 10.1002/dmrr.3202 31291052

[110] Weinstein AR Sesso HD Lee IM Cook NR Manson JE Buring JE Relationship of physical activity vs body mass index with type 2 diabetes in women. JAMA. 2004;292:1188-94. 10.1001/jama.292.10.1188 15353531

[111] Will JC Williamson DF Ford ES Calle EE Thun MJ Intentional weight loss and 13-year diabetes incidence in overweight adults. Am J Public Health. 2002;92:1245-8. 10.2105/AJPH.92.8.1245 12144977 PMC1447223

[112] Williams PT Hoffman K La I Weight-related increases in hypertension, hypercholesterolemia, and diabetes risk in normal weight male and female runners. Arterioscler Thromb Vasc Biol. 2007;27:1811-9. 10.1161/ATVBAHA.107.141853 17510467

[113] Xia MF Lin HD Chen LY Wu L Ma H Li Q Association of visceral adiposity and its longitudinal increase with the risk of diabetes in Chinese adults: A prospective cohort study. Diabetes Metab Res Rev. 2018;34:e3048. 10.1002/dmrr.3048 30035847

[114] Ye M Robson PJ Eurich DT Vena JE Xu JY Johnson JA Changes in body mass index and incidence of diabetes: A longitudinal study of Alberta’s Tomorrow Project Cohort. Prev Med. 2018;106:157-63. 10.1016/j.ypmed.2017.10.036 29117506

[115] Lee DH Keum N Hu FB Orav EJ Rimm EB Willett WC Comparison of the association of predicted fat mass, body mass index, and other obesity indicators with type 2 diabetes risk: two large prospective studies in US men and women. Eur J Epidemiol. 2018;33:1113-23. 10.1007/s10654-018-0433-5 30117031

[116] Sakurai M Ishizaki M Morikawa Y Kido T Naruse Y Nakashima Y Frequency of consumption of balanced meals, bodyweight gain and incident risk of glucose intolerance in Japanese men and women: A cohort study. J Diabetes Investig. 2021;12:763-70. 10.1111/jdi.13392 32869545 PMC8089009

[117] Wang G Radovick S Xu X Xing H Tang G Bartell TR Strategy for early identification of prediabetes in lean populations: New insight from a prospective Chinese twin cohort of children and young adults. Diabetes Res Clin Pract. 2018;146:101-10. 10.1016/j.diabres.2018.10.003 30312713

[118] André P Proctor G Driollet B Garcia-Esquinas E Lopez-Garcia E Gomez-Cabrero D The role of overweight in the association between the Mediterranean diet and the risk of type 2 diabetes mellitus: a mediation analysis among 21 585 UK biobank participants. Int J Epidemiol. 2020;49:1582-90. 10.1093/ije/dyaa103 32754745

[119] Bardenheier BH Wu WC Zullo AR Gravenstein S Gregg EW Progression to diabetes by baseline glycemic status among middle-aged and older adults in the United States, 2006-2014. Diabetes Res Clin Pract. 2021;174:108726. 10.1016/j.diabres.2021.108726 33662490

[120] Chen Y Wang N Dong X Zhu J Chen Y Jiang Q Associations between serum amino acids and incident type 2 diabetes in Chinese rural adults. Nutr Metab Cardiovasc Dis. 2021;31:2416-25. 10.1016/j.numecd.2021.05.004 34158241

[121] Cuthbertson DJ Koskinen J Brown E Magnussen CG Hutri-Kahonen N Sabin M Fatty liver index predicts incident risk of prediabetes, type 2 diabetes and non-alcoholic fatty liver disease (NAFLD). Ann Med. 2021;53:1256-64. 10.1080/07853890.2021.1956685 34309471 PMC8317942

[122] Hodge AM Karim MN Hebert JR Shivappa N de Courten B Association between diet quality indices and incidence of type 2 diabetes in the Melbourne Collaborative Cohort Study. Nutrients. 2021;13:4162. 10.3390/nu13114162 34836416 PMC8622769

[123] Narayan KMV Kondal D Kobes S Staimez LR Mohan D Gujral UP Incidence of diabetes in South Asian young adults compared to Pima Indians. BMJ Open Diabetes Res Care. 2021;9:e001988. 10.1136/bmjdrc-2020-001988 33771765 PMC8006824

[124] Tang ML Zhou YQ Song AQ Wang JL Wan YP Xu RY The relationship between body mass index and incident diabetes mellitus in Chinese aged population: a cohort study. J Diabetes Res. 2021;2021:5581349. 10.1155/2021/5581349 34485532 PMC8410436

[125] Xi Y Gao W Zheng K Lv J Yu C Wang S Overweight and risk of type 2 diabetes: a prospective Chinese twin study. Diabetes Metab. 2022;48:101278. 10.1016/j.diabet.2021.101278 34520837

[126] Xu S Ming J Jia A Yu X Cai J Jing C Normal weight obesity and the risk of diabetes in Chinese people: a 9-year population-based cohort study. Sci Rep. 2021;11:6090. 10.1038/s41598-021-85573-z 33731778 PMC7969601

[127] Zhu X Hu J Guo H Ji D Yuan D Li M Effect of metabolic health and obesity phenotype on risk of diabetes mellitus: A population-based longitudinal study. Diabetes Metab Syndr Obes. 2021;14:3485-98. 10.2147/DMSO.S317739 34385823 PMC8353171

[128] Liu J Ren ZH Qiang H Wu J Shen M Zhang L Trends in the incidence of diabetes mellitus: results from the Global Burden of Disease Study 2017 and implications for diabetes mellitus prevention. BMC Public Health. 2020;20:1415. 10.1186/s12889-020-09502-x 32943028 PMC7500018

[129] George AM Jacob AG Fogelfeld L Lean diabetes mellitus: An emerging entity in the era of obesity. World J Diabetes. 2015;6:613-20. 10.4239/wjd.v6.i4.613 25987958 PMC4434081

[130] NCD Risk Factor Collaboration (NCD-RisC) Worldwide trends in body-mass index, underweight, overweight, and obesity from 1975 to 2016: a pooled analysis of 2416 population-based measurement studies in 128.9 million children, adolescents, and adults. Lancet. 2017;390:2627-42. 10.1016/S0140-6736(17)32129-3 29029897 PMC5735219

[131] Read SH Kerssens JJ McAllister DA Colhoun HM Fischbacher CM Lindsay RS Trends in type 2 diabetes incidence and mortality in Scotland between 2004 and 2013. Diabetologia. 2016;59:2492. 10.1007/s00125-016-4089-y 27597171 PMC6828276

[132] Eckel N Muhlenbruch K Meidtner K Boeing H Stefan N Schulze MB Characterization of metabolically unhealthy normal-weight individuals: risk factors and their associations with type 2 diabetes. Metabolism. 2015;64:862-71. 10.1016/j.metabol.2015.03.009 25861921

[133] Bavuma C Sahabandu D Musafiri S Danquah I McQuillan R Wild S Atypical forms of diabetes mellitus in Africans and other non-European ethnic populations in low- and middle-income countries: a systematic literature review. J Glob Health. 2019;9:020401. 10.7189/jogh.09.020401 31673335 PMC6818125

[134] InterAct Consortium Scott RA Langenberg C Sharp SJ Franks PW Rolandsson O The link between family history and risk of type 2 diabetes is not explained by anthropometric, lifestyle or genetic risk factors: the EPIC-InterAct study. Diabetologia. 2013;56:60-9. 10.1007/s00125-012-2715-x 23052052 PMC4038917

[135] Tabák AG Herder C Rathmann W Brunner EJ Kivimaki M Prediabetes: a high-risk state for diabetes development. Lancet. 2012;379:2279-90. 10.1016/S0140-6736(12)60283-9 22683128 PMC3891203

[136] Li G Zhang P Wang J Gregg EW Yang W Gong Q The long-term effect of lifestyle interventions to prevent diabetes in the China Da Qing Diabetes Prevention Study: a 20-year follow-up study. Lancet. 2008;371:1783-9. 10.1016/S0140-6736(08)60766-7 18502303

[137] Spanakis EK Golden SH Race/ethnic difference in diabetes and diabetic complications. Curr Diab Rep. 2013;13:814-23. 10.1007/s11892-013-0421-9 24037313 PMC3830901

[138] Neuenschwander M Ballon A Weber KS Norat T Aune D Schwingshackl L Role of diet in type 2 diabetes incidence: umbrella review of meta-analyses of prospective observational studies. BMJ. 2019;366:l2368. 10.1136/bmj.l2368 31270064 PMC6607211

