# Supplementary material for: Incidence and temporal trends in type 2 diabetes by weight status: A systematic review and meta-analysis of prospective cohort studies
Source: J Glob Health. 2023 Sep 1;13:04088. doi: 10.7189/jogh.13.04088 (PMC10471153; doi:10.7189/jogh.13.04088)

Online supplementary document

Contents

Supplementary Table 1 Search strategy (PubMed as an example) 1

Supplementary Table 2 Study quality of included studies assessed by NOS 2

Supplementary Table 3 The characteristics of included studies reported diabetes incidence 4

Supplementary Table 4 Leave-one-out analysis for the pooled incidence of diabetes by baseline weight status 19

Supplementary Table 5 The correlation matrix of modifiers that included for multivariate meta-regression models by baseline weight status 23

Supplementary Table 6 The characteristics of included studies reported prediabetes incidence 24

Supplementary Figures legends and Figures 26

The included studies

1. Appleton SL, Seaborn CJ, Visvanathan R, et al. Diabetes and cardiovascular disease outcomes in the metabolically healthy obese phenotype: A cohort study. *Diabetes Care* 2013; 36(8): 2388-94.
2. Arnlov J, Sundstrom J, Ingelsson E, Lind L. Impact of bmi and the metabolic syndrome on the risk of diabetes in middle-aged men. *Diabetes Care* 2011; 34(1): 61-5.
3. Asghar S, Khan AK, Ali SM, et al. Incidence of diabetes in asian-indian subjects: A five year follow-up study from bangladesh. *Prim Care Diabetes* 2011; 5(2): 117-24.
4. Asvold BO, Midthjell K, Krokstad S, Rangul V, Bauman A. Prolonged sitting may increase diabetes risk in physically inactive individuals: An 11 year follow-up of the hunt study, norway. *Diabetologia* 2017; 60(5): 830-5.
5. Aung K, Lorenzo C, Hinojosa MA, Haffner SM. Risk of developing diabetes and cardiovascular disease in metabolically unhealthy normal-weight and metabolically healthy obese individuals. *J Clin Endocrinol Metab* 2014; 99(2): 462-8.
6. Beleigoli AM, Appleton SL, Gill TK, Hill CL, Adams RJ. Association of metabolic phenotypes, grip strength and diabetes risk: The 15-year follow-up of the north west adelaide health study, australia. *Obes Res Clin Pract* 2020; 14(6): 536-41.
7. Borné Y, Nilsson PM, Melander O, Hedblad B, Engström G. Multiple anthropometric measures in relation to incidence of diabetes: A swedish population-based cohort study. *Eur J Public Health* 2015; 25(6): 1100-5.
8. Bragg F, Tang K, Guo Y, et al. Associations of general and central adiposity with incident diabetes in chinese men and women. *Diabetes Care* 2018; 41(3): 494-502.
9. Carey VJ, Walters EE, Colditz GA, et al. Body fat distribution and risk of non-insulin-dependent diabetes mellitus in women. The nurses' health study. *Am J Epidemiol* 1997; 145(7): 614-9.
10. Chan JCY, Chee ML, Tan NYQ, Cheng CY, Wong TY, Sabanayagam C. Differential effect of body mass index on the incidence of diabetes and diabetic retinopathy in two asian populations. *Nutr Diabetes* 2018; 8(1): 16.
11. Chan JM, Rimm EB, Colditz GA, Stampfer MJ, Willett WC. Obesity, fat distribution, and weight gain as risk factors for clinical diabetes in men. *Diabetes Care* 1994; 17(9): 961-9.
12. Chang Y, Jung HS, Yun KE, et al. Metabolically healthy obesity is associated with an increased risk of diabetes independently of nonalcoholic fatty liver disease. *Obesity (Silver Spring)* 2016; 24(9): 1996-2003.
13. de Mutsert R, Sun Q, Willett WC, Hu FB, van Dam RM. Overweight in early adulthood, adult weight change, and risk of type 2 diabetes, cardiovascular diseases, and certain cancers in men: A cohort study. *Am J Epidemiol* 2014; 179(11): 1353-65.
14. Dotevall A, Johansson S, Wilhelmsen L, Rosengren A. Increased levels of triglycerides, bmi and blood pressure and low physical activity increase the risk of diabetes in swedish women. A prospective 18-year follow-up of the beda study. *Diabet Med* 2004; 21(6): 615-22.
15. Dow C, Mangin M, Balkau B, et al. Fatty acid consumption and incident type 2 diabetes: An 18-year follow-up in the female e3n (etude epidemiologique aupres des femmes de la mutuelle generale de l'education nationale) prospective cohort study. *British Journal of Nutrition* 2016; 116(10): 1807-15.
16. Dowse GK. Incidence of niddm and the natural history of igt in pacific and indian ocean populations. *Diabetes Research and Clinical Practice* 1996; 34: S45-S50.
17. Edwards MK, Addoh O, Sng E, et al. Physical activity, body mass index and waist circumference change, and normal-range glycated hemoglobin on incident diabetes: Jackson heart study. *Postgraduate Medicine* 2017; 129(8): 842-8.
18. Feller S, Boeing H, Pischon T. Body mass index, waist circumference, and the risk of type 2 diabetes mellitus: Implications for routine clinical practice. *Dtsch Arztebl Int* 2010; 107(26): 470-6.
19. Feng S, Gong X, Liu H, et al. The diabetes risk and determinants of transition from metabolically healthy to unhealthy phenotypes in 49,702 older adults: 4-year cohort study. *Obesity (Silver Spring)* 2020; 28(6): 1141-8.
20. Field AE, Manson JE, Laird N, Williamson DF, Willett WC, Colditz GA. Weight cycling and the risk of developing type 2 diabetes among adult women in the united states. *Obes Res* 2004; 12(2): 267-74.
21. Ford ES, Williamson DF, Liu S. Weight change and diabetes incidence: Findings from a national cohort of us adults. *Am J Epidemiol* 1997; 146(3): 214-22.
22. Fox CS, Pencina MJ, Meigs JB, Vasan RS, Levitzky YS, D'Agostino RB, Sr. Trends in the incidence of type 2 diabetes mellitus from the 1970s to the 1990s: The framingham heart study. *Circulation* 2006; 113(25): 2914-8.
23. Fujita M, Ueno K, Hata A. Effect of obesity on incidence of type 2 diabetes declines with age among japanese women. *Exp Biol Med (Maywood)* 2009; 234(7): 750-7.

24. Gautier A, Balkau B, Lange C, Tichet J, Bonnet F. Risk factors for incident type 2 diabetes in individuals with a bmi of <27 kg/m2: The role of gamma-glutamyltransferase. Data from an epidemiological study on the insulin resistance syndrome (desir). *Diabetologia* 2010; 53(2): 247-53.
25. Giraldez-Garcia C, Franch-Nadal J, Sangros FJ, et al. Adiposity and diabetes risk in adults with prediabetes: Heterogeneity of findings depending on age and anthropometric measure. *Obesity* 2018; 26(9): 1481-90.
26. Hadaegh F, Zabetian A, Harati H, Azizi F. Waist/height ratio as a better predictor of type 2 diabetes compared to body mass index in tehranian adult men--a 3.6-year prospective study. *Exp Clin Endocrinol Diabetes* 2006; 114(6): 310-5.
27. Han C, Liu Y, Sun X, et al. Prediction of a new body shape index and body adiposity estimator for development of type 2 diabetes mellitus: The rural chinese cohort study. *Br J Nutr* 2017; 118(10): 771-6.
28. Hara H, Egusa G, Yamakido M. Incidence of non-insulin-dependent diabetes mellitus and its risk factors in japanese-americans living in hawaii and los angeles. *Diabet Med* 1996; 13(9 Suppl 6): S133-42.
29. Hayashi T, Tsumura K, Suematsu C, Endo G, Fujii S, Okada K. High normal blood pressure, hypertension, and the risk of type 2 diabetes in japanese men. The osaka health survey. *Diabetes Care* 1999; 22(10): 1683-7.
30. Hinnouho GM, Czernichow S, Dugravot A, et al. Metabolically healthy obesity and the risk of cardiovascular disease and type 2 diabetes: The whitehall ii cohort study. *Eur Heart J* 2015; 36(9): 551-9.
31. Holtermann A, Gyntelberg F, Bauman A, Jensen MT. Cardiorespiratory fitness, fatness and incident diabetes. *Diabetes Res Clin Pract* 2017; 134: 113-20.
32. Hu FB, Manson JE, Stampfer MJ, et al. Diet, lifestyle, and the risk of type 2 diabetes mellitus in women. *N Engl J Med* 2001; 345(11): 790-7.
33. Hu G, Jousilahti P, Peltonen M, Lindström J, Tuomilehto J. Urinary sodium and potassium excretion and the risk of type 2 diabetes: A prospective study in finland. *Diabetologia* 2005; 48(8): 1477-83.
34. Hu G, Lindström J, Valle TT, et al. Physical activity, body mass index, and risk of type 2 diabetes in patients with normal or impaired glucose regulation. *Arch Intern Med* 2004; 164(8): 892-6.
35. Hu H, Nagahama S, Nanri A, et al. Duration and degree of weight change and risk of incident diabetes: Japan epidemiology collaboration on occupational health study. *Prev Med* 2017; 96: 118-23.
36. Ishikawa-Takata K, Ohta T, Moritaki K, Gotou T, Inoue S. Obesity, weight change and risks for hypertension, diabetes and hypercholesterolemia in japanese men. *Eur J Clin Nutr* 2002; 56(7): 601-7.
37. Jae SY, Franklin BA, Choo J, Yoon ES, Choi YH, Park WH. Fitness, body habitus, and the risk of incident type 2 diabetes mellitus in korean men. *Am J Cardiol* 2016; 117(4): 585-9.
38. Janghorbani M, Salamat MR, Amini M, Aminorroaya A. Risk of diabetes according to the metabolic health status and degree of obesity. *Diabetes Metab Syndr* 2017; 11 Suppl 1: S439-s44.
39. Jiamjarasrangsi W, Aekplakorn W. Incidence and predictors of type 2 diabetes among professional and office workers in bangkok, thailand. *J Med Assoc Thai* 2005; 88(12): 1896-904.
40. Jung CH, Lee MJ, Kang YM, et al. The risk of incident type 2 diabetes in a korean metabolically healthy obese population: The role of systemic inflammation. *J Clin Endocrinol Metab* 2015; 100(3): 934-41.
41. Jung HH, Park JI, Jeong JS. Incidence of diabetes and its mortality according to body mass index in south koreans aged 40-79 years. *Clin Epidemiol* 2017; 9: 667-78.
42. Jung JY, Park SK, Oh CM, Ryoo JH, Choi JM, Choi YJ. The risk of type 2 diabetes mellitus according to the categories of body mass index: The korean genome and epidemiology study (koges). *Acta Diabetol* 2018; 55(5): 479-84.
43. Krishnan S, Rosenberg L, Djoussé L, Cupples LA, Palmer JR. Overall and central obesity and risk of type 2 diabetes in u.S. Black women. *Obesity (Silver Spring)* 2007; 15(7): 1860-6.
44. Lee DH, Keum N, Hu FB, et al. Comparison of the association of predicted fat mass, body mass index, and other obesity indicators with type 2 diabetes risk: Two large prospective studies in us men and women. *Eur J Epidemiol* 2018; 33(11): 1113-23.
45. Li F, Duan J, Yang Y, et al. Distinct uric acid trajectories are associated with incident diabetes in an overweight chinese population. *Diabetes and Metabolism* 2020.
46. Li WD, Fu KF, Li GM, et al. Comparison of effects of obesity and non-alcoholic fatty liver disease on incidence of type 2 diabetes mellitus. *World J Gastroenterol* 2015; 21(32): 9607-13.
47. Lim JS, Lee DH, Park JY, Jin SH, Jacobs DR. A strong interaction between serum gamma-glutamyltransferase and obesity on the risk of prevalent type 2 diabetes: Results from the third national health and nutrition examination survey. *Clinical Chemistry* 2007; 53(6): 1092-8.

48. Manson JE, Rimm EB, Stampfer MJ, et al. Physical activity and incidence of non-insulin-dependent diabetes mellitus in women. *Lancet* 1991; 338(8770): 774-8.
49. Maty SC, Everson-Rose SA, Haan MN, Raghunathan TE, Kaplan GA. Education, income, occupation, and the 34-year incidence (1965-99) of type 2 diabetes in the alameda county study. *Int J Epidemiol* 2005; 34(6): 1274-81.
50. Meigs JB, Wilson PW, Fox CS, et al. Body mass index, metabolic syndrome, and risk of type 2 diabetes or cardiovascular disease. *J Clin Endocrinol Metab* 2006; 91(8): 2906-12.
51. Meisinger C, Doring A, Thorand B, Heier M, Lowel H. Body fat distribution and risk of type 2 diabetes in the general population: Are there differences between men and women? The monica/kora augsburg cohort study. *Am J Clin Nutr* 2006; 84(3): 483-9.
52. Mishra GD, Carrigan G, Brown WJ, Barnett AG, Dobson AJ. Short-term weight change and the incidence of diabetes in midlife: Results from the australian longitudinal study on women's health. *Diabetes Care* 2007; 30(6): 1418-24.
53. Nagayoshi M, Punjabi NM, Selvin E, et al. Obstructive sleep apnea and incident type 2 diabetes. *Sleep Med* 2016; 25: 156-61.
54. Nanri A, Mizoue T, Takahashi Y, et al. Soy product and isoflavone intakes are associated with a lower risk of type 2 diabetes in overweight japanese women. *J Nutr* 2010; 140(3): 580-6.
55. Nguyen B, Bauman A, Ding D. Incident type 2 diabetes in a large australian cohort study: Does physical activity or sitting time alter the risk associated with body mass index? *J Phys Act Health* 2017; 14(1): 13-9.
56. Ning F, Zhang D, Xue B, et al. Synergistic effects of depression and obesity on type 2 diabetes incidence in chinese adults. *J Diabetes* 2020; 12(2): 142-50.
57. Oguma Y, Sesso HD, Paffenbarger RS, Jr., Lee IM. Weight change and risk of developing type 2 diabetes. *Obes Res* 2005; 13(5): 945-51.
58. Ohlsson C, Bygdell M, Nethander M, Rosengren A, Kindblom JM. Bmi change during puberty is an important determinant of adult type 2 diabetes risk in men. *J Clin Endocrinol Metab* 2019; 104(5): 1823-32.
59. Papier K, D'Este C, Bain C, et al. Body mass index and type 2 diabetes in thai adults: Defining risk thresholds and population impacts. *BMC Public Health* 2017; 17(1): 707.
60. Sairenchi T, Iso H, Irie F, Fukasawa N, Ota H, Muto T. Underweight as a predictor of diabetes in older adults: A large cohort study. *Diabetes Care* 2008; 31(3): 583-4.
61. Sakurai M, Ishizaki M, Morikawa Y, et al. Frequency of consumption of balanced meals, bodyweight gain and incident risk of glucose intolerance in japanese men and women: A cohort study. *J Diabetes Investig* 2020; 12(5): 763-70.
62. Sasai H, Sairenchi T, Iso H, et al. Relationship between obesity and incident diabetes in middle-aged and older japanese adults: The ibaraki prefectural health study. *Mayo Clin Proc* 2010; 85(1): 36-40.
63. Schmidt M, Johannesdottir SA, Lemeshow S, et al. Obesity in young men, and individual and combined risks of type 2 diabetes, cardiovascular morbidity and death before 55 years of age: A danish 33-year follow-up study. *BMJ Open* 2013; 3(4).
64. Sheikh MA, Lund E, Braaten T. The predictive effect of body mass index on type 2 diabetes in the norwegian women and cancer study. *Lipids Health Dis* 2014; 13: 164.
65. Siegel LC, Sesso HD, Bowman TS, Lee IM, Manson JE, Gaziano JM. Physical activity, body mass index, and diabetes risk in men: A prospective study. *Am J Med* 2009; 122(12): 1115-21.
66. Song BM, Kim HC, Kim DJ, et al. Aminotransferase levels, body mass index, and the risk of diabetes: A prospective cohort study. *Ann Epidemiol* 2018; 28(10): 675-80.e6.
67. Sui X, Hooker SP, Lee IM, et al. A prospective study of cardiorespiratory fitness and risk of type 2 diabetes in women. *Diabetes Care* 2008; 31(3): 550-5.
68. Sun J, Bao G, Cui J, et al. The association of diabetes risk score and body mass index with incidence of diabetes among urban and rural adult communities in qingdao, china. *International Journal of Diabetes in Developing Countries* 2019; 39(4): 730-8.
69. Tatsumi Y, Ohno Y, Morimoto A, et al. U-shaped relationship between body mass index and incidence of diabetes. *Diabetol Int* 2012; 3(2): 92-8.
70. Twig G, Afek A, Derazne E, et al. Diabetes risk among overweight and obese metabolically healthy young adults. *Diabetes Care* 2014; 37(11): 2989-95.
71. Uemura M, Yatsuya H, Hilawe EH, et al. Breakfast skipping is positively associated with incidence of type 2 diabetes mellitus: Evidence from the aichi workers' cohort study. *J Epidemiol* 2015; 25(5): 351-8.

72. Vaidya A, Cui L, Sun L, et al. A prospective study of impaired fasting glucose and type 2 diabetes in china: The kailuan study. *Medicine (Baltimore)* 2016; 95(46): e5350.
73. Villegas R, Shu XO, Yang G, et al. Energy balance and type 2 diabetes: A report from the shanghai women's health study. *Nutr Metab Cardiovasc Dis* 2009; 19(3): 190-7.
74. Wang B, Zhang M, Wang S, et al. Dynamic status of metabolically healthy overweight/obesity and metabolically unhealthy and normal weight and the risk of type 2 diabetes mellitus: A cohort study of a rural adult chinese population. *Obes Res Clin Pract* 2018; 12(1): 61-71.
75. Wang G, Radovick S, Xu X, et al. Strategy for early identification of prediabetes in lean populations: New insight from a prospective chinese twin cohort of children and young adults. *Diabetes Res Clin Pract* 2018; 146: 101-10.
76. Wang H, Shara NM, Calhoun D, Umans JG, Lee ET, Howard BV. Incidence rates and predictors of diabetes in those with prediabetes: The strong heart study. *Diabetes Metab Res Rev* 2010; 26(5): 378-85.
77. Wang Y, Rimm EB, Stampfer MJ, Willett WC, Hu FB. Comparison of abdominal adiposity and overall obesity in predicting risk of type 2 diabetes among men. *Am J Clin Nutr* 2005; 81(3): 555-63.
78. Wannamethee SG, Shaper AG, Walker M. Overweight and obesity and weight change in middle aged men: Impact on cardiovascular disease and diabetes. *J Epidemiol Community Health* 2005; 59(2): 134-9.
79. Wei Y, Wang J, Han X, et al. Metabolically healthy obesity increased diabetes incidence in a middle-aged and elderly chinese population. *Diabetes Metab Res Rev* 2020; 36(1).
80. Weinstein AR, Sesso HD, Lee IM, et al. Relationship of physical activity vs body mass index with type 2 diabetes in women. *JAMA* 2004; 292(10): 1188-94.
81. Will JC, Williamson DF, Ford ES, Calle EE, Thun MJ. Intentional weight loss and 13-year diabetes incidence in overweight adults. *Am J Public Health* 2002; 92(8): 1245-8.
82. Williams PT, Hoffman K, La I. Weight-related increases in hypertension, hypercholesterolemia, and diabetes risk in normal weight male and female runners. *Arterioscler Thromb Vasc Biol* 2007; 27(8): 1811-9.
83. Xia MF, Lin HD, Chen LY, et al. Association of visceral adiposity and its longitudinal increase with the risk of diabetes in chinese adults: A prospective cohort study. *Diabetes Metab Res Rev* 2018; 34(7): e3048.
84. Ye M, Robson PJ, Eurich DT, Vena JE, Xu JY, Johnson JA. Changes in body mass index and incidence of diabetes: A longitudinal study of alberta's tomorrow project cohort. *Prev Med* 2018; 106: 157-63.
85. Andre P, Proctor G, Driollet B, et al. The role of overweight in the association between the mediterranean diet and the risk of type 2 diabetes mellitus: A mediation analysis among 21 585 uk biobank participants. Int J Epidemiol 2020; 49(5): 1582-90.
86. Bardenheier BH, Wu WC, Zullo AR, Gravenstein S, Gregg EW. Progression to diabetes by baseline glycemic status among middle-aged and older adults in the united states, 2006-2014. Diabetes Res Clin Pract 2021; 174: 108726.
87. Chen Y, Wang N, Dong X, et al. Associations between serum amino acids and incident type 2 diabetes in chinese rural adults. Nutr Metab Cardiovasc Dis 2021; 31(8): 2416-25.
88. Cuthbertson DJ, Koskinen J, Brown E, et al. Fatty liver index predicts incident risk of prediabetes, type 2 diabetes and non-alcoholic fatty liver disease (nafld). Annals of Medicine 2021; 53(1): 1256-64.
89. Hodge AM, Karim MN, Hebert JR, Shivappa N, de Courten B. Association between diet quality indices and incidence of type 2 diabetes in the melbourne collaborative cohort study. Nutrients 2021; 13(11) (no pagination).
90. Narayan KMV, Kondal D, Kobes S, et al. Incidence of diabetes in south asian young adults compared to pima indians. BMJ Open Diabetes Res Care 2021; 9(1).
91. Tang ML, Zhou YQ, Song AQ, Wang JL, Wan YP, Xu RY. The relationship between body mass index and incident diabetes mellitus in chinese aged population: A cohort study. Journal of Diabetes Research 2021; 2021 (no pagination).
92. Xi Y, Gao W, Zheng K, et al. Overweight and risk of type 2 diabetes: A prospective chinese twin study. Diabetes & metabolism 2021: 101278.
93. Xu S, Ming J, Jia A, et al. Normal weight obesity and the risk of diabetes in chinese people: A 9-year population-based cohort study. Sci Rep 2021; 11(1): 6090.
94. Zhu X, Hu J, Guo H, et al. Effect of metabolic health and obesity phenotype on risk of diabetes mellitus: A population-based longitudinal study. Diabetes, Metabolic Syndrome and Obesity: Targets and Therapy 2021; 14: 3485-98.

Table S1. Search strategy (PubMed as an example)

Group	Search term
**Diabetes**	1. "Diabetes Mellitus"[Mesh]
	2. “Hyperglycemia” [Mesh]
	3. “Glucose intolerance” [Mesh]
	4. Diabet*.tw.
	5. T2DM.tw.
	6. NIDDM.tw.
	7. Hyperglyc*.tw.
	8. “Glucose intolerance?”.tw.
	**9. OR/1-8 (n=737,574)**
**Prediabetes**	10. “Prediabetic state” [Mesh]
	11. “Glycated Hemoglobin A” [Mesh]
	12. prediabet*.tw.
	13. “pre diabet*”.tw.
	14. “impaired fasting glucose”.tw.
	15. IFG.tw.
	16. “impaired FPG”.tw.
	17. “impaired glucose tolerance”.tw.
	18. “impaired glucose metabolism”.tw.
	19. IGT.tw.
	20. HbA1c.tw.
	21. Hemoglobin.tw.
	22. “Intermediate hyperglyc*”.tw.
	**23. OR/10-22 (n=191,892)**
**Body mass index**	24. Body mass index [Mesh]
	25. Body mass index.tw.
	26. BMI.tw.
	**27. OR/19-26 (n=292,031)**
**Obese**	28. Overweight [Mesh]
	29. overweight.tw.
	30. obes*.tw.
	**31. OR/28-30 (n=375,582)**
**Non-obese**	32. Thinness [Mesh]
	33. Underweight.tw.
	34. thin*.tw.
	35. lean*.tw.
	36. nonobes*.tw.
	37. “non obes*”.tw.
	38. Nonoverweight.tw.
	39. “non overweight”.tw.
	40. “normal weight”.tw.
	**41. OR/32-40 (n=391,652)**
**Cohort study**	42. “Cohort studies” [Mesh]
	43. Cohort.tw
	44. Prospective.tw.
	45. Longitudinal.tw.
	46. Follow-up.tw.
	47. Inciden*.tw.
	**48. OR/41-46 (n=3,453,763)**
**Combine**	**49. (9 OR 23) AND 27 AND (31 OR 41) AND 48 (n=10,286)**
**Filter**	**Filter in English and Human studies and adults (> 18 years) (n=9,171)**

Table S2. Study quality of included studies assessed by NOS

First author	Year	Selection (0-4)	Comparability (0-2)	Outcome (0-3)	Total
Appleton, S. L.	2013	4	1	2	7
Arnlov, J.	2011	4	1	2	7
Asghar, S.	2011	4	2	3	9
Asvold, B. O.	2017	4	2	2	8
Aung, K.	2014	4	2	2	8
Beleigoli, A. M.	2020	3	2	2	7
Borné, Y.	2015	4	2	3	9
Bragg, F.	2018	4	2	3	9
Carey, V. J.	1997	2	2	1	5
Chan, J. C. Y.	2018	4	2	1	7
Chan, J. M.	1994	2	1	2	5
Chang, Y.	2016	4	2	2	8
de Mutsert, R.	2014	2	2	2	6
Dotevall, A.	2004	4	2	3	9
Dow, C.	2016	3	2	2	7
Dowse, G. K.	1996	4	0	2	6
Edwards, M. K.	2017	4	2	2	8
Feller, S.	2010	4	2	1	7
Feng, S.	2020	4	2	1	7
Field, A. E.	2004	2	2	1	5
Ford, E. S.	1997	3	2	2	7
Fox, C. S.	2006	4	1	2	7
Fujita, M.	2009	4	2	2	8
Gautier, A.	2009	3	2	2	7
Giraldez-Garcia, C.	2018	4	2	1	7
Hadaegh, F.	2006	4	2	1	7
Han, C.	2017	4	2	2	8
Hara, H.	1996	3	1	2	6
Hayashi, T.	1999	4	2	3	9
Hinnouho, G. M.	2015	4	2	2	8
Holtermann, A.	2017	4	2	2	8
Hu, F. B.	2001	2	2	2	6
Hu, G.	2004	4	2	2	8
Hu, G.	2005	4	2	2	8
Hu, H.	2016	4	2	3	9
Ishikawa-Takata, K.	2002	4	2	2	8
Jae, S. Y.	2016	4	2	2	8
Janghorbani, M.	2017	3	1	3	7
Jiamjarasrangsi, W.	2005	4	2	1	7
Jung, C. H.	2015	4	2	1	7
Jung, H. H.	2017	4	2	3	9
Jung, J. Y.	2018	4	2	2	8
Krishnan, S.	2007	3	2	1	6
Lee, D. H.	2018	2	2	1	5
Li, F.	2020	3	2	2	7
Li, W. D.	2015	2	2	2	6

Lim, J. S.	2007	4	2	3	9
Manson, J. E.	1991	2	1	1	4
Maty, S. C.	2005	3	2	3	7
Meigs, J. B.	2006	4	2	2	8
Meisinger, C.	2006	4	2	1	7
Mishra, G. D.	2007	3	2	1	6
Nagayoshi, M.	2016	3	2	2	7
Nanri, A.	2010	3	2	1	6
Nguyen, B.	2017	3	2	0	5
Ning, F.	2020	4	2	2	8
Oguma, Y.	2005	2	2	1	5
Ohlsson, C.	2019	4	1	3	8
Papier, K.	2017	3	2	2	7
Sairenchi, T.	2007	4	2	2	8
Sakurai, M.	2020	4	2	1	7
Sasai, H.	2010	4	2	3	9
Schmidt, M.	2013	4	1	3	8
Sheikh, M. A.	2014	3	1	1	5
Siegel, L. C.	2009	2	2	1	5
Song, B. M.	2018	4	2	1	7
Sui, X.	2008	4	2	2	8
Sun, J.	2019	4	2	2	8
Tatsumi, Y.	2012	3	2	2	7
Twig, G.	2014	3	2	2	7
Uemura, M.	2015	4	2	2	8
Vaidya, A.	2016	4	2	1	7
Villegas, R.	2009	4	2	1	7
Wang, B.	2017	4	2	2	8
Wang, G.	2018	4	2	2	8
Wang, H.	2010	4	2	3	9
Wang, Y.	2005	2	2	1	5
Wannamethee, S. G.	2005	4	2	2	8
Wei, Y.	2020	4	2	1	7
Weinstein, A. R.	2004	3	2	2	7
Will, J. C.	2002	3	2	1	6
Williams, P. T.	2007	3	2	1	6
Xia, M. F.	2018	4	0	1	5
Ye, M.	2018	3	2	3	8
Andre, P.	2020	3	2	1	6
Bardenheier, B. H.	2021	4	2	1	7
Chen, Y.	2021	4	2	2	8
Cuthbertson, D. J.	2021	4	1	2	7
Hodge, A. M.	2021	4	2	2	8
Narayan, K. M. V.	2021	4	0	1	5
Tang, M. L.	2021	4	2	2	8
Xi, Y.	2021	3	2	0	5
Xu, S.	2021	4	2	2	8
Zhu, X.	2021	4	2	1	7

NOS: Newcastle-Ottawa Scale.

Table S3. The characteristics of included studies reported diabetes incidence

Author	Publication year	Country /region	Median year[a]	Follow-up (year range, duration)	Participants (n, age, female %)	Weight measurement	Diabetes measurement[b]	Incidence (n for cases; % for cumulative incidence; PY, person-years for incidence rate)
Appleton, S. L.	2013	Australia	2005	1999-2003 to 2008-2010<br>Mean 7.9 y	n=3743<br>45.7 years; Female: 52%	Measured directly	Multiple	<25, n= 16; 16/739=2.2%; 2.2/0.79=2.7 1000 PY<br>25~30, n= 39; 39/925=4.2%; 4.2/0.79= 5.3 1000 PY<br>>30, n=57; 57/756=7.5%; 7.5/0.79=9.5 1000 PY
Arnlov, J.	2011	Sweden	1981.5	1970-1973 to 1990-1993<br>20 y	n=1675<br>50 y; Female: 0%	Measured directly	Multiple	<25, n=32+7=39; 39/(853+60)=4.3%; 4.3/2=2.1 1000 PY<br>25~30, n=67+27=94; 94/(557+117)=13.9%; 13.9/2=7.0 1000 PY<br>>30, n=9+18=27; 27/(29+60)=30.3%; 30.3/2=15.2 1000 PY
Asghar, S.	2011	Bangladesh	2001.5	1999 to 2004<br>5 y	n=2011<br>Mean 36.2 y; Female: 66%	Measured directly	Biology test	Female<br><18.4, n=23; 23/353=6.5%; 13 1000 PY<br>18.5–23.0, n=48; 48/670=7.2%; 14.4 1000 PY<br>>23.0, n =29; 29/310=9.3%, 18.6 1000 PY<br>Male (calculated by total-female)<br><18.4, n=18; 18/225=8.0%, 16 1000 PY<br>18.5–23.0, n=29; 29/348=7.8.3%, 15.6 1000 PY<br>>23.0, n =18; 18/135=13.3%, 26.6 1000 PY
Asvold, B. O.	2017	Norway	2001.5	1995-1997 to 2006-2008<br>11years	n=28051<br>Median 45 y; Female:55%	Measured directly	Multiple	<30, n=702 cases; 702/268428=2.6 1000 PY<br>>30, n =548 cases; 548/40032=13.7 1000 PY
Aung, K.	2014	America	1988.0	1979-1988 to 1987-1996<br>Median 7.4 y	n=2011<br>25-64 y; Female: 55%	Measured directly	Biology test	<25, n=30, 30/1110=2.7%; 2.7/0.74=3.7 1000 PY<br>25-30, n=96, 96/1067=9.0%; 9.0/2.74=12.2 1000 PY<br>>30, n=136, 136/637=21.3%; 21.3/0.74=28.9 1000 PY
Beleigoli, A. M.	2020	Australia	2006.5	1999-2003 to 2015<br>Mean 13.3 y	n= 3039<br>45.2 y; Female: 52%	Measured directly	Multiple	<25, n=26; 26/1037=2.5%; 2.5/1.33=1.9 1000 PY<br>25-30, n=94; 94/1203=7.8%; 7.8/1.33=5.9 1000 PY<br>>30, n=116; 116/800=14.5%; 14.5/1.33=10.9 1000 PY
Borné, Y.	2015	Sweden	2001.3	1991-1996 to 2009<br>14 y	n=26604<br>45-73 y; Female: 61%	Measured directly	Medical records	Males<br>13.9-24.0, n=170; 170/2583=6.6%; 4.7 1000 PY<br>24.0–26.0, n=228; 228/2587=8.8%; 6.2 1000 PY<br>26.0–28.0, n=376; 376/2583=14.6%; 10.7 1000 PY<br>>28.0, n =745; 745/2577=28..9%; 23.7 1000 PY<br>Females<br>13.9-22.4 n=120; 120/4058=3.0%; 2.0 1000 PY<br>22.4–24.6, n=196; 196/4090=4.8%; 3.3 1000 PY<br>24.6–27.5, n=346; 346/4067=8.5%; 5.9 1000 PY<br>>27.5, n =754; 754/4057=18.6%; 13.8 1000 PY
Bragg, F.	2018	China	2009.8	2004-2008 to 2013-2014<br>9.2 y	n=482589<br>30-79 y; Female: 59%	Measured directly	Medical records	Males<br><18.5, n=104; 104/9108=1.1%; 1.1 1000 PY<br>18.5–24.9, n=2183; 2183/129855=1.7%; 1.95 1000 PY<br>25–29.9, n=2366; 2366/54161=4.4%; 5.4 1000 PY

								>30, n =431; 431/5450=7.9%; 10.6 1000 PY<br>Females<br><18.5, n=182; 182/12528=1.45%; 1.18 1000 PY<br>18.5–24.9, n=3492; 3492/177202=1.97%; 2 1000 PY<br>25–29.9, n=3680; 3680/81426=4.52%; 5.29 1000 PY<br>>30, n =978; 978/12859=7.61%; 10.48 1000 PY
Carey, V. J.	1997	America	1990.0	1986 to 1994<br>8 y	n=42492<br>30-55 y; Female: 100%	Self-report	Self-report	<21, n=21; 21/63317=0.3 1000 PY, 0.24%<br>21-22.9, n=40; 40/82395=0.5 1000 PY, 0.4%<br>23-24.9, n=82; 82/73042=1.1 1000 PY, 0.88%<br>25-26.9, n=90; 90/45066=2.0 1000 PY, 1.6%<br>27-28.9, n=96; 96/27775=3.5 1000 PY, 2.8%<br>29-30.9, n=112; 112/17970=6.2 1000 PY, 5.0%<br>>31, n=264; 264/23819=11.1 1000 PY, 8.9%
Chan, J. C. Y.	2018	Singapor e	2009.8	2004-2006 to 2011-2015<br>Median 6.2 y	n=2403<br>Mean 54.5 y; Female:52%	Measured directly	Biology test	Total<br><25, n=73, 6.7%, 10.81 1000 PY<br>25-30, n=153, 16.4%, 26.45 1000 PY<br>>30, n=82, 21.5%, 36.68 1000 PY<br>Male<br><25, n=43, 7.2%, 11.61 1000 PY<br>25-30, n=77, 17.0%, 27.42 1000 PY<br>>30, n=25, 24.8%, 40 1000 PY<br>Female<br><25, n=30, 6.1%, 9.84 1000 PY<br>25-30, n=76, 15.9%, 26.64 1000 PY<br>>30, n=57, 20.4%, 32.90 1000 PY
Chan, J. M.	1994	America	1988.0	1986 to 1992<br>5 y	n=27983<br>40-75 y, Female: 0%	Self-report	Self-report	<23, n=17; 17/25488=0.67 1000 PY, 0.34%<br>23-24, n=13; 13/20190=0.64 1000 PY, 0.32%<br>24-25, n=20; 20/18384=1.1 1000 PY, 0.55%<br>25-27, n=55; 55/34230=1.6 1000 PY, 0.8%<br>27-29, n=57, 57/17402=3.3 1000 PY, 1.7%<br>29-31, n=38, 38/7427=5.1 1000 PY, 2.6%<br>31-33, n=27; 27/3140=8.6 1000 PY, 4.3%<br>33-35, n=21; 21/3151=6.7 1000 PY, 3.4%<br>>35, n=24; 24/870=27.6 1000 PY, 13.8%
Chang, Y.	2016	South Korea	2009.0	2005 to 2013<br>Mean 4.1 y	n=74509<br>Mean 36.2 y; Female: 58%	Measured directly	Biology test	<18.5, n=20; 0.8 1000 PY<br>18.5–22.9, n=239; 239/(181725.2+5704.6)=1.28 1000 PY<br>23-24.9, n=119; 119/(50234.6+8838.3)=2.01 1000 PY<br>>25, n=94; 94/(22288.2+10615.7)=2.86 1000 PY
de Mutsert, R.	2014	America	1997.0	1986 to 2008<br>22 y	n=39909<br>40-75 y; Female: 56.8%	Self-report	Self-report	<18.5, n=113; 113/1296=8.7%, 3.95 1000 PY<br>18.5-22.9, n=1080; 1080/19065=5.7%, 2.59 1000 PY<br>23.0-24.9; n=613; 613/10669=5.7%, 2.59 1000 PY

								25.0-27.4, n=603; 603/6674=9.0%, 4.09 1000 PY<br>27.5-29.9, n=207; 207/1491=13.9%, 6.32 1000 PY<br>>30, n=119; 119/714=16.7%; 7.59 1000 PY
Dotevall, A.	2004	Sweden	1988.8	1979-1981 to 1997-1998<br>18 y	n=1351<br>39-65 y; Female: 100%	Measured directly	Multiple	<22, n=6; 0.88 1000PY, 6/366=1.64%<br>22-24, n=7; 1.09 1000 PY, 7/338=2.07%<br>24-27, n=19; 2.98 1000 PY, 19/348=5.46%<br>>27, n=41; 7.82 1000 PY, 41/298=13.76%
Dow, C.	2016	France	2002.0	1993 to 2011<br>18 y	n=71334<br>Median 52.9 y; Female: 100%	Measured directly	Self-report	<20, n=68; 0.66%, 0.37 1000 PY<br>20-24.9, n=1028; 2.2%, 1.2 1000 PY<br>25-30, n=982; 8.5%, 4.72 1000 PY<br>>30, n=532; 10.6%, 5.89 1000 PY
Dowse, G. K.	1996	Pacific and Indian Ocean	1985.0	1978 to 1992<br>Average 8 y	n=4731<br>25-54 years; Female: NR	Measured directly	Multiple	<25, n=(26*19.7*7.8+38*5.2*5.1+1813*9.0*5.0)/1000=86; (26*19.7+38*5.2+1813*9.0)/(26+118+79+38+1813)=8.21 1000 PY<br>25-30, n=(21*36.5*7.8+25*6.0*7.1+106*7.9*13.2+110*5.3*5.1+690*18.6*5.0)/1000=84; (21*36.5+25*6.0+106*7.9+110*5.3+690*18.6)/(21+25+106+110+690)=15.94 1000 PY<br>>30, n=(13*49.2*7.8+86*18.5*13.2+412*29.0*5.3+159*31.4*5.0)=113; (13*49.2+86*18.5+412*29.0+159*31.4)/(13+4+86+412+159)=28.44 1000 PY
Edwards, M. K.	2017	America	2006.8	2001-2004 to 2009-2013<br>8 y	n=2450<br>Average 50 y; Female: 47%	Measured directly	Biology test	18.5-25, n=2+3+6+5=16; 16/366=4.4%, 4.4/0.8= 5.5 1000 PY<br>25-30, n=14+22+20+20=73; 76/850=8.9%, 8.9/0.8=11.13 1000 PY<br>>30, n =27+35+49+83=194; 194/1234=15.7%, 15.7/0.8=19.63 1000 PY
Feller, S.	2010	German	2000.0	1994-1998 to NR<br>Mean 8 y	n=25244<br>35-65 y; Female 61%	Measured directly	Self-report	Male<br><25, n=45; 45/2965=1.5%, 1.5/0.8=1.88 1000 PY<br>25-30, n=2287; 287/5182=5.5%, 5.5/0.8=6.88 1000 PY<br>>30, n=257; 257/1606=16.0%, 16/0.8=20.0 1000 PY<br>Female<br><25, n=48; 48/8055=0.6%, 0.6/0.8=0.75 1000 PY<br>25-30, n=151; 151/5007=3.0%, 3.0/0.8=3.75 1000 PY<br>>30, n=226; 226/2429=9.3%, 9.3/0.8=11.63 1000 PY
Feng, S.	2020	China	2016.0	2014 to 2018<br>4 y	n=49702<br>≥60 y; ; Female 56%	Measured directly	Multiple	<24.0, n=250+119=369;362/22612=1.6%; 369/(71024+19005)=4.1 1000 PY<br>24.0–28.0, n=251+201=452; 452/20224=2.2%;

								452/(53970+26525)=5.6 1000 PY<br>>28.0, n =92+130=222; 222/6866=3.2%;<br>222/(14848+12487)=8.1 1000 PY
Field, A. E.	2004	America	1996.0	1993 to 1999<br>6 y	n=46634<br>25-43 y; Female: 100%	Self-report	Self-report	<22, n= 9; 9/38812=0.23 1000 PY<br>22-24.9, n=17; 17/38408=0.44 1000 PY<br>25-29.9, n=69; 69/32391=2.1 1000 PY<br>30-35, n=104, 104/13956=7.4 1000 PY<br>>35, n=219, 219/9954=22.0 1000 PY
Ford, E. S.	1997	America	1980.5	1971-1975 to 1984-1992<br>9 y	n=8545<br>25-74 y; Female: 62.3%	Measured directly	Self-report	<22, n=35, 35/19116=1.59 1000 PY<br>22-22.9, n=16, 16/7090=1.96 1000 PY<br>23-23.9, n=33, 33/6699=4.28 1000 PY<br>24-24.9, n=29, 29/6376=5.92 1000 PY<br>25-26.9, n=73, 73/11600=5.55 1000 PY<br>27-28.9, n=88, 88/8265=9.51 1000 PY<br>29-30.9, n=72, 72/4996=10.95 1000 PY<br>31-32.9, n=43, 43/2958=14.78 1000 PY<br>33-34.9, n=40, 40/1718=19.82 1000 PY<br>≥35, n=58, 58/2104=26.36 1000 PY
Fox, C. S.	2006	America	1980.0	1970s to 1990s<br>8 y	n=3104<br>Mean 47 y; Female: 51%	Measured directly	Biology test	<25, n=1; 1/540=0.2%. 0.2/0.8=0.25 1000 PY<br>25-30, n=16; 16/566=2.5%, 2.5/0.8=3.13 1000 PY<br>>30, n=13; 13/203=6.4%, 6.4/0.8=8.0 1000 PY
Fujita, M. (Chiba)	2009	Japan	1999.5	1994 to 2005<br>12 y	n=35579<br>40-79 y; Female: 79%	Measured directly	Biology test	Male<br><18.5; n=2+26+17=45; 45/(402+1219+985)=17.3 1000 PY<br>18.5-25, n=181+327+46=554;<br>554/(15181+18229+2697)=15.3 1000 PY<br>>25, n=160+160+68=388; 388/(5866+5943+2514)=27.1 1000 PY<br>Female<br><18.5; n=39+25+19=83; 83/(9901+3219+1940)=5.5 1000 PY<br>18.5-25, n=675+340+165=1180;<br>1180/(134764+39934+13796)=6.3 1000 PY<br>>25, n=401+247+95=743; 743/(26885+12635+4985)=16.7 1000 PY
Fujita, M. (Kashiwa)	2009	Japan	2004.0	2002 to 2006<br>5 y	n=27760<br>40-79 y; Female: 74%	Measured directly	Biology test	Male<br><18.5; n=1+6+18=25; 25/(73+339+548)=26.0 1000 PY<br>18.5-25, n=46+192+181=419; 419/(2697+8424+6982)=23.1 1000 PY<br>>25, n=43+103+83=229; 229/(1120+3015+2051)=37.0 1000 PY<br>Female

								<18.5; n=10+9+23=42; 42/(2091+1518+999)=9.1 1000 PY<br>18.5-25, n=115+209+148=472; 472/(26533+20019+9569)=8.4 1000 PY<br>>25, n=91+112+82=285; 285/(5411+5437+2980)=20.6 1000 PY
Gautier, A.	2010	France	1999.5	1994-1996 to 2003-2005<br>9 y	n=3826<br>aged 30-64 years general population (51% for females)<br>1,865 men and 1,962 women	Measured directly	Biology test	Total<br><27, n=92; 92/2947=3.1%, 3.1/0.9=3.4 1000 PY<br>>27, n=111; 111/879=12.6%, 12.6/0.9= 14 1000 PY<br>Male<br><27, n=69, 69/1370=5.0%, 5.0/0.9=5.6 1000 PY<br>>27, n=71, 71/494=14.4%, 14.4/0.9=16 1000 PY<br>Female<br><27, n=23, 23/1577=1.5%, 1.5/0.9=1.67 1000 PY<br>>27, n=40, 40/395=10.1%, 14.4/0.9=16 1000 PY
Giraldez-Garcia, C.	2018	Spain	2013.5	2012 to 2015<br>3 y	n=1184<br>30-74 y; Female: 50%	Measured directly	Biology test	30-59 y<br><30, n=28; 9.1%; 28/879= 31.8 1000 PY<br>>30, n=38; 15.8%; 38/689=55.1 1000 PY<br>60-74 y<br><30, n=33; 9.0%; 33/1038= 31.8 1000 PY<br>>30, n=44; 16.4%; 44/755=58.3 1000 PY
Hadaegh, F.	2006	Iran	2002.5	1999-2001 to 2005<br>Median 3.8 y	n=1852<br>Mean 45.1 y; Female: 0%	Measured directly	Biology test	<22.9, n=69*1.8/(1.8+1.9+4.2+6.4)=8; 1.8%; 1.8/0.38=4.74 1000 PY<br>23-25.9, n=69*1.9/(1.8+1.9+4.2+6.4)=9; 1.9%, 1.9/0.38=5 1000 PY<br>26.0–27.9, n=69*4.2/(1.8+1.9+4.2+6.4)=21; 4.2%, 4.2/0.38=11.05 1000 PY<br>>28.0, n=69*6.4/(1.8+1.9+4.2+6.4)=31; 6.4%, 6.4/0.38=16.84 1000 PY
Han, C.	2017	China	2010.5	2007-2008 to 2013-2014<br>6 y	n=11687,<br>Median 51 y; Female: 62.4%	Measured directly	Self-report	Q1 (18.51–21.49), n=60; 4.25 1000 PY<br>Q2 (21.50–23.33), n=92; 6.61 1000 PY<br>Q3 (23.34–25.10), n=114; 8.21 1000 PY<br>Q4 (25.11–27.28), n=189; 13.70 1000 PY<br>Q5 (27.29–44.56), n=294; 21.74 1000 PY
Hara, H.	1996	America	1982.5	1976 to 1989<br>9 y	n=1144<br>Median 60.7 y; Female: 58%	Measured directly	Biology test	<19, n=1; 1.4%; 2.1 1000 PY<br>19-21, n=11; 6.0%; 9.1 1000 PY<br>21-23, n=9; 4.3%; 6.5 1000 PY<br>23-25, n=9; 4.5%; 6.8 1000 PY<br>25-27, n=8; 7.4%; 11.4 1000 PY<br>>27, n=8; 11.3%; 18.4 1000 PY

Hayashi, T.	1999	Japan	1989.0	1981 to 1997<br>9.6 y	n=7594<br>35-61 y; Female: 58%	Measured directly	Biology test	<22.7, n=103+50+1=154; 154/(25542+7370+3998)=4.2 1000 PY<br>>22.7, n=197+106+113=416; 416/(19842+9262+6932)=11.5 1000 PY
Hinnouho, G. M.	2015	NR	UK	1991–1993 to 2008-2009<br>18 y	n=7122<br>Mean 49.3 y; Female: 30%	Measured directly	Multiple	<25, n=154+110=264; 264/(154/3.37+110/12.46)=4.84 1000 PY<br>25~30, n=136+222=358; 358/(136/5.75+222/15.29)=9.38 1000 PY<br>30>, n=48+128=176; 176/(48/12.96+128/26.58)=20.66 1000 PY
Holterman n, A.	2017	Denmark	1992.3	1970-1971 to 2014,<br>44 y	n=4988<br>Mean 48.7 y; Female: 0%	Measured directly	Medical records	<25, n=136; 136/2388=5.7%, 5.7/4.4=1.29 1000 PY<br>25-30, n=301; 301/2266=13.3%, 13.3/4.4=3.02 1000 PY<br>>30, n =81; 81/334=24.2%, 24.2/4.4=5.5 1000 PY
Hu, F. B.	2001	America	1988.0	1980 to 1996<br>16 y	n=84941<br>30-55 y; Female: 100%	Self-report	Self-report	<23, n=121; 121/(0.32*1301055)=0.3 1000 PY<br>23-24.9, n=202; 202/(0.18*1301055)=0.9 1000 PY<br>25-29.9, n=884; 884/(0.25*1301055)=2.7 1000 PY<br>30-34.9, n=885; 885/(0.09*1301055)=7.6 1000 PY<br>>35, n=759; 759/(0.04*1301055)=14.6 1000 PY
Hu, G.	2004	Finland	1988.3	1982-1987 to 1992<br>9.4 y	n=4369<br>45-64 y; Female: 54%	Measured directly	Medical records	<25, n=11; 11/12208=0.9 1000 PY<br>25-30, n=38; 38/19291=2.0 1000 PY<br>>30, n =71; 71/9801=7.2 1000 PY
Hu, G.	2005	Finland	1993.3	1982-1987 to 2002<br>18.1 y	n=1935<br>34-64 y; Female: 52%	Measured directly	Medical records	<30, n=57; 57/(5897+22239)=2.0 1000 PY<br>>30, n=72; 72/(2605+4285)=10.4 1000 PY
Hu, H.	2017	Japan	2011.5	2008 to 2015<br>7 y	n=51777<br>30-59 y; Female: 14%	Measured directly	Biology test	<23, n=964; 964/26048=3.7%, 3.7/7=0.53 1000 PY<br>23-27.5, n=1666; 1666/20986=7.9%, 7.9/7=1.13 1000 PY<br>>27.5, n =835; 835/4743=17.6%, 17.6/7=2.51 1000 PY
Ishikawa-Takata, K.	2002	Japan	1996.0	1994 to 1998<br>10 y	n=4737<br>18-59 y; Female: 0%	Measured directly	Biology test	<23, n=108; 108/9903= 10.9 1000 PY, 10.9%<br>23-25, n=61; 61/3688=16.5 1000 PY, 16.5%<br>>25, n=74; 74/2838=26.1 1000 PY, 26.1%
Jae, S. Y.	2016	South Korea	2003.5	1998 to 2009<br>Median 5 y	n=3770<br>20-76 y; Female: 0%	Measured directly	Biology test	18.5–24.9, n=80; 80/2309=3.5%, 7 1000 PY<br>25.0–29.9, n=84; 84/1409=6.0%, 12 1000 PY<br>>30, n =6; 6/52=11.5%, 23 1000 PY
Janghorba ni, M.	2017	Iran	2010.0	2003-2005 to 2016<br>Mean 10.1 y	n=1959<br>30-70 y; Female: 76%	Measured directly	Biology test	<25, n=36; 36/312=11.5%; 36/(817+2279)=11.6 1000PY<br>25-29.9, n=161; 161/942=17.1%; 161(1326+8165)=17.0 1000PY<br>>30, n=166; 166/705=23.5%; 166/(797+6351)=23.2 1000PY
Jiamjarasra ngsi, W.	2005	Thailand	2001.0	1999 to 2003<br>Mean 1.67 y	n=6924<br>35-59 y; Female: 69%	Measured directly	Biology test	<18.5, n=0; 0/270=0; 0/450=0<br>18.5–22.9, n=23; 23/2680=0.9%; 23/4376= 5.3 1000 PY

								23.0-27.4, n =47; 47/2479=1.9%; 47/3965= 11.8 1000 PY<br>>27.5, n=43; 43/855=5.0%; 43/1345=32.0 1000 PY
Jung, C. H.	2015	South Korea	2010.0	2007 to 2013<br>Median 3 y	n=36135<br>Mean 47.5 y;<br>Female: 42%	Measured directly	Multiple	<25, n=145+133=278, 278/(4696+20491)=1.1%, 1.1/0.3=3.7 1000 PY<br>>25, n=269+88=357, 357/(4909+6039)=3.3%, 3.3/0.3=10.9 1000 PY
Jung, H. H.	2017	South Korea	2008.5	2002 to 2015<br>10 y	n=850282<br>40-79 y; Female: 42%	Measured directly	Medical records	16.0–18.4, n=513; 2.8%; 3.2 1000 PY<br>18.5–19.9, n=1544; 3.1%; 3.4 1000 PY<br>20.0–22.9, n=12950; 5%; 5.3 1000 PY<br>23.0–24.9, n=19179; 8.2%; 8.8 1000 PY<br>25.0–27.4, n=23520; 11.8%; 13 1000 PY<br>27.5–29.9, n=11520; 16.7%; 19 1000 PY<br>30.0–32.4, n=3605; 21.8%; 25.7 1000 PY<br>32.5–34.9, n=925; 26.5%; 32.5 1000 PY
Jung, J. Y.	2018	South Korea	2006.5	2001-2002 to 2011-2012<br>10 y	n=7660<br>Mean 51.7 y;<br>Female: 53%	Measured directly	Biology test	Male<br><18.5, n=18; 28.7 1000 PY<br>18.5–23.0, n=186; 19.6 1000 PY<br>23-25, n=189; 23.5 1000 PY<br>25-30, n=301; 27.8 1000 PY<br>>30.0, n =31; 41.6 1000 PY<br>Female<br><18.5, n=10; 22.2 1000 PY<br>18.5–23.0, n=148; 15.0 1000 PY<br>23-25, n=173; 19.3 1000 PY<br>25-30, n=319; 25.3 1000 PY<br>>30.0, n =65; 31.4 1000 PY
Krishnan, S.	2007	America	1999.0	1995 to 2003<br>8 y	n=49766<br>21-69 y; Female: 100%	Self-report	Self-report	<25, n=50+100=150; 150/1960=7.6%, 150/(29113+23801)= 2.8 1000 PY<br>25-29, n=646; 646/15851=4.1%; 646/55179=11.7 1000PY<br>30-34, n=666; 666/8003=8.2%; 666/30198=22.0 1000 PY<br>35-39, n=511; 511/3589=14.2%; 511/13841=36.9 1000 PY<br>≥40, n=162+235=397; 397/2722=14.6%; 397/(6498+4260)=36.9 1000 PY
Lee, D. H.	2018	America	1995.0	HPFS (1978 to 2012<br>34 y)	HPFS: n=36925<br>40–75 y; Female: 0%	Self-report	Self-report	HPFS:<br>Q1: n=159, 159/133686=1.19 1000 PY<br>Q2, n=282, 282/138025=2.04 1000 PY<br>Q3, n=412, 412/134151=3.07 1000 PY<br>Q4, n=632, 632/135411=4.67 1000 PY<br>Q5, n=1306, 1306/133978=9.75 1000 PY

Lee, D. H.	2018	America	1999.0	NHS (1986 to 2012<br>26 y)	NHS: n=60186<br>30–55 y: Female: 100%	Self-report	Self-report	NHS:<br>Q1: n=215, 215/242898=0.88 1000 PY<br>Q2, n=386, 386/243011=1.59 1000 PY<br>Q3, n=672, 672/242908=2.77 1000 PY<br>Q4, n=1207, 1207/242340=4.98 1000 PY<br>Q5, n=2722, 2722/24107911.29 1000 PY
Li, F.	2020	China	2013.5	2011 to 2016<br>5 y	n=4556<br>45.1 y; Female: 46%	Measured directly	Multiple	<24, n=37; 1.5%, 1.5/0.5=3 1000 PY<br>24-28, n=84; 5.6%, 5.6/0.5=11.2 1000 PY<br>>28, n=49; 8.5%, 8.5/0.5=17 1000 PY
Li, W. D.	2015	China	2010.0	2008 to 2012<br>median 3.85 y	n=4736<br>Mean 52.7 y; Female: 66.5%	Measured directly	Biology test	<24.0, n=166; 5.5%, 5.5/0.385=14.29 1000 PY<br>24.0–28.0, n=137; 11.0%, 11/0.385=28.57 1000 PY<br>>28.0, n =77; 15.8%, 15.8/0.385=41.04 1000 PY
Lim, J. S.	2007	America	1991.0	1988 to 1994<br>6 y	n=3629<br>≥40 y: Female: NR	Measured directly	Biology test	<25, n=34; 34/1236=2.7%, 2.7/0.6=4.5 1000 PY<br>25-30, n=72; 72/1388=5.2%, 5.2/0.6=8.7 1000 PY<br>30-35, n=60; 60/672=8.9%, 8.9/0.6=14.8 1000 PY<br>>35, n=39; 39/333=11.7%, 11.7/0.6=19.5 1000 PY
Manson, J. E.	1991	America	1983.5	19761984 to 1986-1988<br>8 y	n= 82753<br>34-59 y: Female: 100%	Self-report	Self-report	<27, n=241+150=391, 391/(277309+251203)=0.74 1000 PY<br>≥27, n=603+309=912, 912/(85475+56410)=6.43 1000 PY
Maty, S. C.	2005	America	1982.0	1965 to 1999<br>34 y	n=6147<br>Median 58.6 y; Female: 54%	Self-report	Self-report	<25, n=154; 3.7%, 1.7 1000 PY<br>25-30, n=106; 6.4%, 3.0 1000 PY<br>>30, n=58; 16.9%, 8.7 1000 PY
Meigs, J. B.	2006	America	1996.3	1991-1995 to 1998-2001<br>7 y	n=2902<br>Mean 53 y; Female: 50%	Measured directly	Biology test	Total<br><25, n=12+5=17; 17/1056=1.6%, 1.6/0.7=2.29 1000 PY<br>25-30, n=13+37=50; 50/1208=4.1%, 4.1/0.7=5.86 1000 PY<br>>30, n=7+67=74; 74/638=11.6%, 11.6/0.7=16.57 1000 PY
Meisinger, C.	2006	German	1996.0	1989-1990 to 1997-1998 or 1994-1995 to 2002-2003<br>Mean 9.2 y	n=6462<br>35–74 y; Female: 46%	Measured directly	Self-report	Male<br><25.1, n=21, 21/6711=3.1.3 1000 PY, 21/727=2.9%<br>25.1-27.2, n=37, 37/7297=5.07 1000 PY, 37/800=4.6%<br>27.2-29.4, n=59, 59/6631=8.9 1000 PY, 59/739=8.0%<br>>29.4, n=126, 126/6598=19.1 1000 PY, 126/789=16.0%<br>Female<br><23.4, n=4, 4/7279=0.55 1000 PY, 4/734=0.5%<br>23.4-26.1, n=21, 21/7255= 2.89 1000 PY, 21/739=2.8%<br>26.1-29.5, n=39, 39/6955=5.61 1000 PY. 39/737=5.3%<br>>29.5, n=94, 94/6550=14.35 1000 PY, 94/747=12.6%
Mishra, G. D.	2007	Australia	2008.5<br>2010	1996 to 2001-2004<br>8 y	n=7239<br>Mean 47.5 y; Female: 100%	Self-report	Self-report	<25, n=3985*0.005=20; 0.5%, 0.5/0.8=0.63 1000 PY<br>25-30, n=2064*0.021=43; 2.1%, 2.1/0.8=2.63 1000 PY<br>30-35, n=815*0.044=36; 4.4%, 4.4/0.8=5.5 1000 PY<br>>35, n=375*0.057=21; 5.7%, 5.7/0.8=7.13 1000 PY

Nagayoshi, M.	2016	America	2004.5	1996-1998 to 2011-2013<br>Median 13	n=1453<br>Mean 63 y: Female: 54%	Measured directly	Self-report	<30, n=154; 154/1001=15.4%; 154/11446=13.4 1000 PY<br>>30, n=131; 131/452=29.0%; 131/4728=27.7 1000 PY
Nanri, A.	2010	Japan	1996.5	1990-1993 to 2000-2003<br>5 y	n=33045<br>45-75 y;<br>Female:57%	Self-report	Self-report	<25, n=219; 219/23586=0.9%, 0.9/0.5=1.8 1000 PY<br>>25, n=249; 249/9459=2.6%, 2.6/0.5= 5.2 1000 PY
Nguyen, B.	2017	Australia	2008.5	2006-2008 to 2010<br>median 2.7 y	n=29572<br>≥45 y; Female: 55%	Self-report	Self-report	18.5-25, n=0.195*611=119; 119/12450=0.96%, 0.96/0.27=3.6 1000 PY<br>25-30, n=0.412*611=252; 252/ 12124=2.08%, 2.08/0.27=7.7 1000 PY<br>>30, n=0.393*611=240; 240/4998=4.80%, 4.8/0.27=17.8 1000 PY
Ning, F.	2020	China	2009.0	2006 to 2009<br>or<br>2009 to 2012<br>3 y	n=2809<br>35-74 y; Female: 62%	Measured directly	Biology test	<30, n=221; 221/2531=8.7%, 8.7/0.3=29 1000 PY<br>>30, n=54; 54/278=19.4%, 19.4/0.3=64.7 1000 PY
Oguma, Y.	2005	America	1981.0	1962 or 1966 to 1998<br>32 or 36 y	n=20187<br>Mean 45.9; Female: 0%	Self-report	Self-report	<21; n=36; 36/1171=3.1%; 36/27866=1.3 1000 PY<br>21-23; n=138; 138/3977=3.5%; 138/97049=1.4 1000 PY<br>23-25; n=325; 325/7146=4.5%; 325/173548=1.9 1000 PY<br>25-27; n=363; 363/5067=7.2%; 363/119230=3.0 1000 PY<br>27-30; n=264; 264/2281=11.6%; 264/51644=5.1 1000 PY<br>>30; n=97; 97/525=18.5%; 97/10353=9.4 1000 PY
Ohlsson, C.	2019	Sweden	1983.0	1945-1961 to 2013<br>28.3 y	n=36176<br>20 y; Female: 0%	Measured directly	Medical records	<25, n=620+32=652, 652/(32202+1309)=1.95%, 1.95/2.83=0.69 1000 PY<br>>25, n=152+82=234, 234/(1718+947)=8.78%, 8.78/2.83=3.10 1000 PY
Papier, K.	2017	Thailand	2008.0	2005 to 2009-2013<br>8 y	n=39021<br>Mean 35 y; Female: 55%	Self-report	Self-report	Total<br><18.5; n= 8; 8/5183=0.15%, 0.15/0.8=0.19 1000 PY<br>18.5-23.0; n=123; 123/20997=0.59%, 0.59/0.8=0.74 1000 PY<br>23.0-25.0; n=121; 121/6381=1.9%, 1.9/0.8=2.4 1000 PY<br>25.0-30.0; n=294; 294/5489=5.4%, 5.4/0.8=6.75 1000 PY<br>>30.0; n=142; 142/971=14.6%, 14.6/0.8=18.25 1000 PY<br>Male<br><18.5; n= 5, 5/962=0.5%, 0.5/0.8=0.63 1000 PY<br>18.5-23.0; n=67,67/8208=0.8%, 0.8/0.8=1 1000 PY<br>23.0-25.0; n=76, 76/4034=1.9%, 1.9/0.8=2.4 1000 PY<br>25.0-30.0; n=196, 196/3631=5.4%, 5.4/0.8=6.75 1000 PY<br>>30.0; n=86, 86/520=16.5%, 16.5/0.8=20.63 1000 PY<br>Female<br><18.5; n= 3, 3/4221=0.1%, 0.1/0.8=0.13 1000 PY

								18.5-23.0; n=56, 56/12789=0.4%, 0.4/0.8=0.5 1000 PY<br>23.0-25.0; n=45, 45/ 2347=1.9%, 1.9/0.8=2 1000 PY<br>25.0-30.0; n=98, 98/ 1858=5.3%, 5.3/0.8=6.63 1000 PY<br>>30.0; n=56, 56/ 451=12.4%, 12.4/0.8=15.5 1000 PY
Sairenchi, T.	2007	Japan	1998.5	1993 to 2004<br>Mean of 5.3 y	n=127213<br>40-79 y; Female: 69%	Measured directly	Biology test	Male<br>40-59 y<br><18.5, n= 17.9*1.176=21, 21/1176= 17.9 1000 PY<br>18.5–24.9, 14.2*46.547=661, 661/46547=14.2 1000 PY<br>25.0–29.9, 22.2*20.70=460, 460/20701=122.2 1000 PY<br>>30.0, 33.3*1.230=41, 41/1230=33.3 1000 PY<br>60-79 y<br><18.5, n=26.4*5.906=156, 156/5906=26.4 1000 PY<br>18.5–24.9, n=20.2*87.462=1767, 1767/87462=20.2 1000 PY<br>25.0–29.9, n=26.2*27.348=716, 716/27348=26.2 1000 PY<br>>30.0, n=31.8*1.353=43, 43/1353=31.8 1000 PY<br>Female<br>40-59 y<br><18.5, n=4.6*8.538=39, 39/8538=4.6 1000 PY<br>18.5–24.9, n=5.9*185.911=1097, 1097/185911=5.9 1000 PY<br>25.0–29.9, n=11.3*61.582=696, 696/61582=11.3 1000 PY<br>>30.0, n=31.5*5.838=184, 184/5838=31.5 1000 PY<br><18.5, n=11.8*8.953=106, 106/8953=11.8 1000 PY<br>18.5–24.9, 9.8*137.095=1343, 1343/137095=9.8 1000 PY<br>25.0–29.9, 15.1*64.294=971, 971/64294=15.1 1000 PY<br>>30.0, 26.2*5.722=150, 150/5722=26.2 1000 PY
Sasai, H.	2010	Japan	1999.0	1993 to 2006<br>Mean 5.5 y	n=61415<br>Mean 59 y; Female: 68%	Measured directly	Biology test	Male<br>40-59 y<br><25.0; n=396, 396/4914=8.1%, 396/26569=14.9 1000 PY<br>25.0-29.9; n=298, 298/2268=13.1%, 298/11791=25.3 1000 PY<br>>30; n=21, 21/147=14.3%, 21/718=29.2 1000 PY<br>**60-79 y**<br><25.0; n=968, 968/9560=10.1%, 968/47223=20.5 1000 PY<br>25.0-29.9; n=361, 361/2895=12.5%, 361/14029=25.7 1000 PY<br>>30; n=21, 21/142=14.8%, 21/619=33.9 1000 PY<br>Female<br>40-59 y<br><25.0; n=633, 633/15878=4.0%, 633/100461=6.3 1000 PY

								25.0-29.9; n=370, 370/5441=6.8%, 370/32439=11.4 1000 PY<br>>30; n=106, 106/640=16.6%, 106/3096=34.2 1000 PY<br>**60-79 y**<br><25.0; n=694, 694/12934=5.4%, 694/68069=10.2 1000 PY<br>25.0-29.9; n=478, 478/6002=8.0%, 478/30041=15.9 1000 PY<br>>30; n=77, 77/594=13.0%, 77/2511=30.7 1000 PY
Schmidt, M.	2013	Denmark	1993.5		n=6502<br>age=22 years<br>Male population	Measured directly	Medical records	<18.5, n=7; 7/353=2%; 0.63 1000 PY<br>18.5–24.9, n=207; 207/5407=3.8%; 1.22 1000 PY<br>25.0–29.9, n=76; 76/639=11.9%; 3.87 1000 PY<br>>30, n=26; 26/97=26.8; 9.46 1000 PY
Sheikh, M. A.	2014	Norway	2001.5	1998 to 2005<br>7 y	n=31302<br>Mean 47.7 y;<br>Female: 100%	Self-report	Self-report	<25.0; n=55; 55/21506=0.3%, 0.3/0.7=0.43 1000 PY<br>25.0-29.9; n=126; 126/9042=1.4%, 1.4/0.7=2 1000 PY<br>>30; n=132; 132/2619=5.0%, 5/0.7=7.14 1000 PY
Siegel, L. C.	2009	America	1994.0	1982 to 2006<br>Median 23.1 y	n=20757<br>40-84 y; Female: 0%	Self-report	Self-report	18.5-25.0; n=414+197=611; 611/12003=5.1%; 611/252466=2.4 1000 PY<br>25.0-29.9; n=623+363=986; 986/7914=12.5%; 986/162701=6.1 1000 PY<br>>30; n=140+99=239; 239/840=28.5%; 239/15959=15.0 1000 PY
Song, B. M.	2018	South Korea	2011.0	2005-2011 to 2014<br>Median 4.5 y	n=6484<br>≥40 y; Female: 62%	Measured directly	Multiple	<22.4; n=31; 1.9%, 1.9/0.45=4.2 1000 PY<br>22.4-24.2; n=57; 3.5%, 3.5/0.45=7.8 1000 PY<br>24.2-26.4; n=86; 5.3%, 5.3/0.45=11.8 1000 PY<br>>26.4; n=130; 8.0%, 8.0/0.45=17.8 1000 PY
Sui, X.	2008	America	1987.5	1971 to 2004<br>17 y	n=6249<br>Mean 43.8 y;<br>Female: 100%	Measured directly	Biology test	<25; n=87; 87/5077=1.7%; 87/92249=0.9 1000 PY<br>25-29.9; n=36; 36/875=4.1%; 36/12915=2.8 1000 PY<br>>30; n=20; 30/297-=10.1%; 20/4158=4.8 1000 PY
Sun, J.	2019	China	2010.5	2006-2009 to 2012-2015<br>Median 4.1 y	n=3033<br>35-74 y; Female: 64%	Measured directly	Biology test	Urban<br>18.5-23.9; n=277*6.3%=17; 6.3%; 6.3/0.41=15.0 1000 PY<br>>24; n=463*14.1%=65; 14.1%; 14.1/0.41=34.3 1000 PY<br>Rural<br>18.5-23.9; n=907*7.2%=65; 7.2%; 7.2/0.41=17.5 1000 PY<br>>24; n=1387*9.1%=126; 9.1%; 9.1/0.41=22.2 1000 PY
Tatsumi, Y.	2012	Japan	1998.5	1990-1992 to 2006<br>Mean 9.5 y	n=7240<br>40-79 y; Female: 64%	Measured directly	Multiple	Male<br><18.5, n=13; 16.1 1000 PY<br>18.5-22.9, n=106; 10.0 1000 PY<br>23-24.9, n=77, 13.9 1000 PY<br>≥25, n=100, 18.3 1000 PY<br>Female<br><18.5, n=20; 11.2 1000 PY

								18.5-22.9, n=121; 6.1 1000 PY<br>23-24.9, n=98; 8.2 1000 PY<br>≥25, n=180; 14.4 1000 PY
Twig, G.	2014	NR	Israel	1995 to 2011<br>Mean 6.1 y	n=33939<br>Mean 30.9 y; Female: 0%	Measured directly	Biology test	<25, n=65+66+28+14=173; 173/(56184+33008+11021+2704)=1.7 1000 PY<br>25-30, n=56+88+92+79=315; 315/(26605+28673+18538+7739)=3.9 1000 PY<br>>30, n=17+52+73+104=246; 246/(3909+7934+8541+5423)=9.5 1000 PY
Uemura, M.	2015	Japan	2006.5	2002 to 2011<br>8.9 y	n=4631<br>35-66 y; Female: 22%	Measured directly	Self-report	<25; n=148+28=176; 176/3630=4.8%, 4.8/0.89=5.4 1000 PY<br>>25; n=91+18=109; 109/1011=10.8%, 10.8/0.89=12.1 1000 PY
Vaidya, A.	2016	China	2008.0	2006 to 2010<br>4 y	n=90790<br>Mean 51y; Female: 20%	Measured directly	Self-report	Male<br><20, n=95; 6.33 1000 PY<br>20-22.9, n=577; 9.77 1000 PY<br>23-24.9, n=779; 12.4 1000 PY<br>25-27.9, n=1435; 17.1 1000 PY<br>28-29.9, n=651; 22.5 1000 PY<br>≥30, n=552; 28.1 1000 PY<br>Female<br><20, n=14; 1.89 1000 PY<br>20-22.9, n=107; 4.82 1000 PY<br>23-24.9, n=151; 8.23 1000 PY<br>25-27.9, n=211; 11.7 1000 PY<br>28-29.9, n=108; 17.1 1000 PY<br>≥30, n=161; 27.1 1000 PY
Villegas, R.	2009	China	1999.8	1997-2000 to 2002-2004<br>Mean 4.6 y	n=64191<br>40-70 y; Female: 100%	Measured directly	Multiple	<23, n=238; 238/129689=1.8 1000 PY, 1.8*0.46=0.83%<br>23-27.5, n=748; 748/129991=5.7 1000 PY, 4.7*0.46=2.2%<br>>27.5, n=614; 614/37589=16.3 1000 PY, 16.3*0.46=7.5%
Wang, B.	2017	China	2010.5	2007-2008 to 2013-2014<br>6 y	n=11865<br>Mean 47 y; Female: 62%	Measured directly	Biology test	18.5-23.9, n=193; 193/5689=3.4%; 193/34141.75=5.6 1000 PY<br>>24, n=529; 529/6176=8.6%; 529/36757.33=14.4 1000 PY
Wang, H.	2010	America	1996.0	1989-1991 to 2002<br>Median 7.8 y	n=1677<br>45-74 y; Female: 57%	Measured directly	Biology test	<25, n=23; 18.3%; 30.5 1000 PY<br>25-30, n=103; 26.5%; 45.4 1000 PY<br>>30, n=267; 47.1%; 91.4 1000 PY
Wang, Y.	2005	America	1993.0	1986 to 2000<br>13 y	n=27270<br>40–75 y; Female: 0%	Self-report	Self-report	14.2-22.7, n=58; 58/5398=1.1%; 58/70665=0.8 1000 PY<br>22.8-24.1, n=64; 64/5413=1.2%; 64/71424=0.9 1000 PY<br>24.2-25.3, n=111; 111/5416=2.0%; 111/70821=1.6 1000 PY<br>25.4-27.1, n=178; 178/5444=3.3%; 178/70666=2.5 1000 PY<br>27.2-54.2, n=473; 473/5599=8.4%; 473/70605=6.7 1000 PY

Wannamethee, S. G.	2005	UK	1989.5	1978-1980 to 2000<br>Mean 21.3 y	n=6910<br>40-59 y; Female: 0%	Self-report	Self-report	<25, n=95; 95/3313=2.9%; 1.6 1000 PY<br>25-27.5, n=135; 135/2192=6.2%; 3.5 1000 PY<br>27.5-29.9, n=114; 114/1104=10.3%; 5.9 1000 PY<br>>30, n=105; 105/567=18.5%; 11.4 1000 PY
Wei, Y.	2020	China	2011.0	2008-2010 to 2013<br>4.6 y	n=17801<br>Mean 63.2 y; Female: 52%	Measured directly	Biology test	18.5-23.9; n=151+248=399; 399/8176=4.9%, 4.9/0.46=10.65 1000 PY<br>24-27.9; n=146+562=708; 708/7186=9.8%, 9.8/0.46=21.30 1000 PY<br>>28; n=43+303=346; 346/2439=14.2%, 14.2/0.46=30.87 1000 PY
Weinstein, A. R.	2004	America	1995.0	1992 to 1998<br>6.9 y	n=37878<br>Mean 54.5 y; Female: 100%	Self-report	Self-report	<25, n=178, 0.9%,, 0.9/0.69=1.3 1000 PY<br>25-30, n=421, 3.6%, 3.6/0.69=5.2 1000 PY<br>>30, n=762, 11.6%, 11.6/ 0.69=16.8 1000 PY
Will, J. C.	2002	America	1965.8	1959-1960 to 1972<br>13 y	n=212570<br>>30 y; Female: 52.3%	Self-report	Self-report	Male<br>>25, n=3857+523+287+30+812=5509, 5509/(679205+73036+44591+4931+120050)=6.0 1000 PY<br>Female<br>>25, n=4290+870+343+66+1368=6937, 6937/(661558+98492+55555+7854+220376)=6.6 1000 PY
Williams, P. T.	2007	America	1996.5	1991-1994 to 1999-2002<br>7.8 y	n=41124<br>45 y for Male and 40 for Female; Female: 29%	Self-report	Self-report	Male<br><22.5, n=15, 0.2 1000 PY<br>22.5-25, n=44, 0.5 1000 PY<br>25-27.5, n=62, 1.3 1000 PY<br>>27.5, n=76, 4.1 1000 PY<br>Female<br><22.5, n=10, 0.1 1000 PY<br>22.5-25, n=5, 0.3 1000 PY<br>25-27.5, n=7, 1.2 1000 PY<br>>27.5, n=6, 2.3 1000 PY
Xia, M. F.	2018	China	2013.0	2009-2012 to 2014-2017<br>4.4 y	n=2558<br>55-70 y; Female: 61%	Measured directly	Medical records	<28, n=17; 17/212=8.0%, 8.0/0.44=18.2 1000 PY<br>>28, n=82; 82/2346=3.5%, 3.5/0.44=8.0 1000 PY
Ye, M.	2018	Canada	2009.5	2000-2008 to 2015<br>Mean 10.4 y	n=19164<br>Mean 50 y; Female: 62%	Self-report	Medical records	<25, n=61, 61/5049=1.2%; 61/53117.5=1.1 1000PY<br>25-29.9, n=305; 305/7063=4.3%; 305/72786.5=4.2 1000 PY<br>>30, n=642; 642/5391=11.9%; 642/52625.2=12.2 1000 PY
Andre, P.	2020	UK	2012.5	2007-2010 to 2014-2017<br>Mean 6.1 y	n=21585<br>Mean 56.5 y; Female 52%	Self-report	Self-report	<25: n=68; 68/9079=0.75%; 0.75/0.61=1.2 1000 PY<br>>25: n=405; 405/12033=3.37%; 3.67/0.61=6.0 1000 PY
Bardenheier, B. H.	2021	America	2009	2006 to 2010-2014<br>Mean 4.7 y	n=3313<br>Mean 54 y; Female: 53.3%	Measured directly	Biology test	18.5-25: n=0.5%*1122=6; 0.5%; 0.5/0.47=1.1 1000 PY<br>25-30: n=1.4%*1288=18; 1.4%; 1.4/0.47=3.0 1000 PY

								>30: n=3%*773=23; 3.0%; 3.0/0.47=6.4 1000 PY
Chen, Y.	2021	China	2012.5	2006-2008 to 2018<br>Mean 12 y	n=1199<br>Mean 46.6 years:<br>Females 56.2%	Measured directly	Multiple	<24: n=59; 5.35 1000 PY<br>>24: n=39; 11.62 1000 PY
Cuthbertson, D. J.	2021	UK	2006	2001 to 2011<br>10 y	n=2020<br>Mean 31.8 y,<br>Females: 56%	Measured directly	Multiple	<25: n=19; 19/1310=1.45%; 1.45/1=1.45 1000 PY<br>>=25; n=33; 33/710=4.65%; 4.65/1=4.65 1000 PY
Hodge, A. M.	2021	Australia	1998.5	1990-1994 to 1995-1998 and 2003-2007<br>13 y	n=25888<br>Mean 55.2 y;<br>Females 59.7%	Measured directly	Self-report	<25: n=133; 133/14461=0.92%; 0.92/1.3=0.7 1000 PY<br>25-30: n=541; 541/16914=3.2%; 3.2/1.3=2.5 1000 PY<br>>30: n=; 575/7810=7.36%; 7.36%/1.3=5.7 1000PY
Narayan, K. M. V.	2021	India and Pakistan	CARRS cohort: 2013.5<br><br>ARIC cohort: 1998	CARRS cohort: 2010-2011 to 2016-2017,<br>Mean 4.8 y<br><br>ARIC cohort: from 1989 to 2007<br>Mean 6.7 y	n=6676 for CARRS cohort<br>Median 33.0 y;<br>Females: 56.2%<br><br>n= 1852 for ARIC cohort<br>Median 27.4 y;<br>Females: 61.5%	Measured directly	Biology test	CARRS cohort<br>Male<br><25: n=68; 68/6622=10.3 1000 PY<br>25-30: n=62; 62/2846=21.8 1000 PY<br>>30: n=36; 36/752=47.9 1000 PY<br>Female<br><25: n=52; 52/6774=7.7 1000 PY<br>25-30: n=91; 91/4705=19.3 1000 PY<br>>30: n=98; 98/2610=37.5 1000 PY<br><br>ARIC cohort<br>Male<br><25: n=1; 1/503=2.0 1000 PY<br>25-30: n=21; 21/1314=16.0 1000 PY<br>>30: n=154; 154/3159=48.7 1000 PY<br>Female<br><25: n=5; 5/591=8.5 1000 PY<br>25-30: n=31; 31/1453=21.3 1000 PY<br>>30: n=298; 298/6076=49.0 1000 PY
Tang, M. L.	2021	China	2016.5	2014 to 2019<br>5 y	n=6911<br>Mean age 71 y;<br>Female 40.5%	Measured directly	Biology test	<18.5: n=5; 5/201=2.5%; 2.5/0.5=5.0 1000 PY<br>18.5-22.5: n=58; 58/1750=3.3%; 3.3/0.5=6.6 1000 PY<br>22.5-25: n=130; 130/2130=6.1%; 6.1/0.5=12.2 1000 PY<br>25-27.5: n=107; 107/1778=6.0%; 6/0.5=12.0 1000 PY<br>27.5-30: n=68; 68/777=8.8%; 8.8/0.5=17.5 1000 PY<br>>=30: n=26; 26/275=9.5%; 9.5/0.5=18.9 1000 PY
Xi, Y.	2021	China	2003	2001 to 2005<br>Mean 4.4 y	n=31197<br>Mean 33.0 y<br>Females 42.4%	Self-report	Self-report	<18.5: n=4; 4/2655=0.15%; 0.15/0.44=0.34 1000 PY<br>18.2-24: n=88; 88/20643=0.43%; 0.43/0.44=0.97 1000 PY<br>>24: n=136; 136/7899=1.72; 1.72/0.44=3.91 1000 PY
Xu, S.	2021	China	2012	2007-2008 to 2006-2017	n=1128<br>Mean 43.9 y	Measured directly	Biology test	<24: n=27+12=39; 39/(527+118)=6.05%; 6.05/0.9=6.72 1000 PY

				9 y	Females 57.7%			>=24: n=2+72=74; 74/(118+419)=13.78%; 13.78/0.9=15.31 1000 PY
Zhu, X.	2021	China	2018.5	2017 to 2020<br>Mean 1.64 y	n=3641<br>Mean 51 y<br>Females 62.2%	Measured directly	Biology test	18.5-24: n=90+35=125; 125/(1688+414)=59.5 1000 PY<br>24-28: n=58+113=171; 171/(1388+1317)=63.2 1000 PY<br>>28; n=16+103=119; 119/(322+853)=101.3 1000 PY

[a]Median year of data collection was calculated as the mean value of the follow-up year range;
[b]Multiple method was defined as blood test plus other ascertainment methods.

Table S4. Leave-one-out analysis for the pooled incidence of diabetes by baseline weight status

**Underweight (n=12 articles)**			**Normal weight (n=64 articles)**			**Overweight/obesity (n=81 articles)**		
Omitting study	Incidence (per 1000 person-years)	I^2	Omitting study	Incidence (per 1000 person-years)	I^2	Omitting study	Incidence (per 1000 person-years)	I^2
Pooled estimate	4.5 (2.8-7.3)	98.7%	Pooled estimate	2.7 (2.2-3.3)	99.5%	Pooled estimate	10.5 (9.3-11.8)	99.7%
Omitting Papier, K. (F)	5.2 (3.2-8.4)	98.7%	Omitting Williams, P. T. (F)	2.8 (2.3-3.4)	99.6%	Omitting Hu, H.	10.7 (9.6-11.9)	99.7%
Omitting Xi, Y.	5.1 (3.1-8.2)	98.7%	Omitting Fox, C. S.	2.7 (2.2-3.3)	99.6%	Omitting Williams, P. T. (F)	10.6 (9.5-12.0)	99.7%
Omitting Papier, K. (M)	4.9 (3.0-8.0)	98.7%	Omitting Field, A. E.	2.7 (2.2-3.4)	99.6%	Omitting Bardenheier, B. H.	10.7 (9.5-12.0)	99.7%
Omitting Schmidt, M.	5.0 (3.1-8.0)	98.7%	Omitting Williams, P. T. (M)	2.7 (2.2-3.4)	99.5%	Omitting Chang, Y.	10.6 (9.5-12.0)	99.7%
Omitting Chang, Y.	4.9 (3.0-8.0)	98.7%	Omitting Sheikh, M. A.	2.7 (2.2-3.4)	99.5%	Omitting Williams, P. T. (M)	10.6 (9.4-12.0)	99.7%
Omitting Bragg, F. (F)	4.9 (3.0-7.8)	98.6%	Omitting Hu, H.	2.7 (2.3-3.3)	99.4%	Omitting Ohlsson, C.	10.6 (9.4-12.0)	99.7%
Omitting Bragg, F. (M)	4.9 (3.1-7.8)	98.4%	Omitting Papier, K. (F)	2.7 (2.2-3.4)	99.5%	Omitting Sheikh, M. A.	10.6 (9.4-12.0)	99.7%
Omitting Hara, H.	4.6 (2.9-7.5)	98.8%	Omitting Carey, V. J.	2.7 (2.2-3.3)	99.5%	Omitting Sui, X.	10.6 (9.4-12.0)	99.7%
Omitting Jung, H. H.	4.6 (2.6-7.9)	98.7%	Omitting Hu, F. B.	2.7 (2.2-3.3)	99.5%	Omitting Holtermann, A.	10.6 (9.4-11.9)	99.7%
Omitting de Mutsert, R.	4.5 (2.7-7.6)	98.8%	Omitting Ohlsson, C.	2.7 (2.2-3.3)	99.5%	Omitting Chan, J. M.	10.6 (9.4-12.0)	99.7%
Omitting Tang, M. L.	4.5 (2.8-7.4)	98.8%	Omitting Hodge, A. M.	2.7 (2.2-3.3)	99.5%	Omitting Hu, G.	10.6 (9.4-11.9)	99.7%
Omitting Fujita, M. (F, Chiba)	4.5 (2.7-7.4)	98.8%	Omitting Feller, S. (F)	2.7 (2.2-3.3)	99.6%	Omitting Mishra, G. D.	10.6 (9.4-11.9)	99.7%
Omitting Sairenchi, T. (F)	4.4 (2.6-7.3)	98.7%	Omitting Chan, J. M.	2.7 (2.2-3.3)	99.6%	Omitting Maty, S. C.	10.6 (9.4-11.9)	99.7%
Omitting Fujita, M. (F, Kashiwa)	4.4 (2.7-7.2)	98.7%	Omitting Bardenheier, B. H.	2.7 (2.2-3.3)	99.6%	Omitting Xi, Y.	10.6 (9.4-11.9)	99.7%
Omitting Tatsumi, Y. (F)	4.4 (2.7-7.1)	98.7%	Omitting Hu, G.	2.7 (2.2-3.3)	99.6%	Omitting Oguma, Y.	10.6 (9.4-11.9)	99.7%
Omitting Asghar, S. (F)	4.3 (2.7-7.0)	98.7%	Omitting Sui, X.	2.7 (2.2-3.3)	99.6%	Omitting Fox, C. S.	10.6 (9.4-11.9)	99.7%
Omitting Asghar, S. (M)	4.3 (2.6-7.0)	98.7%	Omitting Xi, Y.	2.7 (2.2-3.3)	99.6%	Omitting Hodge, A. M.	10.6 (9.4-11.9)	99.7%
Omitting Tatsumi, Y. (M)	4.3 (2.6-7.0)	98.7%	Omitting Dotevall, A.	2.7 (2.2-3.3)	99.6%	Omitting Schmidt, M.	10.6 (9.4-11.9)	99.7%
Omitting Fujita, M. (M, Chiba)	4.3 (2.6-6.9)	98.7%	Omitting Mishra, G. D.	2.7 (2.2-3.3)	99.6%	Omitting Wang, Y.	10.6 (9.4-11.9)	99.7%
Omitting Jung, J. Y. (F)	4.2 (2.6-6.9)	98.7%	Omitting Papier, K. (M)	2.7 (2.2-3.3)	99.6%	Omitting Cuthbertson, D. J.	10.6 (9.4-11.9)	99.7%
Omitting Sairenchi, T. (M)	4.2 (2.8-6.5)	98.1%	Omitting Wang, Y.	2.7 (2.2-3.3)	99.5%	Omitting de Mutsert, R.	10.6 (9.4-11.9)	99.7%
			Omitting Ye, M.	2.7 (2.2-3.3)	99.6%	Omitting Carey, V. J.	10.6 (9.4-11.9)	99.7%
			Omitting Dow, C.	2.7 (2.2-3.3)	99.5%	Omitting Dow, C.	10.6 (9.4-11.9)	99.7%
			Omitting Schmidt, M.	2.7 (2.2-3.3)	99.5%	Omitting Hu, F. B.	10.6 (9.4-11.9)	99.7%
			Omitting Andre, P.	2.7 (2.2-3.3)	99.6%	Omitting Dotevall, A.	10.6 (9.4-11.9)	99.7%

			Omitting Holtermann, A.	2.7 (2.2-3.3)	99.6%	Omitting Papier, K. (F)	10.6 (9.4-11.9)	99.7%
			Omitting Weinstein, A. R.	2.7 (2.2-3.3)	99.5%	Omitting Twig, G.	10.6 (9.4-11.9)	99.7%
			Omitting Chang, Y.	2.7 (2.2-3.3)	99.5%	Omitting Nanri, A.	10.6 (9.4-11.9)	99.7%
			Omitting Cuthbertson, D. J.	2.7 (2.2-3.3)	99.6%	Omitting Papier, K. (M)	10.6 (9.4-11.9)	99.7%
			Omitting Wannamethee, S. G.	2.7 (2.2-3.3)	99.6%	Omitting Andre, P.	10.6 (9.4-11.9)	99.7%
			Omitting Oguma, Y.	2.7 (2.2-3.3)	99.5%	Omitting Wannamethee, S. G.	10.6 (9.4-11.9)	99.7%
			Omitting Twig, G.	2.7 (2.2-3.3)	99.6%	Omitting Bragg, F. (M)	10.6 (9.4-11.9)	99.7%
			Omitting Maty, S. C.	2.7 (2.2-3.3)	99.6%	Omitting Bragg, F. (F)	10.6 (9.4-11.9)	99.7%
			Omitting Meisinger, C. (F)	2.7 (2.2-3.3)	99.6%	Omitting Will, J. C. (M)	10.6 (9.4-11.9)	99.7%
			Omitting Villegas, R.	2.7 (2.2-3.3)	99.6%	Omitting Feng, S.	10.6 (9.4-11.9)	99.7%
			Omitting Beleigoli, A. M.	2.7 (2.2-3.3)	99.6%	Omitting Feller, S. (F)	10.5 (9.4-11.9)	99.7%
			Omitting Feller, S. (M)	2.7 (2.2-3.3)	99.6%	Omitting Manson, J. E.	10.5 (9.4-11.9)	99.7%
			Omitting Arnlov, J.	2.7 (2.2-3.3)	99.6%	Omitting Will, J. C. (F)	10.5 (9.3-11.9)	99.7%
			Omitting Meigs, J. B.	2.7 (2.2-3.3)	99.6%	Omitting Siegel, L. C.	10.5 (9.4-11.9)	99.7%
			Omitting Siegel, L. C.	2.7 (2.2-3.3)	99.6%	Omitting Field, A. E.	10.5 (9.4-11.9)	99.7%
			Omitting de Mutsert, R.	2.7 (2.2-3.3)	99.6%	Omitting Appleton, S. L.	10.5 (9.3-11.9)	99.7%
			Omitting Appleton, S. L.	2.7 (2.2-3.3)	99.6%	Omitting Feller, S. (M)	10.5 (9.3-11.9)	99.7%
			Omitting Krishnan, S.	2.7 (2.2-3.3)	99.6%	Omitting Ye, M.	10.5 (9.3-11.9)	99.7%
			Omitting Ford, E. S.	2.7 (2.2-3.3)	99.6%	Omitting Beleigoli, A. M.	10.5 (9.3-11.9)	99.7%
			Omitting Li, F.	2.7 (2.2-3.3)	99.6%	Omitting Arnlov, J.	10.5 (9.3-11.9)	99.7%
			Omitting Meisinger, C. (M)	2.7 (2.2-3.3)	99.6%	Omitting Xia, M. F.	10.5 (9.3-11.9)	99.7%
			Omitting Nguyen, B.	2.7 (2.2-3.3)	99.6%	Omitting Villegas, R.	10.5 (9.3-11.9)	99.7%
			Omitting Aung, K.	2.7 (2.2-3.3)	99.6%	Omitting Weinstein, A. R.	10.5 (9.3-11.9)	99.7%
			Omitting Jung, C. H.	2.7 (2.2-3.3)	99.6%	Omitting Meigs, J. B.	10.5 (9.3-11.8)	99.7%
			Omitting Borné, Y. (F)	2.7 (2.2-3.3)	99.6%	Omitting Borné, Y. (F)	10.5 (9.3-11.8)	99.7%
			Omitting Feng, S.	2.7 (2.2-3.3)	99.6%	Omitting Meisinger, C. (F)	10.5 (9.3-11.8)	99.7%
			Omitting Hayashi, T.	2.7 (2.2-3.3)	99.6%	Omitting Tang, M. L.	10.5 (9.3-11.8)	99.7%
			Omitting Song, B. M.	2.7 (2.2-3.3)	99.6%	Omitting Hu, G.	10.5 (9.3-11.8)	99.7%
			Omitting Vaidya, A. (F)	2.7 (2.2-3.3)	99.6%	Omitting Nguyen, B.	10.5 (9.3-11.8)	99.7%
			Omitting Lim, J. S.	2.7 (2.2-3.3)	99.6%	Omitting Ford, E. S.	10.5 (9.3-11.8)	99.7%
			Omitting Borné, Y. (M)	2.7 (2.2-3.3)	99.6%	Omitting Meisinger, C. (M)	10.5 (9.3-11.8)	99.7%

			Omitting Hadaegh, F.	2.7 (2.2-3.3)	99.6%	Omitting Jung, C. H.	10.5 (9.3-11.8)	99.7%
			Omitting Hinnouho, G. M.	2.7 (2.2-3.3)	99.6%	Omitting Hadaegh, F.	10.5 (9.3-11.8)	99.7%
			Omitting Tang, M. L.	2.7 (2.2-3.3)	99.6%	Omitting Tatsumi, Y. (F)	10.5 (9.3-11.8)	99.7%
			Omitting Jung, H. H.	2.7 (2.1-3.3)	99.4%	Omitting Hayashi, T.	10.5 (9.3-11.8)	99.7%
			Omitting Jiamjarasrangsi, W.	2.7 (2.2-3.3)	99.6%	Omitting Chen, Y.	10.5 (9.3-11.8)	99.7%
			Omitting Chen, Y.	2.7 (2.2-3.3)	99.6%	Omitting Lim, J. S.	10.5 (9.3-11.8)	99.7%
			Omitting Uemura, M.	2.7 (2.2-3.3)	99.6%	Omitting Uemura, M.	10.5 (9.3-11.8)	99.7%
			Omitting Han, C.	2.7 (2.2-3.3)	99.6%	Omitting Jae, S. Y.	10.5 (9.3-11.8)	99.7%
			Omitting Edwards, M. K.	2.7 (2.2-3.3)	99.6%	Omitting Song, B. M.	10.5 (9.3-11.8)	99.7%
			Omitting Wang, B.	2.7 (2.2-3.3)	99.6%	Omitting Li, F.	10.5 (9.3-11.8)	99.7%
			Omitting Tatsumi, Y. (F)	2.6 (2.2-3.3)	99.6%	Omitting Jung, H. H.	10.5 (9.2-12.0)	99.7%
			Omitting Xu, S.	2.6 (2.2-3.3)	99.6%	Omitting Borné, Y. (M)	10.5 (9.3-11.8)	99.7%
			Omitting Hara, H.	2.6 (2.2-3.2)	99.6%	Omitting Asvold, B. O.	10.5 (9.3-11.8)	99.7%
			Omitting Vaidya, A. (M)	2.6 (2.1-3.2)	99.5%	Omitting Hinnouho, G. M.	10.5 (9.3-11.8)	99.7%
			Omitting Tatsumi, Y. (M)	2.6 (2.1-3.2)	99.6%	Omitting Hara, H.	10.5 (9.3-11.8)	99.7%
			Omitting Wei, Y.	2.6 (2.1-3.2)	99.5%	Omitting Wang, B.	10.5 (9.3-11.8)	99.7%
			Omitting Ishikawa-Takata, K.	2.6 (2.1-3.2)	99.6%	Omitting Han, C.	10.5 (9.3-11.8)	99.7%
			Omitting Janghorbani, M.	2.6 (2.1-3.2)	99.6%	Omitting Sasai, H. (F)	10.5 (9.3-11.8)	99.7%
			Omitting Li, W. D.	2.6 (2.1-3.2)	99.5%	Omitting Xu, S.	10.5 (9.3-11.8)	99.7%
			Omitting Asghar, S. (F)	2.6 (2.1-3.2)	99.6%	Omitting Sairenchi, T. (F)	10.5 (9.3-11.8)	99.7%
			Omitting Jung, J. Y. (F)	2.6 (2.1-3.2)	99.5%	Omitting Vaidya, A. (F)	10.5 (9.3-11.8)	99.7%
			Omitting Sun, J. (Urban)	2.6 (2.1-3.2)	99.6%	Omitting Gautier, A. (F)	10.5 (9.3-11.8)	99.7%
			Omitting Asghar, S. (M)	2.6 (2.1-3.2)	99.6%	Omitting Gautier, A. (M)	10.5 (9.3-11.8)	99.7%
			Omitting Sun, J. (ARural)	2.6 (2.1-3.2)	99.5%	Omitting Tatsumi, Y. (M)	10.5 (9.3-11.8)	99.7%
			Omitting Jung, J. Y. (M)	2.6 (2.1-3.2)	99.5%	Omitting Edwards, M. K.	10.5 (9.3-11.8)	99.7%
			Omitting Wang, H.	2.6 (2.1-3.2)	99.6%	Omitting Fujita, M. (F, Chiba)	10.5 (9.3-11.8)	99.7%
			Omitting Zhu, X.	2.6 (2.1-3.1)	99.5%	Omitting Jiamjarasrangsi, W.	10.5 (9.3-11.8)	99.7%
						Omitting Aung, K.	10.4 (9.3-11.8)	99.7%
						Omitting Asghar, S. (F)	10.5 (9.3-11.8)	99.7%
						Omitting Vaidya, A. (M)	10.4 (9.3-11.8)	99.7%
						Omitting Janghorbani, M.	10.4 (9.3-11.8)	99.7%

						Omitting Krishnan, S.	10.4 (9.3-11.8)	99.7%
						Omitting Fujita, M. (F, Kashiwa)	10.4 (9.3-11.8)	99.7%
						Omitting Ishikawa-Takata, K.	10.4 (9.3-11.8)	99.7%
						Omitting Dowse, G. K.	10.4 (9.3-11.8)	99.7%
						Omitting Sun, J. (Rural)	10.4 (9.3-11.8)	99.7%
						Omitting Jung, J. Y. (F)	10.4 (9.3-11.7)	99.7%
						Omitting Wei, Y.	10.4 (9.3-11.7)	99.7%
						Omitting Sasai, H. (M)	10.4 (9.2-11.7)	99.7%
						Omitting Narayan, K. M. V. (F, CARRS)	10.4 (9.2-11.7)	99.7%
						Omitting Sairenchi, T. (M)	10.4 (9.2-11.7)	99.7%
						Omitting Jung, J. Y. (M)	10.4 (9.2-11.7)	99.7%
						Omitting Asghar, S. (M)	10.4 (9.3-11.7)	99.7%
						Omitting Fujita, M. (M, Chiba)	10.4 (9.2-11.7)	99.7%
						Omitting Narayan, K. M. V. (M, CARRS)	10.4 (9.2-11.7)	99.7%
						Omitting Nagayoshi, M.	10.4 (9.2-11.7)	99.7%
						Omitting Chan, J. C. Y. (F)	10.4 (9.2-11.7)	99.7%
						Omitting Chan, J. C. Y. (M)	10.4 (9.2-11.7)	99.7%
						Omitting Li, W. D.	10.4 (9.2-11.7)	99.7%
						Omitting Sun, J. (Urban)	10.4 (9.2-11.7)	99.7%
						Omitting Fujita, M. (M, Kashiwa)	10.4 (9.2-11.7)	99.7%
						Omitting Narayan, K. M. V. (M, ARIC)	10.4 (9.2-11.7)	99.7%
						Omitting Narayan, K. M. V. (F, ARIC)	10.4 (9.2-11.7)	99.7%
						Omitting Giraldez-Garcia, C.	10.3 (9.2-11.7)	99.7%
						Omitting Ning, F.	10.3 (9.2-11.6)	99.7%
						Omitting Wang, H.	10.3 (9.2-11.6)	99.7%
						Omitting Zhu, X.	10.3 (9.2-11.6)	99.7%

Table S5. The correlation matrix of modifiers that included for multivariate meta-regression models by baseline weight status

For underweight

	**Age, y**	**WHO Region**	World Bank country	**Weight assessment**	Diabetes ascertainment
Median year of data collection	0.08	0.32	**0.50****	0.09	-0.27
Age, y		-0.11	-0.27	-0.26	-0.13
WHO Region			-0.21	-0.46*	-0.52*
World Bank country				0.39	0.23
Weight assessment					**0.75*****

For normal weight

	**Sex**	**Age, y**	WHO Region	World Bank country	Follow-up duration, y	**Weight assessment**	Diabetes ascertainment
Median year of data collection	0.20	0.18	0.33**	**0.50*****	**-0.53****	-0.22*	-0.28*
Sex		0.07	-0.02	0.21*	-0.14	-0.30**	-0.23*
Age, y			-0.09	-0.04	-0.15	-0.08	0.01
WHO Region				0.13	0.03	**-0.50*****	-0.14
World Bank country					-0.36*	-0.15	-0.21*
Follow-up duration, y						0.17	0.19
Weight assessment							**0.57*****

For overweight/obesity

	**Age, y**	WHO Region	World Bank country	Follow-up duration, y	**Weight assessment**	Diabetes ascertainment
Median year of data collection	0.14	0.34***	**0.51*****	**-0.51*****	-0.35***	-0.36***
Age, y		0.01	-0.13	-0.14	-0.09	-0.03
WHO Region			0.08	0.01	**-0.51*****	-0.20*
World Bank country				-0.35***	-0.19*	-0.25*
Follow-up duration, y					0.19*	0.25**
Weight assessment						**0.60*****

**p*-value <0.05; ***p*-value <0.01; ****p*-value <0.001

Table S6. The characteristics of included studies reported prediabetes incidence

Author	Publication year	Country/region	Median year[a]	Follow-up (year range, duration)	Participants (n, age, female %)	Weight measurement	Diabetes measurement[b]	Incidence (n for cases; % for cumulative incidence; PY, person-years for incidence rate)
Sakurai, M.	2020	Japan	2015.5	2014 to 2017<br>3 y	n=11900<br>40.3 y; Female: 28%	Measured directly	Biology test	Hyperglycemia (prediabetes and diabetes)<br>Male<br><25, n=141+62+40+15=258, 258/(3221+1803+995+471)=4.0%; 4.0/0.3=13.3 1000 PY<br>>25, n=82+60+42+22=206, 206/(1002+581+343+157)=9.9%; 9.9/0.3=33.0 1000 PY<br>Female<br><25, n=43+18+6+2=69, 69/(1673+689+335+172)=2.4%; 2.4/0.3=8.0 1000 PY<br>>25, n=26+9+9+2=46, 46/(254+112+60+32)=10.0%; 10/0.3=33.3 1000 PY
Vaidya, A.	2016	China	2008.0	2006 to 2010<br>4 y	n=90790<br>Mean 51y;<br>Female: 20%	Measured directly	Self-report	Prediabetes<br>Male<br><20, n=694; 60.4 1000 PY<br>20-22.9, n=2974; 71.2 1000 PY<br>23-24.9, n=3353; 80.2 1000 PY<br>25-27.9, n=4592; 86.1 1000 PY<br>28-29.9, n=1572; 89.8 1000 PY<br>>=30, n=1120; 95.9 1000 PY<br>Female<br><20, n=218; 34.8 1000 PY<br>20-22.9, n=811; 45.3 1000 PY<br>23-24.9, n=809; 57.3 1000 PY<br>25-27.9, n=917; 71.9 1000 PY<br>28-29.9, n=1322; 76.3 1000 PY<br>>=30, n=323; 84.1 1000 PY
Wang, G.	2018	China	2002.3	1998-2000 to 2005-2006<br>6 y	n=1073<br>Mean 32 y;<br>Female: 48%	Measured directly	Biology test	Hyperglycemia (prediabetes and diabetes)<br><18.5, n=14, 14/85=16.5%, 16.5/0.6=27.5 1000 PY<br>18.5-23, n=220, 220/783=28.1%, 28.1/0.6=46.8 1000 PY<br>>23, n=59, 59/205=28.9%, 28.9/0.6=48.2 1000 PY
Xia, M. F.	2018	China	2013.0	2009-2012 to 2014-2017<br>4.4 y	n=2558<br>55-70 y;<br>Female: 61%	Measured directly	Medical records	Prediabetes<br><28, n=65; 65/212=30.7%, 30.7/0.44=69.8 1000 PY<br>>28, n=481; 481/2346=20.5%, 20.5/0.44=46.6 1000 PY

Cuthbertson, D. J.	2021	UK	2006	2001 to 2011<br>10 y	n=2020<br>Mean 31.8 y,<br>Females: 56%	Measured directly	Multiple	Prediabetes<br><25: n=339; 339/1310=25.88%; 25.88/1=25.9 1000 PY<br>>25; n=286; 286/710=40.28%; 40.28/1=40.3 1000 PY

[a]Median year of data collection was calculated as the mean value of the follow-up year range;
[b]Multiple method was defined as blood test plus other ascertainment methods.

Supplementary Figures legends

Figure S1. PRISMA flowchart

Note: Databases were searched from inception to November 13, 2020 and updated to December 8, 2021.

PRISMA: Preferred Reporting Items for Systematic Reviews and Meta-Analyses.

Figure S2. The pooled incidence of diabetes by baseline weight status after excluding studies with NOS <7

A: Underweight; **B**: Normal weight; **C**: Overweight/obesity

Note: Study quality was assessed by the Newcastle-Ottawa Scale (NOS); a score of NOS ≥7 was defined as good quality.

BMI: body mass index (kg/m^2); F: female; M: male

Figure S3. The bubble plot of diabetes incidence by baseline weight status

A: Underweight; the results of univariate meta-regression model indicated that the median year of data collection was not associated with the trends of diabetes incidence (β = -0.053, 95% CI: -0.116 to 0.010, *p*-value = 0.100, variance explained R^2 = 37.2%, Maximum likelihood test *p*-value = 0.100); the multivariate meta-regression model indicated that the median year of data collection was associated with the trends of diabetes incidence (β = -0.092, 95% CI: -0.173 to -0.011, *p*-value = 0.027, variance explained R^2 = 64.4%, Maximum likelihood test *p*-value<0.001) after the adjustment for age, follow-up duration, and weight assessment methods;

B: Normal weight; the results of univariate meta-regression model indicated that the median year of data collection was associated with the trends of diabetes incidence (β = 0.043, 95% CI: 0.024 to 0.063, *p*-value<0.001, variance explained R^2 = 16.96%); the multivariate meta-regression model indicated that the median year of data collection was also associated with the trends of diabetes incidence (β = 0.024, 95% CI: 0.003 to 0.046, *p*-value = 0.028, variance explained R^2 = 40.0%, Maximum likelihood test *p*-value<0.001) after the adjustment for age, sex, and weight assessment methods;

C: Overweight/obesity; the results of univariate meta-regression model indicated that the median year of data collection was associated with the trends of diabetes incidence (β = 0.028, 95% CI: 0.016 to 0.039 *p*-value<0.001, variance explained R^2 = 1.76%); the multivariate meta-regression model indicated that the median year of data collection was also associated with the trends of diabetes incidence (β = 0.016, 95% CI: 0.003 to 0.030, *p*-value = 0.019, variance explained R^2 = 25.1%, Maximum likelihood test *p*-value<0.001) after the adjustment for age and weight assessment methods.

Figure S4. The bubble plot of diabetes incidence by baseline weight status after excluding studies with NOS <7

A: Underweight; the results of univariate meta-regression model indicated that the median year of data collection was associated with the trends of diabetes incidence (β = -0.097, 95% CI: -0.166 to -0.0281, *p*-value = 0.006, variance explained R^2 = 53.42%); the multivariate meta-regression model indicated that the median year of data collection was also associated with the trends of diabetes incidence (β = -0.114, 95% CI: -0.190 to -0.039, *p*-value = 0.003, variance explained R^2 = 73.27%, Maximum likelihood test *p*-value<0.001) after the adjustment for age, WHO region, and weight assessment methods;

B: Normal weight; the results of univariate meta-regression model indicated that the median year of data collection was associated with the trends of diabetes incidence (β = 0.041, 95% CI: 0.017 to 0.066, *p*-value<0.001, variance explained R^2 = 10.23); the multivariate meta-regression model indicated that the median year of data collection was also associated with the trends of diabetes incidence (β = 0.034, 95% CI: 0.001 to 0.059, *p*-value = 0.001, variance explained R^2 = 24.49%, Maximum likelihood test *p*-value<0.001) after the adjustment for age, sex, and weight assessment methods;

C: Overweight/obesity; the results of univariate meta-regression model indicated that the median year of data collection was associated with the trends of diabetes incidence (β = 0.026, 95% CI: 0.010 to 0.043, *p*-value = 0.002, variance explained R^2<0.01%); the multivariate meta-regression model indicated that the median year of data collection was also associated with the trends of diabetes incidence (β = 0.019, 95% CI: 0.002 to 0.037, *p*-value = 0.029, variance explained R^2 = 20.75%, Maximum likelihood test *p*-value<0.001) after the adjustment for age and weight assessment methods.

Figure S5. The funnel plot for the pooled diabetes incidence by baseline weight status

A: Underweight; linear regression test for asymmetry: Test result: *t* = 0.54, df = 21, *p*-value = 0.593. The study of Hara, H had the lowest standard error (SE).

B: Normal weight; linear regression test for asymmetry: Test result: *t* = -2.07, df = 81, *p*-value = 0.042. The study of Fox, C. S. had the lowest SE.

C: Overweight/obesity; linear regression test for asymmetry: Test result: *t* = -0.81, df = 111, *p*-value = 0.421. The study of Williams, P. T, Hara, H, Asghar, S, and Fox, C. S. had the lowest SE.

Figure S1

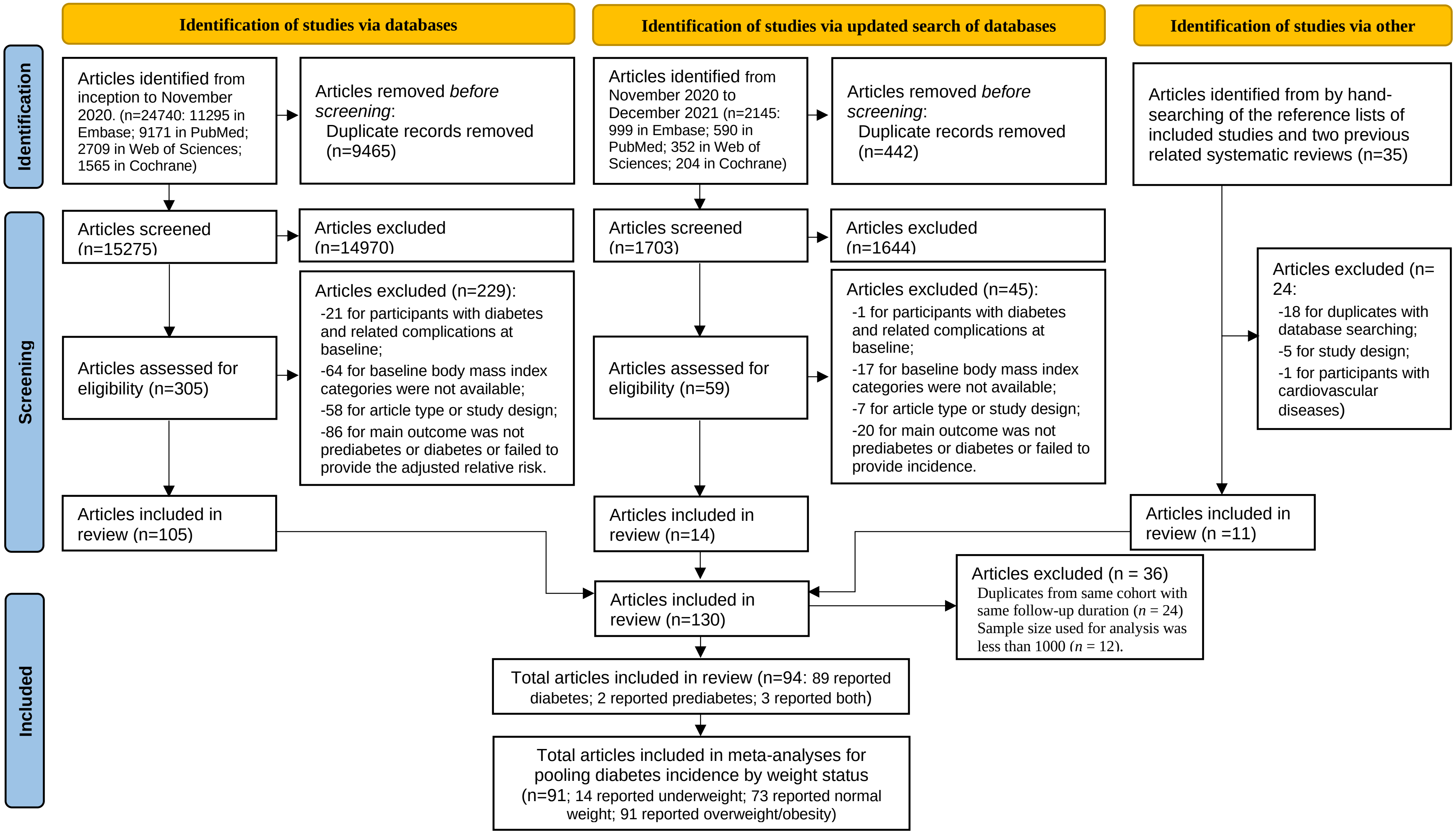

Identification of studies via databases
Identification of studies via updated search of databases
Identification of studies via other
Identification
Screening
Included
Articles identified from inception to November 2020. (n=24740: 11295 in Embase; 9171 in PubMed; 2709 in Web of Sciences; 1565 in Cochrane)
Articles removed *before screening*: Duplicate records removed (n=9465)
Articles identified from November 2020 to December 2021 (n=2145: 999 in Embase; 590 in PubMed; 352 in Web of Sciences; 204 in Cochrane)
Articles removed *before screening*: Duplicate records removed (n=442)
Articles identified from by hand-searching of the reference lists of included studies and two previous related systematic reviews (n=35)
Articles screened (n=15275)
Articles excluded (n=14970)
Articles screened (n=1703)
Articles excluded (n=1644)
Articles excluded (n=229):
-21 for participants with diabetes and related complications at baseline;
-64 for baseline body mass index categories were not available;
-58 for article type or study design;
-86 for main outcome was not prediabetes or diabetes or failed to provide the adjusted relative risk.
Articles assessed for eligibility (n=305)
Articles assessed for eligibility (n=59)
Articles excluded (n=45):
-1 for participants with diabetes and related complications at baseline;
-17 for baseline body mass index categories were not available;
-7 for article type or study design;
-20 for main outcome was not prediabetes or diabetes or failed to provide incidence.
Articles excluded (n= 24:
-18 for duplicates with database searching;
-5 for study design;
-1 for participants with cardiovascular diseases)
Articles included in review (n=105)
Articles included in review (n=14)
Articles included in review (n =11)
Articles included in review (n=130)
Articles excluded (n = 36)
Duplicates from same cohort with same follow-up duration (*n* = 24)
Sample size used for analysis was less than 1000 (*n* = 12).
Total articles included in review (n=94: 89 reported diabetes; 2 reported prediabetes; 3 reported both)
Total articles included in meta-analyses for pooling diabetes incidence by weight status (n=91; 14 reported underweight; 73 reported normal weight; 91 reported overweight/obesity)

Figure S2

A. Underweight

Study	Year	BMI	Cases	Personyears	Rate	95%-CI	Weight
Papier, K. (F)	2017	<18.5	3	23076.9	0.1	(0.0- 0.4)	4.2%
Papier, K. (M)	2017	<18.5	5	7936.5	0.6	(0.3- 1.5)	4.6%
Schmidt, M.	2013	<18.5	7	11111.1	0.6	(0.3- 1.3)	4.8%
Chang, Y.	2016	<18.5	20	25000.0	0.8	(0.5- 1.2)	5.1%
Bragg, F. (F)	2018	<18.5	104	94545.5	1.1	(0.9- 1.3)	5.2%
Bragg, F. (M)	2018	<18.5	182	151666.7	1.2	(1.0- 1.4)	5.2%
Jung, H. H.	2017	<18.5	513	160312.5	3.2	(2.9- 3.5)	5.2%
Tang, M. L.	2021	<18.5	5	1005.0	5.0	(2.1-12.0)	4.6%
Fujita, M. (F, Chiba)	2009	<18.5	83	15060.0	5.5	(4.4- 6.8)	5.2%
Sairenchi, T. (F)	2007	<18.5	145	17491.0	8.3	(7.0- 9.8)	5.2%
Fujita, M. (F, Kashiwa)	2009	<18.5	42	4608.0	9.1	(6.7-12.3)	5.2%
Tatsumi, Y. (F)	2012	<18.5	20	1785.7	11.2	(7.2-17.4)	5.1%
Asghar, S. (F)	2011	<18.5	23	1769.2	13.0	(8.6-19.6)	5.1%
Asghar, S. (M)	2011	<18.5	18	1125.0	16.0	(10.1-25.4)	5.0%
Tatsumi, Y. (M)	2012	<18.5	13	807.5	16.1	(9.3-27.7)	5.0%
Fujita, M. (M, Chiba)	2009	<18.5	45	2606.0	17.3	(12.9-23.1)	5.2%
Jung, J. Y. (F)	2018	<18.5	10	450.0	22.2	(12.0-41.3)	4.9%
Sairenchi, T. (M)	2007	<18.5	177	7082.0	25.0	(21.6-29.0)	5.2%
Fujita, M. (M, Kashiwa)	2009	<18.5	25	960.0	26.0	(17.6-38.5)	5.1%
Jung, J. Y. (M)	2018	<18.5	18	627.0	28.7	(18.1-45.6)	5.0%
**Random effects model**					**5.2**	**(3.1- 8.8)**	**100.0%**

Heterogeneity: $I^2 = 98.9\%$, $\tau = 1.1697$, $p < 0.001$

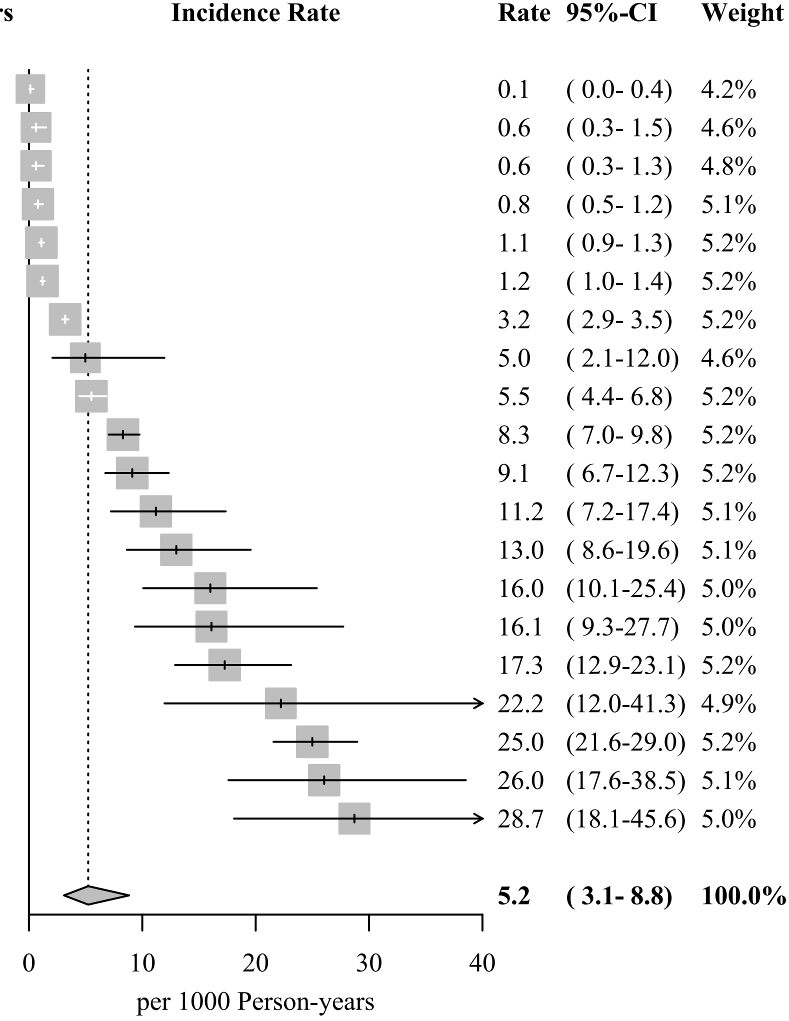

Figure S2

B. Normal weight

Study	Year	BMI	Cases	Personyears	Rate	95%-CI	Weight
Fox, C. S.	2006	<25.0	1	4000.0	0.2	(0.0- 1.8)	0.8%
Hu, H.	2017	<23.0	964	1818867.9	0.5	(0.5- 0.6)	1.6%
Papier, K. (F)	2017	18.5-23.0	56	102312.0	0.5	(0.4- 0.7)	1.6%
Ohlsson, C.	2019	<25.0	652	944927.5	0.7	(0.6- 0.7)	1.6%
Hodge, A. M.	2021	<25.0	133	187323.9	0.7	(0.6- 0.8)	1.6%
Feller, S. (F)	2010	<25.0	48	64000.0	0.8	(0.6- 1.0)	1.6%
Bardenheier, B. H.	2021	18.5-25.0	6	7792.2	0.8	(0.3- 1.7)	1.4%
Hu, G.	2004	<25.0	11	12222.2	0.9	(0.5- 1.6)	1.5%
Sui, X.	2008	<25.0	87	96666.7	0.9	(0.7- 1.1)	1.6%
Dotevall, A.	2004	<24.0	13	13090.6	1.0	(0.6- 1.7)	1.5%
Papier, K. (M)	2017	18.5-23.0	67	65664.0	1.0	(0.8- 1.3)	1.6%
Ye, M.	2018	<25.0	61	55454.5	1.1	(0.9- 1.4)	1.6%
Dow, C.	2016	<25.0	1096	954285.2	1.1	(1.1- 1.2)	1.6%
Schmidt, M.	2013	18.5-25.0	207	169672.1	1.2	(1.1- 1.4)	1.6%
Holtermann, A.	2017	<25.0	136	105426.4	1.3	(1.1- 1.5)	1.6%
Chang, Y.	2016	18.5-23.0	239	180022.1	1.3	(1.2- 1.5)	1.6%
Cuthbertson, D. J.	2021	<25.0	19	13103.4	1.4	(0.9- 2.3)	1.5%
Wannamethee, S. G.	2005	<25.0	95	59375.0	1.6	(1.3- 2.0)	1.6%
Twig, G.	2014	<25.0	173	102917.0	1.7	(1.4- 2.0)	1.6%
Maty, S. C.	2005	<25.0	154	90588.2	1.7	(1.5- 2.0)	1.6%
Meisinger, C. (F)	2006	<26.0	25	14534.0	1.7	(1.2- 2.5)	1.5%
Villegas, R.	2009	<22.7	238	129689.0	1.8	(1.6- 2.1)	1.6%
Beleigoli, A. M.	2020	<25.0	26	13832.0	1.9	(1.3- 2.8)	1.5%
Feller, S. (M)	2010	<25.0	45	23936.2	1.9	(1.4- 2.5)	1.6%
Arnlov, J.	2011	<25.0	39	18260.0	2.1	(1.6- 2.9)	1.6%
Meigs, J. B.	2006	<25.0	17	7423.6	2.3	(1.4- 3.7)	1.5%
Appleton, S. L.	2013	<25.0	16	5846.0	2.7	(1.7- 4.5)	1.5%
Krishnan, S.	2007	<25.0	150	53571.4	2.8	(2.4- 3.3)	1.6%
Ford, E. S.	1997	<25.0	113	39281.0	2.9	(2.4- 3.5)	1.6%
Li, F.	2020	<24.0	37	12333.3	3.0	(2.2- 4.1)	1.5%
Meisinger, C. (M)	2006	<25.0	21	6709.3	3.1	(2.0- 4.8)	1.5%
Aung, K.	2014	<25.0	30	8214.0	3.7	(2.6- 5.2)	1.5%
Jung, C. H.	2015	<25.0	278	75135.1	3.7	(3.3- 4.2)	1.6%
Borné, Y. (F)	2015	<24.6	316	81443.3	3.9	(3.5- 4.3)	1.6%
Feng, S.	2020	<24.0	369	90000.0	4.1	(3.7- 4.5)	1.6%
Hayashi, T.	1999	<22.7	154	36910.0	4.2	(3.6- 4.9)	1.6%
Song, B. M.	2018	<22.4	31	7342.1	4.2	(3.0- 6.0)	1.5%
Vaidya, A. (F)	2016	<23.0	121	27003.0	4.5	(3.7- 5.4)	1.6%
Lim, J. S.	2007	<25.0	34	7555.6	4.5	(3.2- 6.3)	1.5%
Borné, Y. (M)	2015	<24.0	170	36170.2	4.7	(4.0- 5.5)	1.6%
Hadaegh, F.	2006	<23.0	8	1687.8	4.7	(2.4- 9.5)	1.4%
Hinnouho, G. M.	2015	<25.0	264	54545.5	4.8	(4.3- 5.5)	1.6%
Tang, M. L.	2021	18.5-22.5	5	1005.0	5.0	(2.1-12.0)	1.3%
Jung, H. H.	2017	18.5-23.0	14494	2909177.0	5.0	(4.9- 5.1)	1.6%
Jiamjarasrangsi, W.	2005	18.5-23.0	23	4339.6	5.3	(3.5- 8.0)	1.5%
Chen, Y.	2021	<24.0	59	11028.0	5.3	(4.1- 6.9)	1.6%
Uemura, M.	2015	<25.0	176	32592.6	5.4	(4.7- 6.3)	1.6%
Han, C.	2017	<23.3	152	28029.8	5.4	(4.6- 6.4)	1.6%
Edwards, M. K.	2017	18.5-25.0	16	2909.1	5.5	(3.4- 9.0)	1.5%
Wang, B.	2017	18.5-24.0	193	34464.3	5.6	(4.9- 6.4)	1.6%
Tatsumi, Y. (F)	2012	18.5-23.0	121	19836.1	6.1	(5.1- 7.3)	1.6%
Xu, S.	2021	<24	39	5803.6	6.7	(4.9- 9.2)	1.6%
Vaidya, A. (M)	2016	<23.0	672	72385.0	9.3	(8.6-10.0)	1.6%
Tatsumi, Y. (M)	2012	18.5-23.0	106	10600.0	10.0	(8.3-12.1)	1.6%
Wei, Y.	2020	<24.0	399	37464.8	10.7	(9.7-11.7)	1.6%
Ishikawa-Takata, K.	2002	<23.0	108	9908.3	10.9	(9.0-13.2)	1.6%
Janghorbani, M.	2017	<25.0	36	3103.4	11.6	(8.4-16.1)	1.5%
Asghar, S. (F)	2011	18.5-23.0	48	3333.3	14.4	(10.9-19.1)	1.6%
Jung, J. Y. (F)	2018	18.5-23.0	148	9897.0	15.0	(12.7-17.6)	1.6%
Sun, J. (Urban)	2019	18.5-24.0	17	1135.7	15.0	(9.3-24.1)	1.5%
Asghar, S. (M)	2011	18.5-23.0	29	1859.0	15.6	(10.8-22.4)	1.5%
Sun, J. (ARural)	2019	18.5-24.0	65	3718.7	17.5	(13.7-22.3)	1.6%
Jung, J. Y. (M)	2018	18.5-23.0	186	9483.0	19.6	(17.0-22.6)	1.6%
Wang, H.	2010	<25.0	23	754.1	30.5	(20.3-45.9)	1.5%
Zhu, X.	2021	18.5-24	125	2100.8	59.5	(49.9-70.9)	1.6%
**Random effects model**					**3.3**	**(2.6- 4.3)**	**100.0%**

Heterogeneity: $I^2 = 99.5\%$, $\tau = 0.9695$, $p < 0.001$

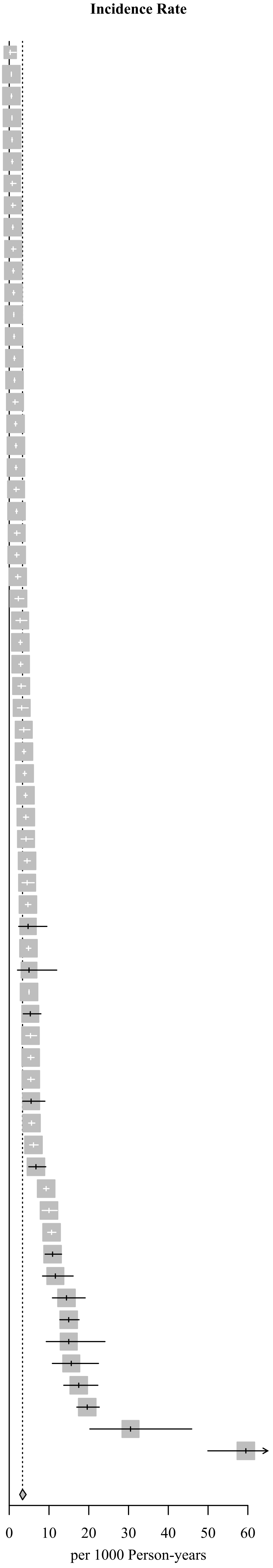

Figure S2

C. Overweight/obesity

Study	Year	BMI	Cases	Personyears	Rate	95%-CI	Weight
Hu, H.	2017	>23.0	2501	1807005.6	1.4	(1.3- 1.4)	1.2%
Bardenheier, B. H.	2021	>25.0	41	20499.0	2.0	(1.5- 2.7)	1.1%
Chang, Y.	2016	>23.0	213	91976.8	2.3	(2.0- 2.6)	1.2%
Ohlsson, C.	2019	>25.0	234	75483.9	3.1	(2.7- 3.5)	1.2%
Sui, X.	2008	>25.0	56	17073.0	3.3	(2.5- 4.3)	1.1%
Holtermann, A.	2017	>25.0	382	114396.1	3.3	(3.0- 3.7)	1.2%
Hu, G.	2004	>25.0	109	29092.0	3.7	(3.1- 4.5)	1.2%
Maty, S. C.	2005	>25.0	164	42000.0	3.9	(3.4- 4.6)	1.2%
Fox, C. S.	2006	>25.0	29	6736.8	4.3	(3.0- 6.2)	1.1%
Hodge, A. M.	2021	>25.0	1116	247240.0	4.5	(4.3- 4.8)	1.2%
Schmidt, M.	2013	>25.0	102	22386.7	4.6	(3.8- 5.5)	1.2%
Cuthbertson, D. J.	2021	>25.0	33	7096.8	4.7	(3.3- 6.5)	1.1%
Dow, C.	2016	>25.0	1514	298373.4	5.1	(4.8- 5.3)	1.2%
Dotevall, A.	2004	>24.0	60	11618.8	5.2	(4.0- 6.7)	1.2%
Papier, K. (F)	2017	>23.0	199	38125.9	5.2	(4.5- 6.0)	1.2%
Twig, G.	2014	>25.0	561	107362.0	5.2	(4.8- 5.7)	1.2%
Papier, K. (M)	2017	>23.0	358	65676.3	5.5	(4.9- 6.0)	1.2%
Wannamethee, S. G.	2005	>25.0	354	63355.1	5.6	(5.0- 6.2)	1.2%
Bragg, F. (M)	2018	>25.0	2797	481168.2	5.8	(5.6- 6.0)	1.2%
Bragg, F. (F)	2018	>25.0	4658	789271.5	5.9	(5.7- 6.1)	1.2%
Feng, S.	2020	>24.0	674	107830.0	6.3	(5.8- 6.7)	1.2%
Feller, S. (F)	2010	>25.0	377	59699.2	6.3	(5.7- 7.0)	1.2%
Appleton, S. L.	2013	>25.0	96	13279.9	7.2	(5.9- 8.8)	1.2%
Feller, S. (M)	2010	>25.0	2544	345262.8	7.4	(7.1- 7.7)	1.2%
Ye, M.	2018	>25.0	947	125411.7	7.6	(7.1- 8.0)	1.2%
Beleigoli, A. M.	2020	>25.0	210	26639.9	7.9	(6.9- 9.0)	1.2%
Arnlov, J.	2011	>25.0	121	15240.0	7.9	(6.6- 9.5)	1.2%
Villegas, R.	2009	>22.7	1362	167580.0	8.1	(7.7- 8.6)	1.2%
Weinstein, A. R.	2004	>25.0	1183	126318.7	9.4	(8.8- 9.9)	1.2%
Meigs, J. B.	2006	>25.0	124	12998.3	9.5	(8.0-11.4)	1.2%
Borné, Y. (F)	2015	>24.6	1100	113281.7	9.7	(9.2-10.3)	1.2%
Meisinger, C. (F)	2006	>26.1	133	13505.0	9.8	(8.3-11.7)	1.2%
Tang, M. L.	2021	>22.5	331	33550.0	9.9	(8.9-11.0)	1.2%
Hu, G.	2005	>30.0	72	6923.1	10.4	(8.3-13.1)	1.2%
Ford, E. S.	1997	>25.0	316	29537.0	10.7	(9.6-11.9)	1.2%
Meisinger, C. (M)	2006	>25.1	222	20526.0	10.8	(9.5-12.3)	1.2%
Jung, C. H.	2015	>25.0	357	32844.0	10.9	(9.8-12.1)	1.2%
Hadaegh, F.	2006	>23.0	61	5374.9	11.3	(8.8-14.6)	1.2%
Tatsumi, Y. (F)	2012	>23.0	278	24451.2	11.4	(10.1-12.8)	1.2%
Hayashi, T.	1999	>22.7	416	36036.0	11.5	(10.5-12.7)	1.2%
Chen, Y.	2021	>24.0	39	3356.3	11.6	(8.5-15.9)	1.1%
Lim, J. S.	2007	>25.0	171	14305.9	12.0	(10.3-13.9)	1.2%
Uemura, M.	2015	>25.0	109	9008.3	12.1	(10.0-14.6)	1.2%
Jae, S. Y.	2016	>25.0	90	7260.9	12.4	(10.1-15.2)	1.2%
Song, B. M.	2018	>22.4	273	21323.6	12.8	(11.4-14.4)	1.2%
Li, F.	2020	>24.0	133	10382.4	12.8	(10.8-15.2)	1.2%
Jung, H. H.	2017	>23.0	58749	4392513.0	13.4	(13.3-13.5)	1.2%
Borné, Y. (M)	2015	>24.0	1349	98535.8	13.7	(13.0-14.4)	1.2%
Asvold, B. O.	2017	>30.0	548	40000.0	13.7	(12.6-14.9)	1.2%
Hinnouho, G. M.	2015	>25.0	534	38385.0	13.9	(12.8-15.1)	1.2%
Wang, B.	2017	>24.0	529	36757.3	14.4	(13.2-15.7)	1.2%
Han, C.	2017	>23.3	597	41189.9	14.5	(13.4-15.7)	1.2%
Sasai, H. (F)	2010	>25.0	1031	68087.0	15.1	(14.2-16.1)	1.2%
Xu, S.	2021	>24.0	74	4833.4	15.3	(12.2-19.2)	1.2%
Sairenchi, T. (F)	2007	>25.0	2001	128474.0	15.6	(14.9-16.3)	1.2%
Vaidya, A. (F)	2016	>23.0	631	40191.1	15.7	(14.5-17.0)	1.2%
Gautier, A. (F)	2010	>27.0	40	2500.0	16.0	(11.7-21.8)	1.1%
Gautier, A. (M)	2010	>27.0	71	4437.5	16.0	(12.7-20.2)	1.2%
Tatsumi, Y. (M)	2012	>23.0	177	11004.0	16.1	(13.9-18.6)	1.2%
Edwards, M. K.	2017	>25.0	267	16441.7	16.2	(14.4-18.3)	1.2%
Fujita, M. (F, Chiba)	2009	>25.0	743	44505.0	16.7	(15.5-17.9)	1.2%
Jiamjarasrangsi, W.	2005	23.0-27.5	90	5310.0	16.9	(13.8-20.8)	1.2%
Aung, K.	2014	>25.0	232	12609.6	18.4	(16.2-20.9)	1.2%
Asghar, S. (F)	2011	>23.0	29	1559.1	18.6	(12.9-26.8)	1.1%
Vaidya, A. (M)	2016	>23.0	3417	181755.3	18.8	(18.2-19.4)	1.2%
Janghorbani, M.	2017	>25.0	327	16639.0	19.7	(17.6-21.9)	1.2%
Fujita, M. (F, Kashiwa)	2009	>25.0	285	13828.0	20.6	(18.4-23.1)	1.2%
Ishikawa-Takata, K.	2002	>23.0	135	6532.2	20.7	(17.5-24.5)	1.2%
Sun, J. (Rural)	2019	>24.0	126	5686.7	22.2	(18.6-26.4)	1.2%
Jung, J. Y. (F)	2018	>23.0	557	23656.0	23.5	(21.7-25.6)	1.2%
Wei, Y.	2020	>24.0	1054	44447.7	23.7	(22.3-25.2)	1.2%
Sasai, H. (M)	2010	>25.0	701	27157.0	25.8	(24.0-27.8)	1.2%
Sairenchi, T. (M)	2007	>25.0	1260	48622.0	25.9	(24.5-27.4)	1.2%
Jung, J. Y. (M)	2018	>23.0	521	19605.0	26.6	(24.4-29.0)	1.2%
Asghar, S. (M)	2011	>23.0	18	676.7	26.6	(16.8-42.2)	1.1%
Fujita, M. (M, Chiba)	2009	>25.0	388	14323.0	27.1	(24.5-29.9)	1.2%
Nagayoshi, M.	2016	>30.0	131	4729.2	27.7	(23.3-32.9)	1.2%
Chan, J. C. Y. (F)	2018	>25.0	133	4585.4	29.0	(24.5-34.4)	1.2%
Chan, J. C. Y. (M)	2018	>25.0	102	3433.2	29.7	(24.5-36.1)	1.2%
Sun, J. (Urban)	2019	>24.0	65	1894.2	34.3	(26.9-43.8)	1.2%
Fujita, M. (M, Kashiwa)	2009	>25.0	229	6186.0	37.0	(32.5-42.1)	1.2%
Giraldez-Garcia, C.	2018	>30.0	82	1444.0	56.8	(45.7-70.5)	1.2%
Ning, F.	2020	>30.0	54	834.6	64.7	(49.6-84.5)	1.1%
Wang, H.	2010	>25.0	370	5189.9	71.3	(64.4-78.9)	1.2%
Zhu, X.	2021	>24.0	290	3880.0	74.7	(66.6-83.9)	1.2%
**Random effects model**					**11.5**	**(10.0-13.2)**	**100.0%**

Heterogeneity: $I^2 = 99.7\%$, $\tau = 0.6503$, $p < 0.001$

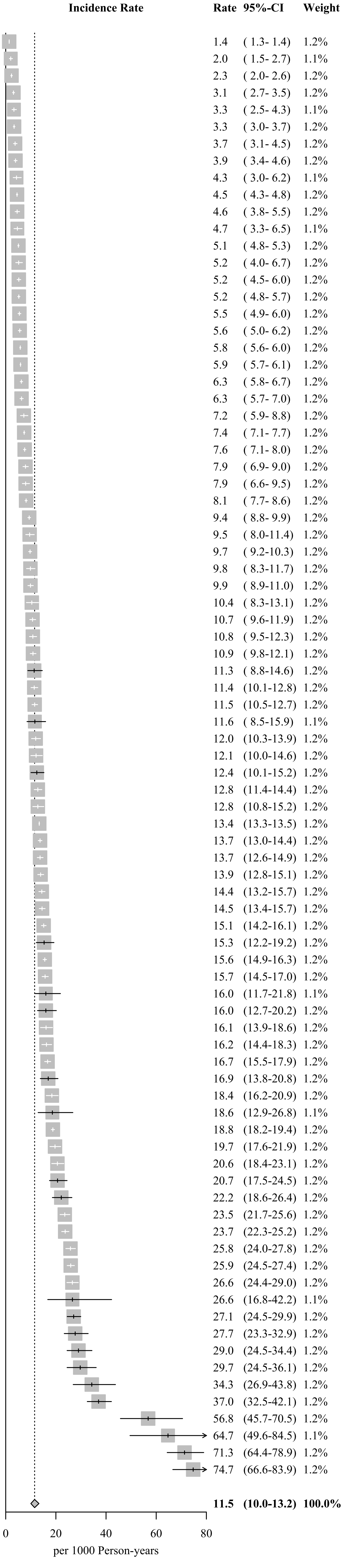

Figure S3

A. Underweight

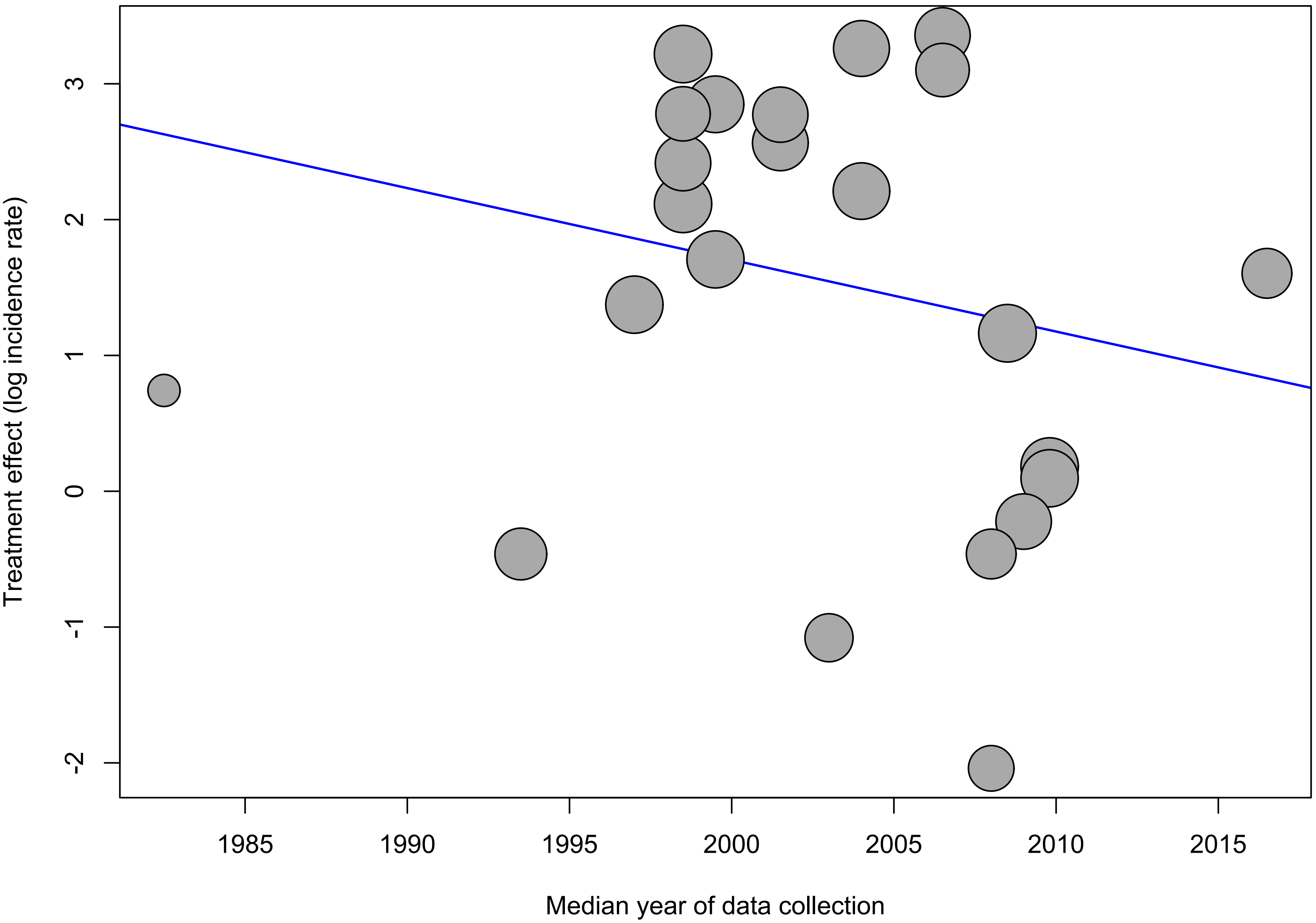

Figure S3

B. Normal weight

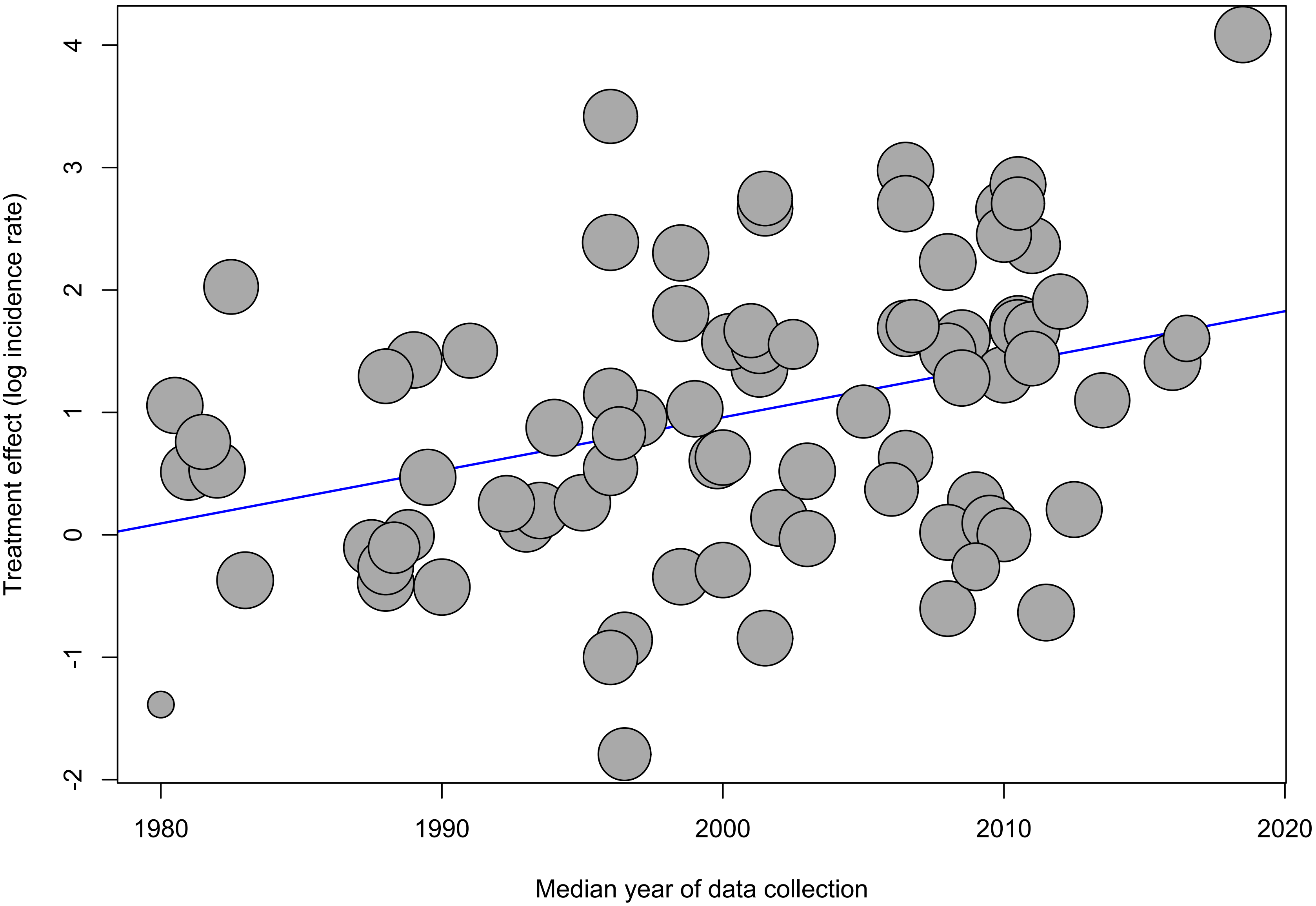

Figure S3

C. Overweight/obesity

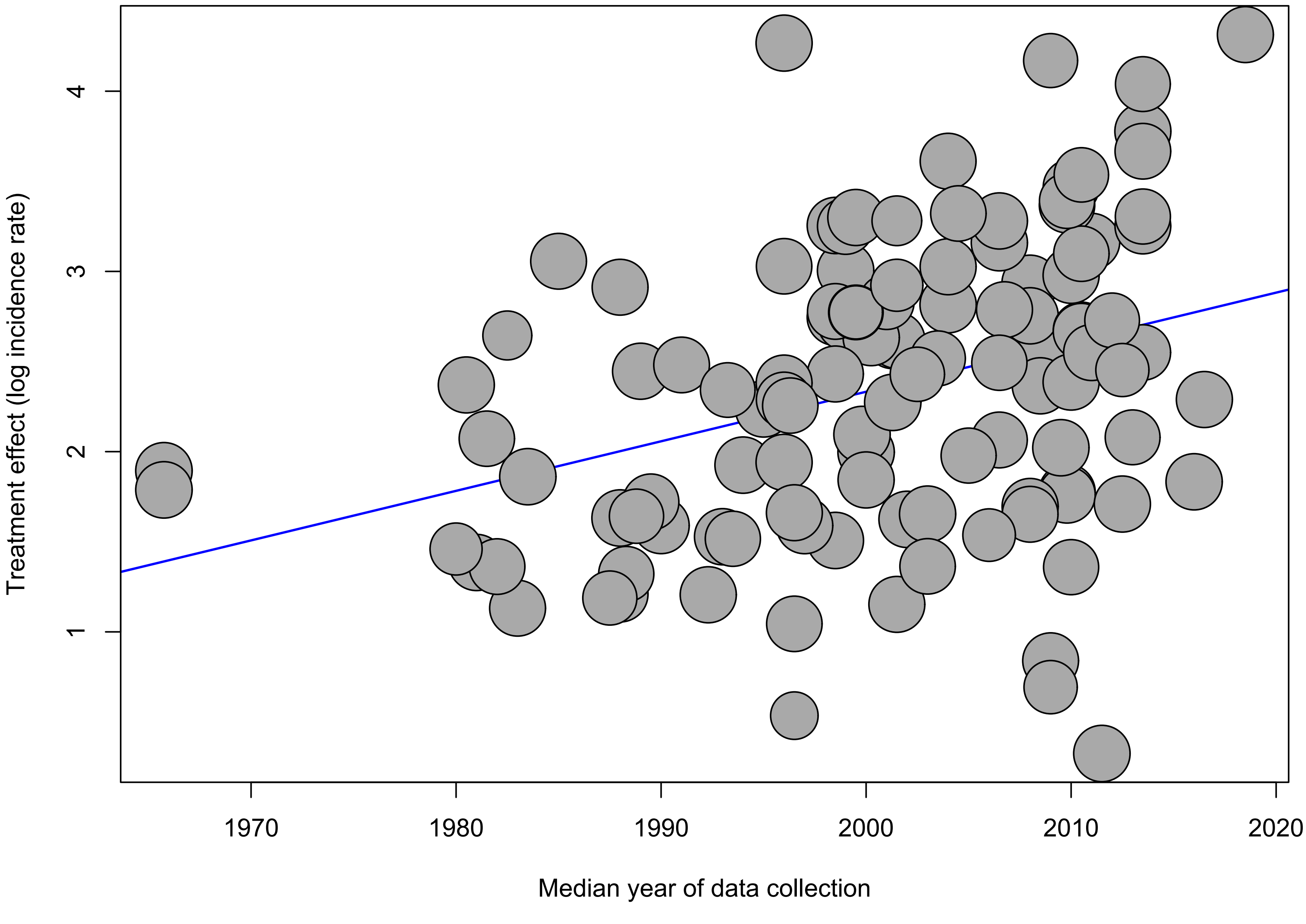

Figure S4

A. Underweight

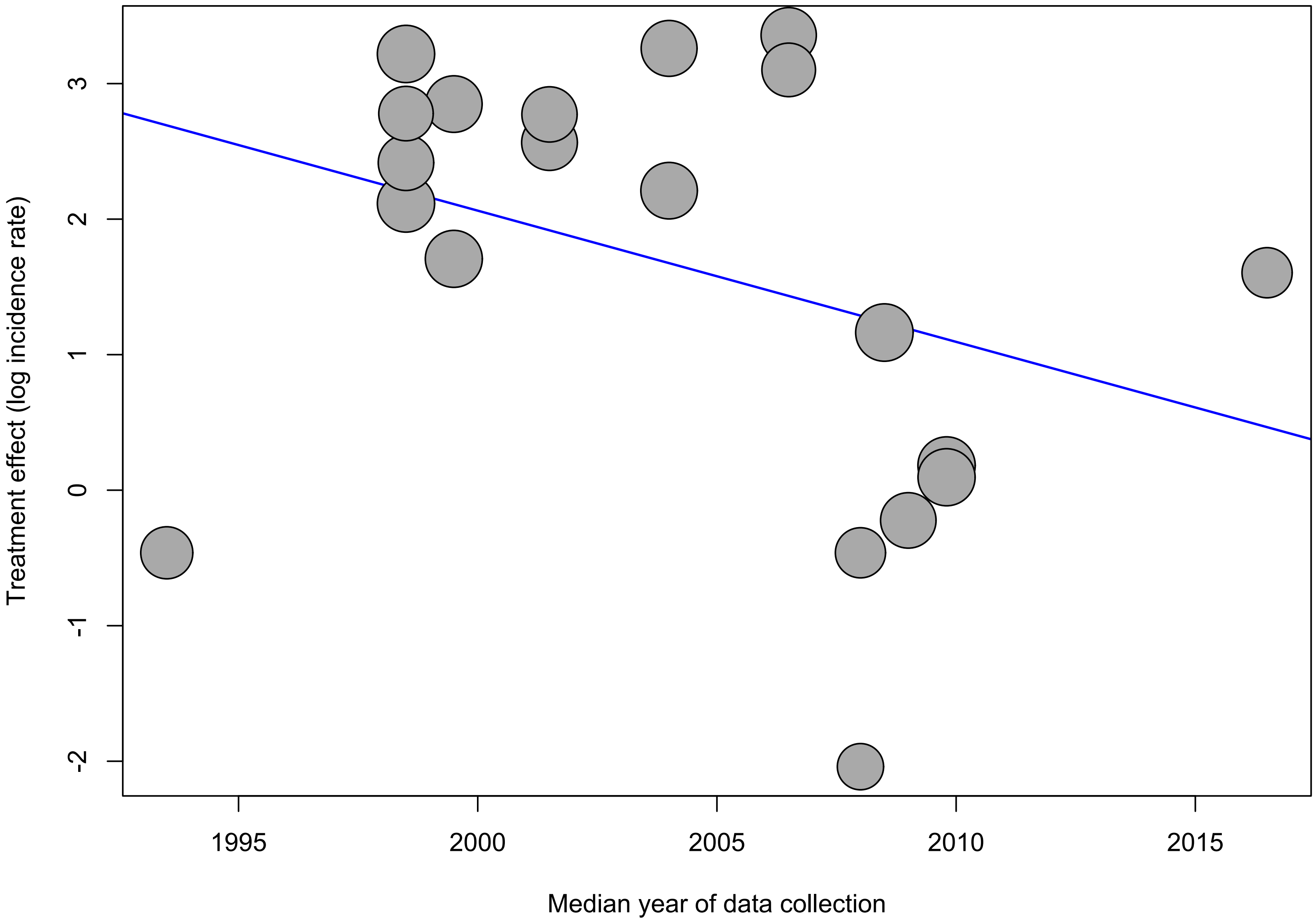

Figure S4

B. Normal weight

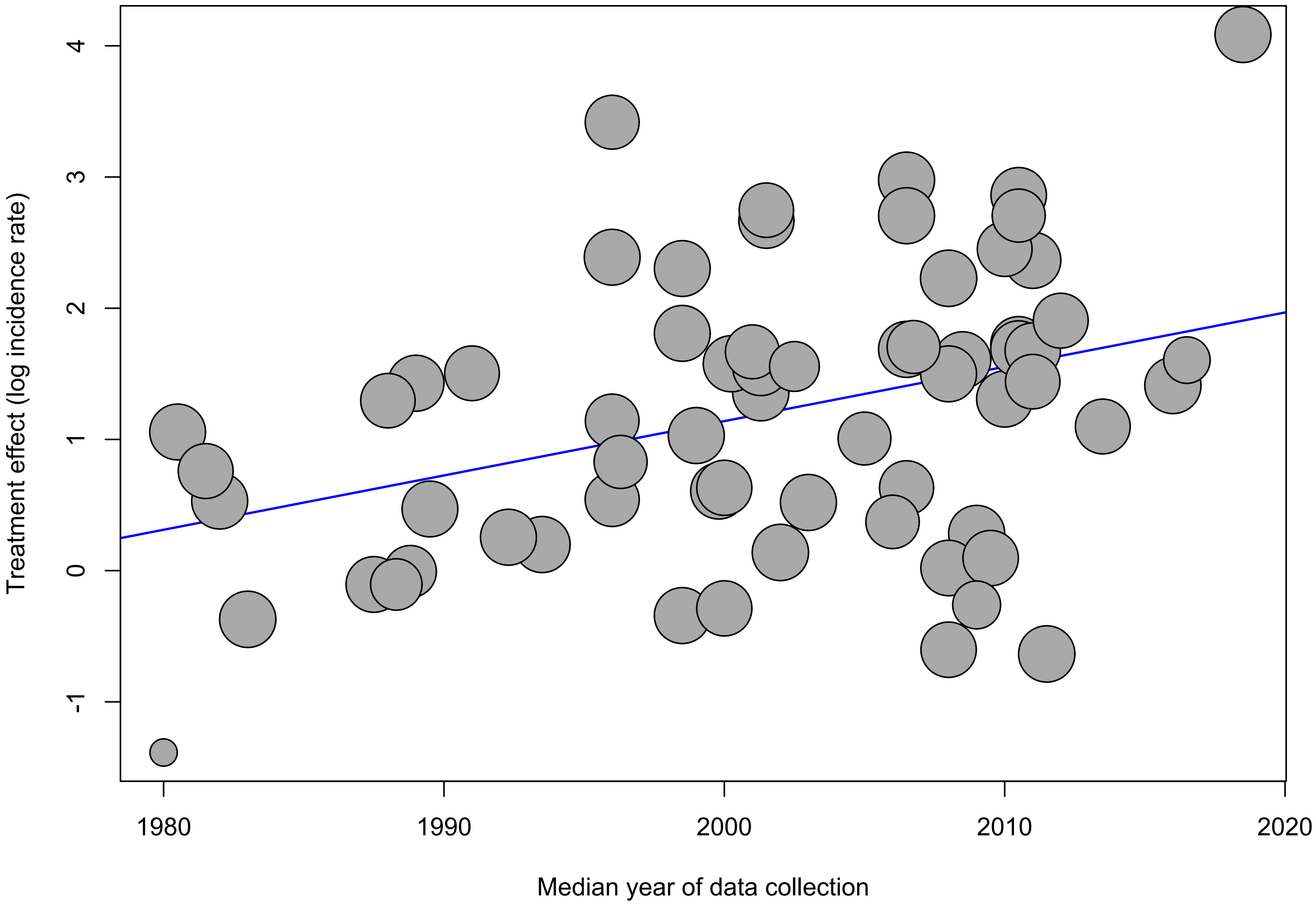

Figure S4

C. Overweight/obesity

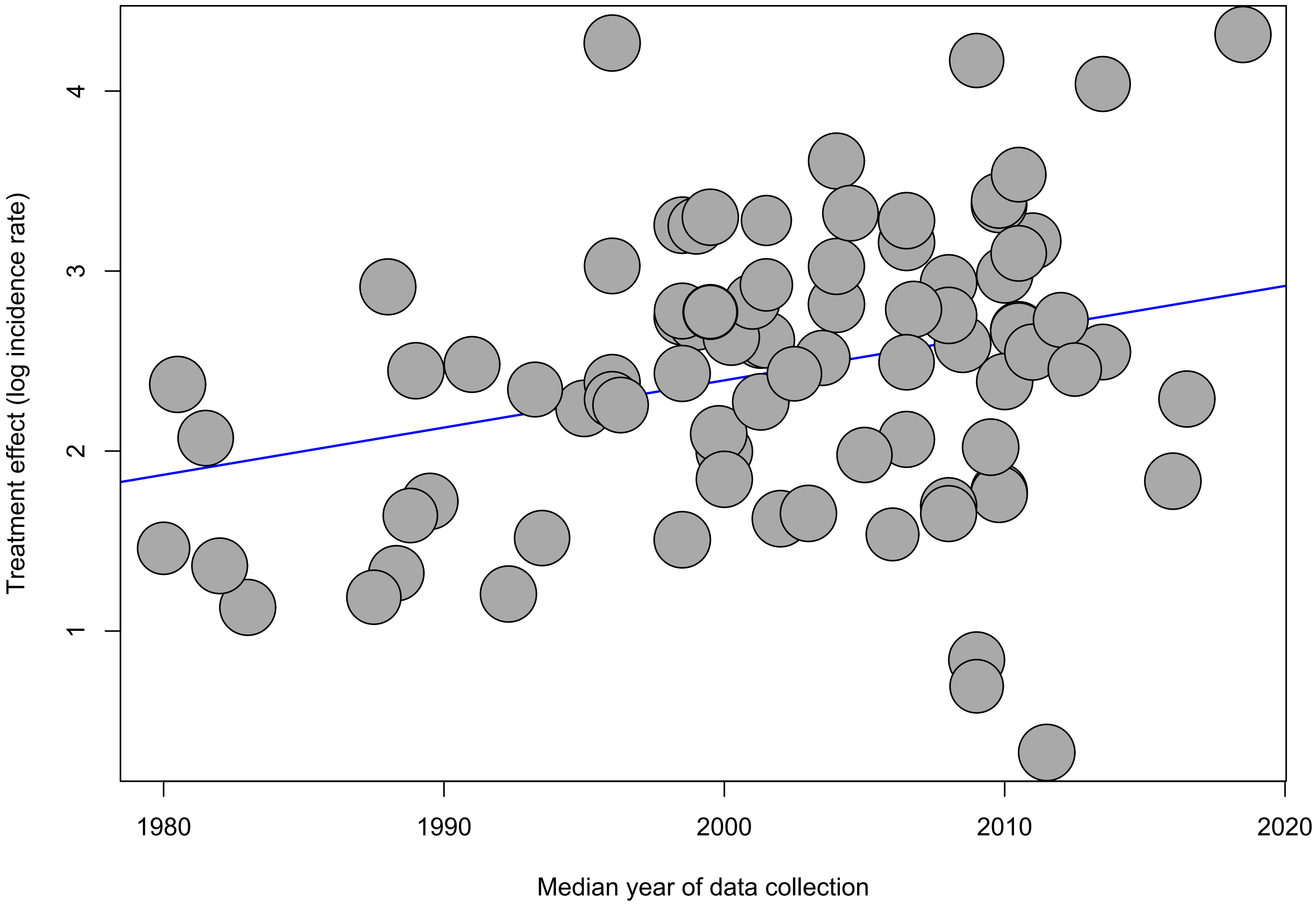

Figure S5

A. Underweight

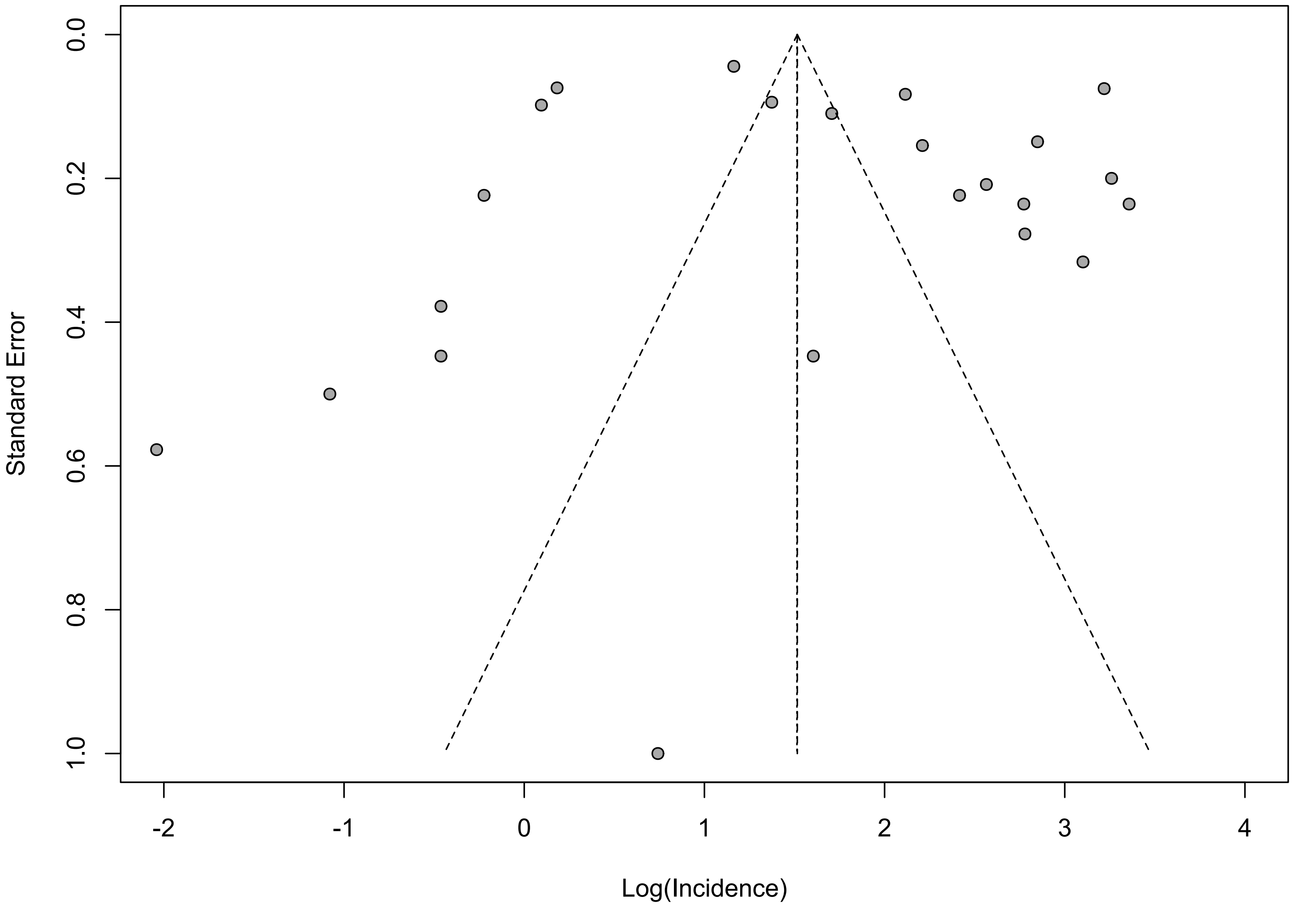

Figure S5

B. Normal weight

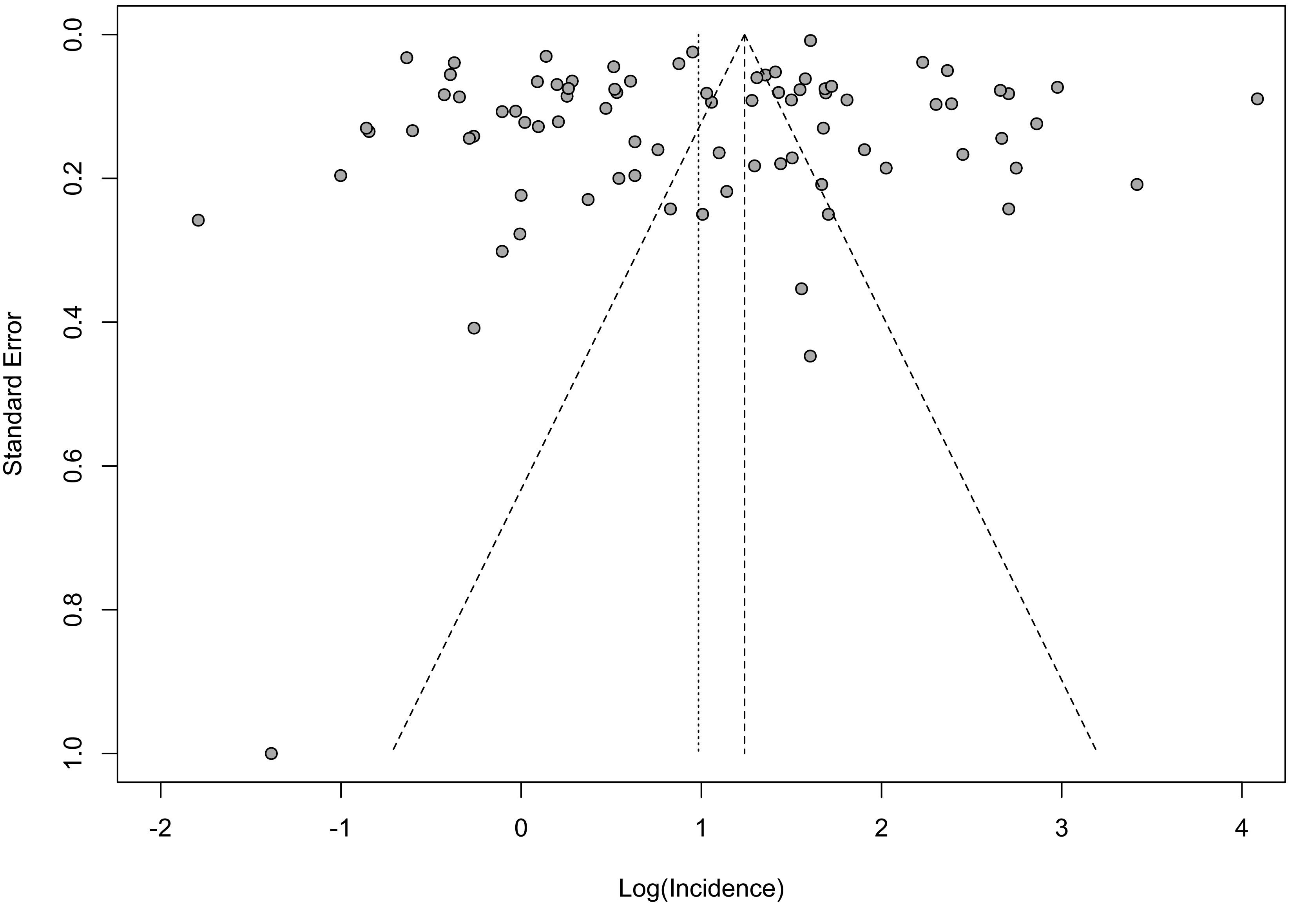

Figure S5

C. Overweight/obesity

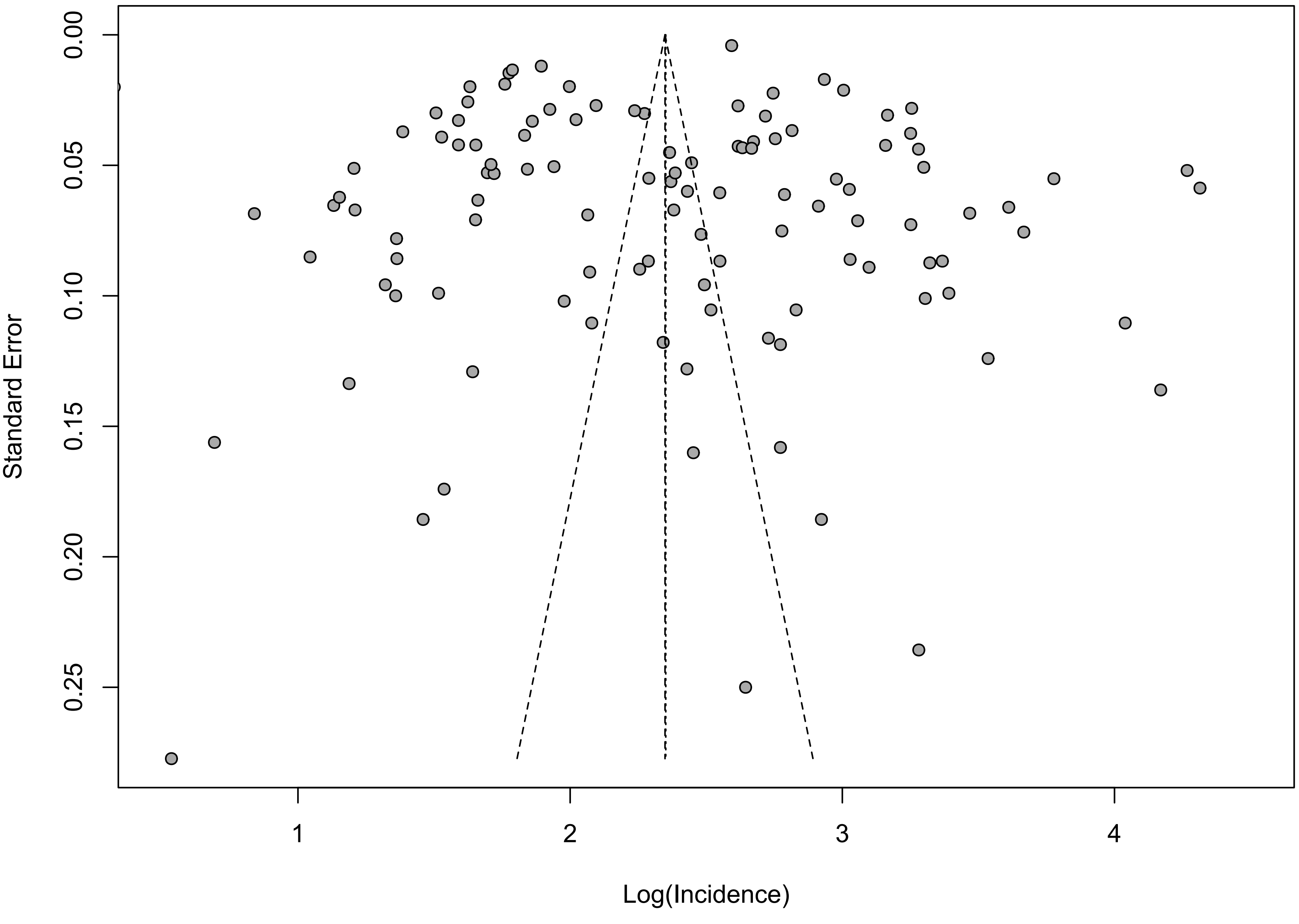

Supplement: Online Supplementary Document [file jogh-13-04088-s001.pdf]
